# Supplementary figures and images for: Dysfunctional S1P/S1PR1 signaling in the dentate gyrus drives vulnerability of chronic pain-related memory impairment
Source: eLife. 2024 Dec 19;13:RP99862. doi: 10.7554/eLife.99862 (PMC11658773; doi:10.7554/eLife.99862)

Full unedited blot for Figure 2F

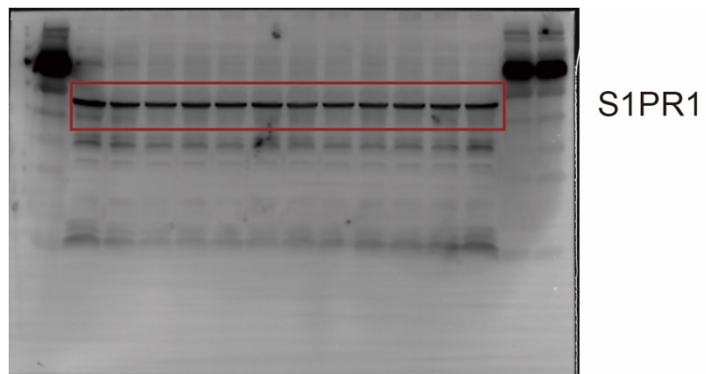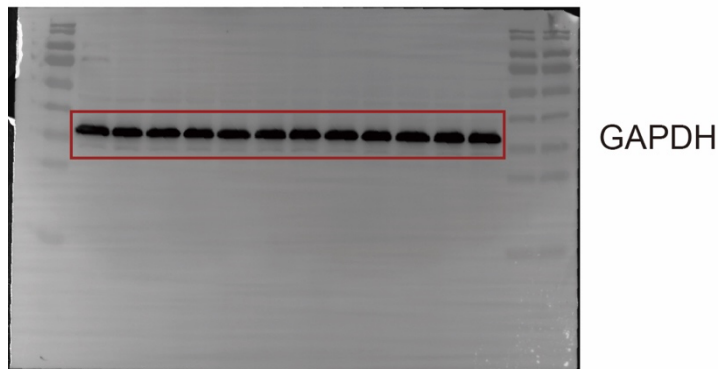

Full unedited blot for Figure 2G

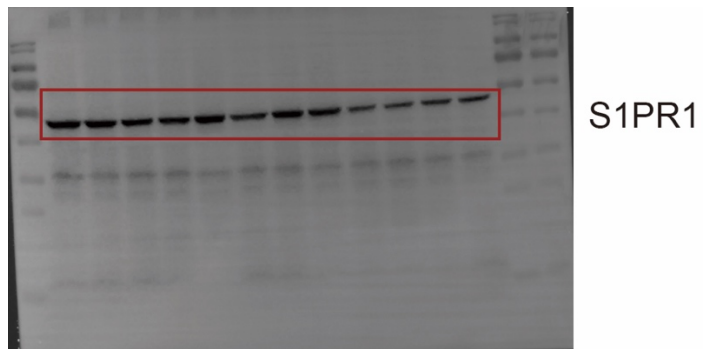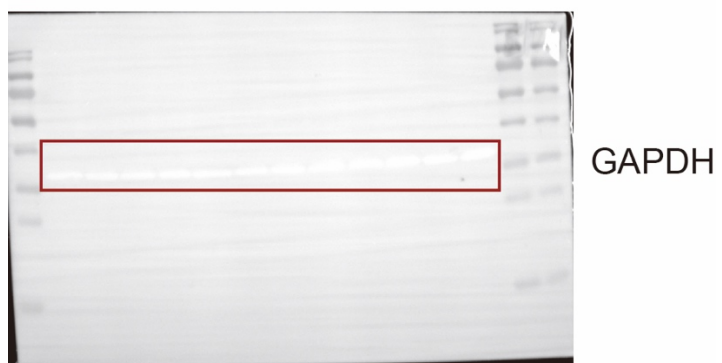

Supplement: Figure 2—source data 1. [file elife-99862-fig2-data1.zip › Figure 2-source data 1.pdf]

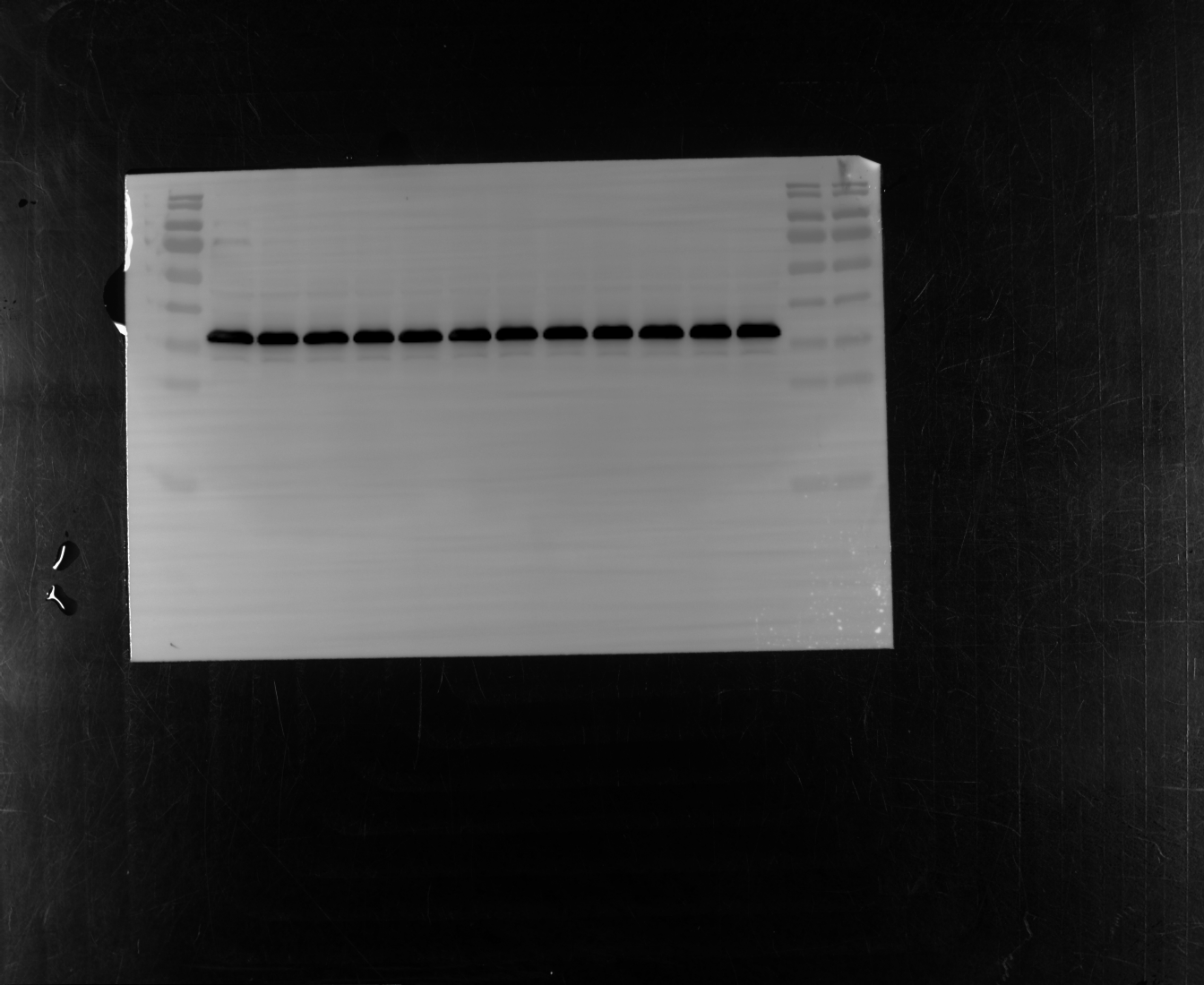

Supplement: Figure 2—source data 2. [file elife-99862-fig2-data2.zip › Figure 2-source data 2/GAPDH-Figure 2F.Tif]

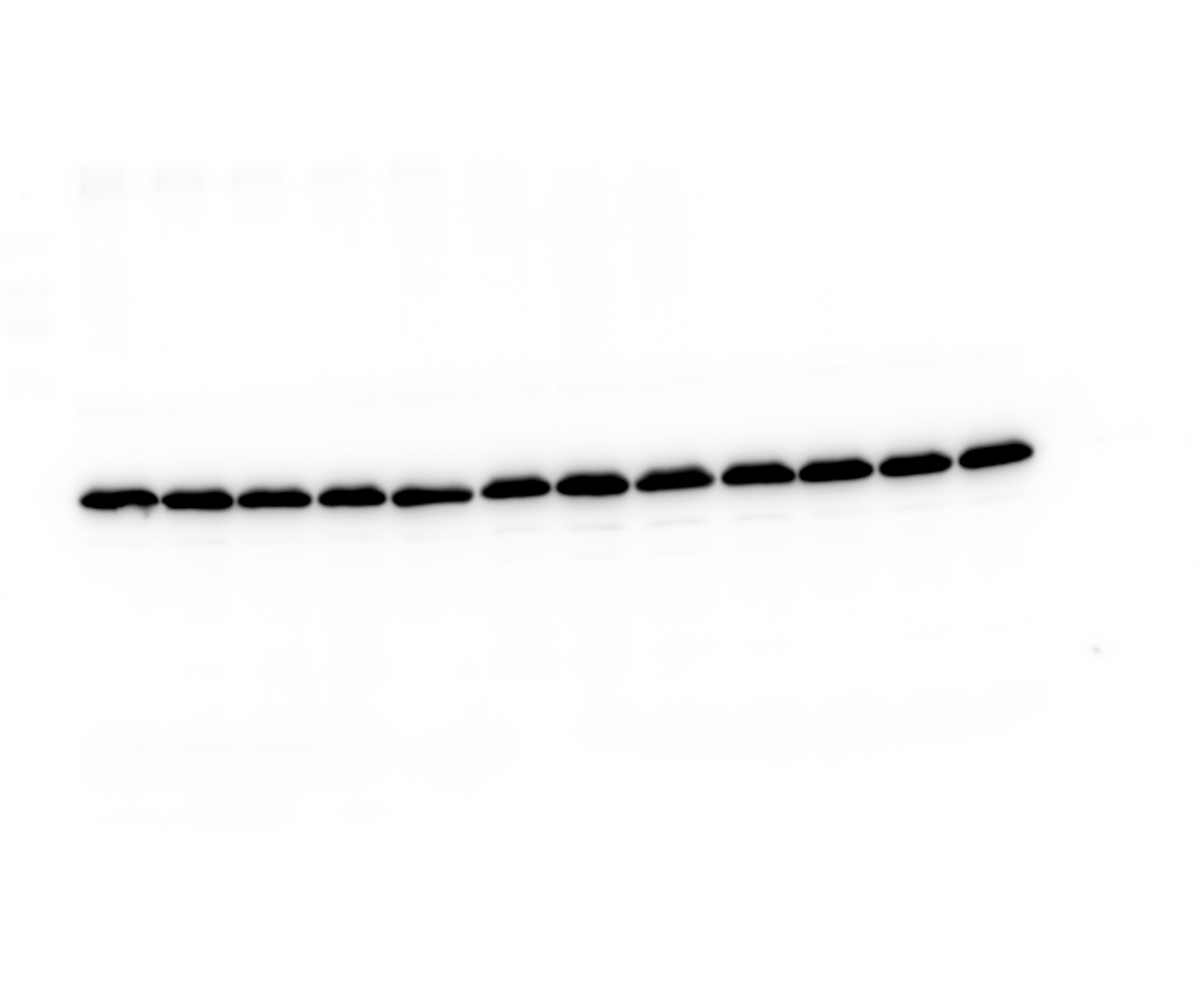

Supplement: Figure 2—source data 2. [file elife-99862-fig2-data2.zip › Figure 2-source data 2/GAPDH-Figure 2G.Tif]

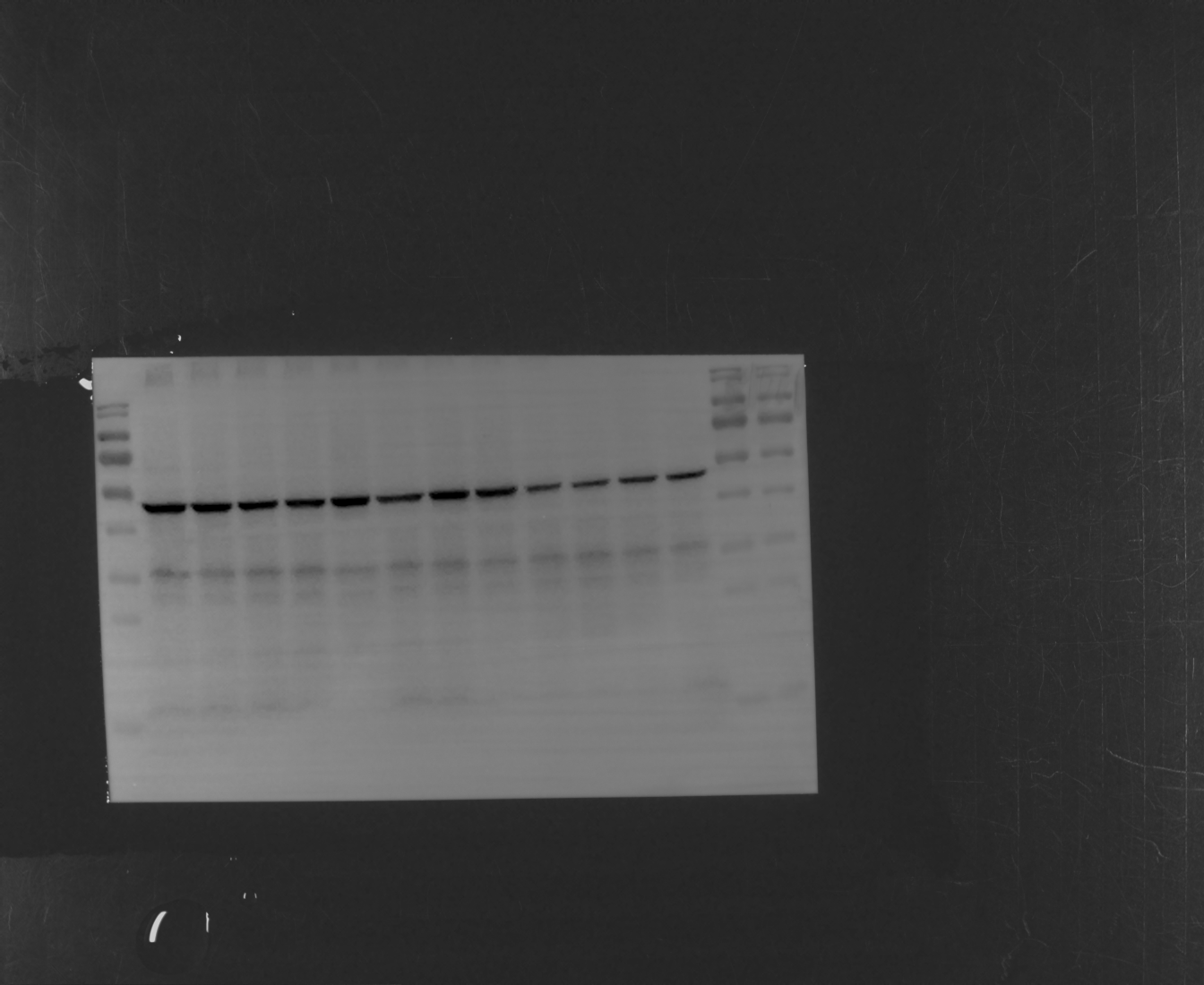

Supplement: Figure 2—source data 2. [file elife-99862-fig2-data2.zip › Figure 2-source data 2/S1PR1-Figure 2G.Tif]

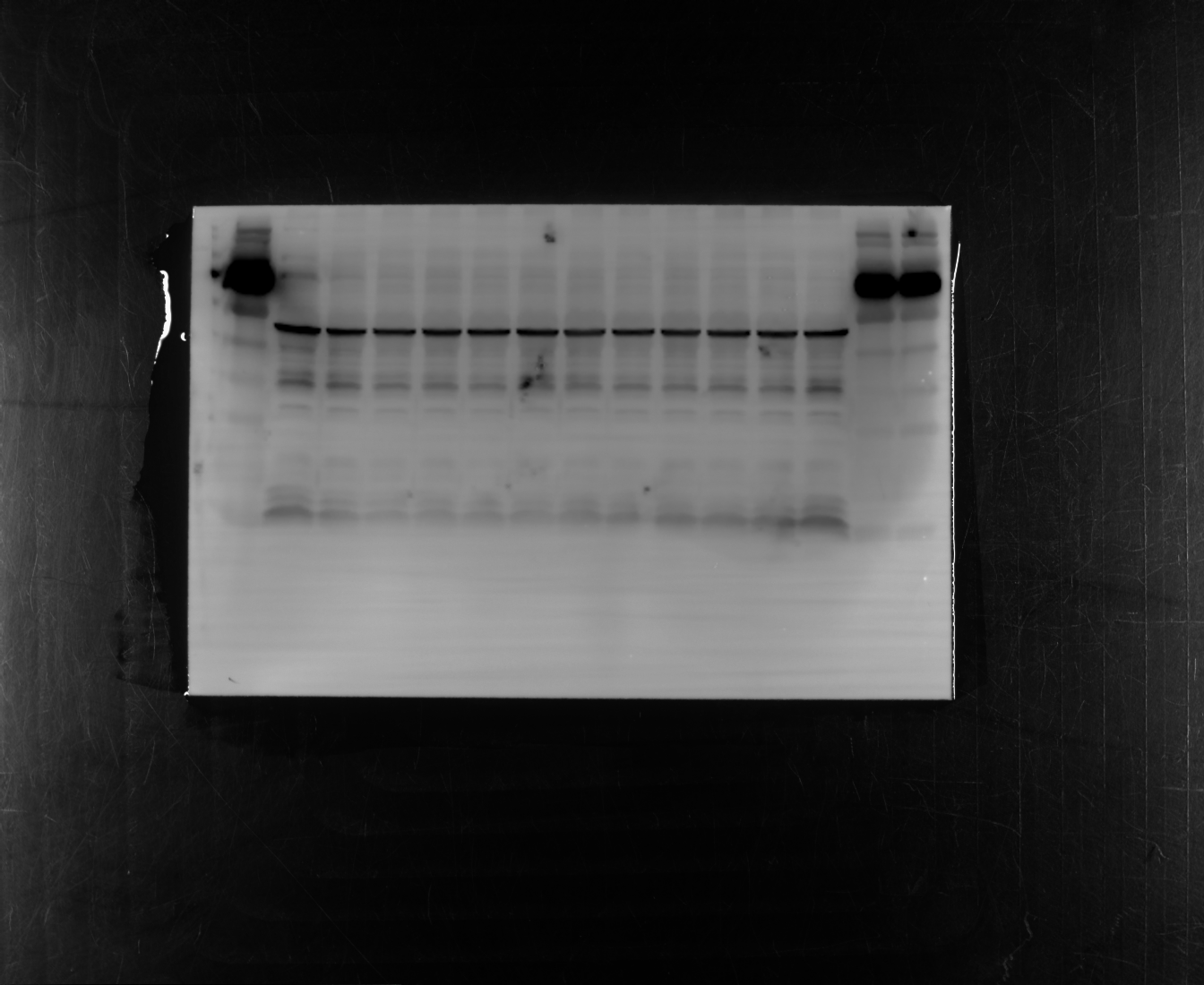

Supplement: Figure 2—source data 2. [file elife-99862-fig2-data2.zip › Figure 2-source data 2/S1PR1-Figure 2F.Tif]

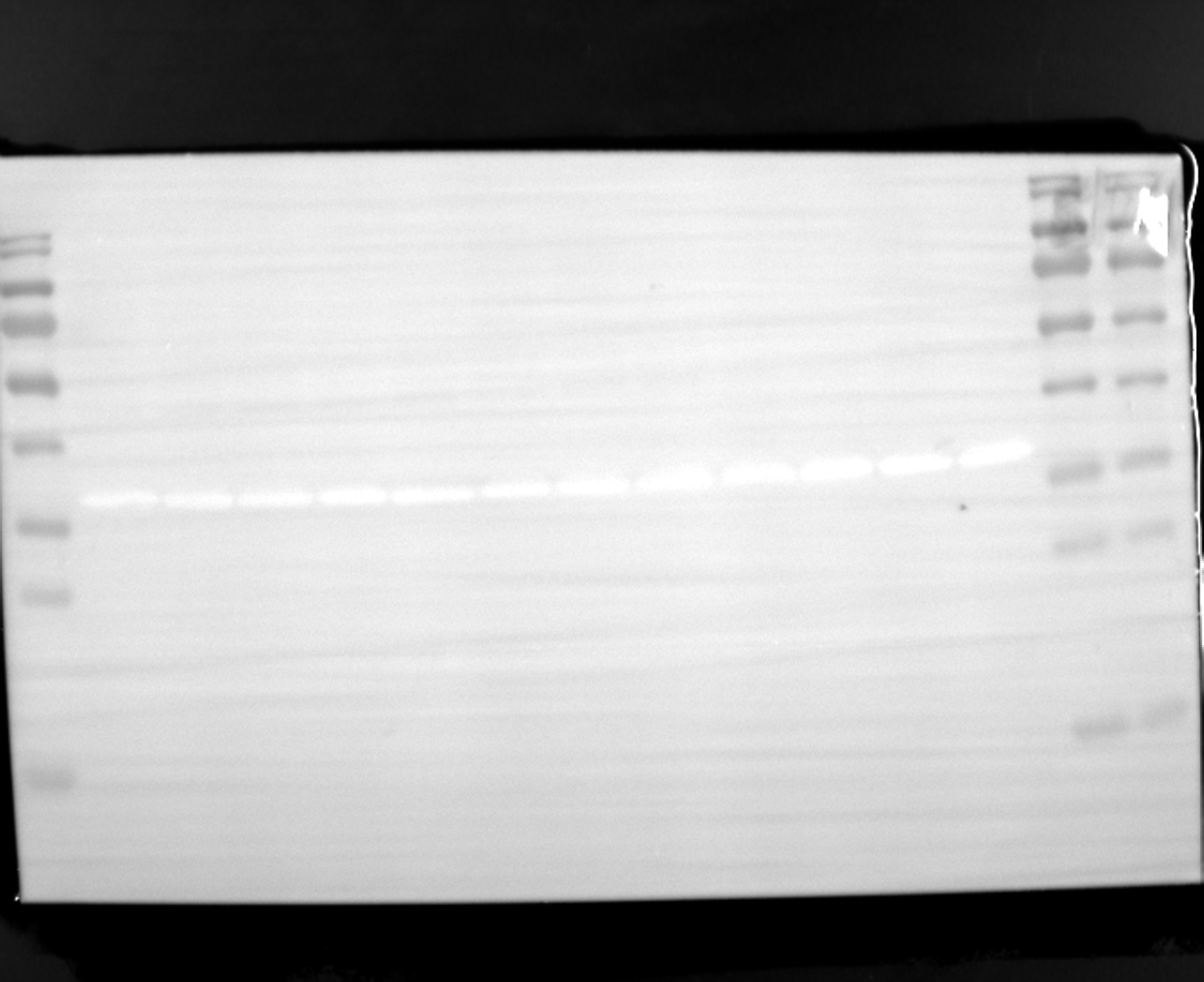

Supplement: Figure 2—source data 2. [file elife-99862-fig2-data2.zip › Figure 2-source data 2/GAPDH-Figure 2G-BF.Tif]

Full unedited blot for Figure 4C

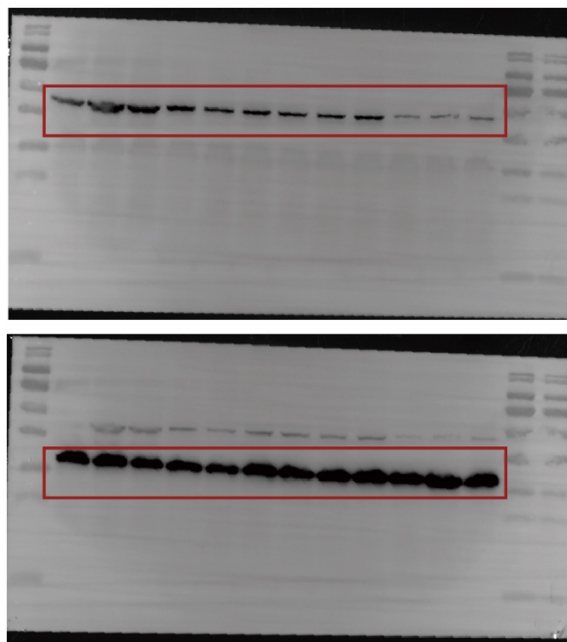

S1PR1

GAPDH

Supplement: Figure 3—source data 1. [file elife-99862-fig3-data1.pdf]

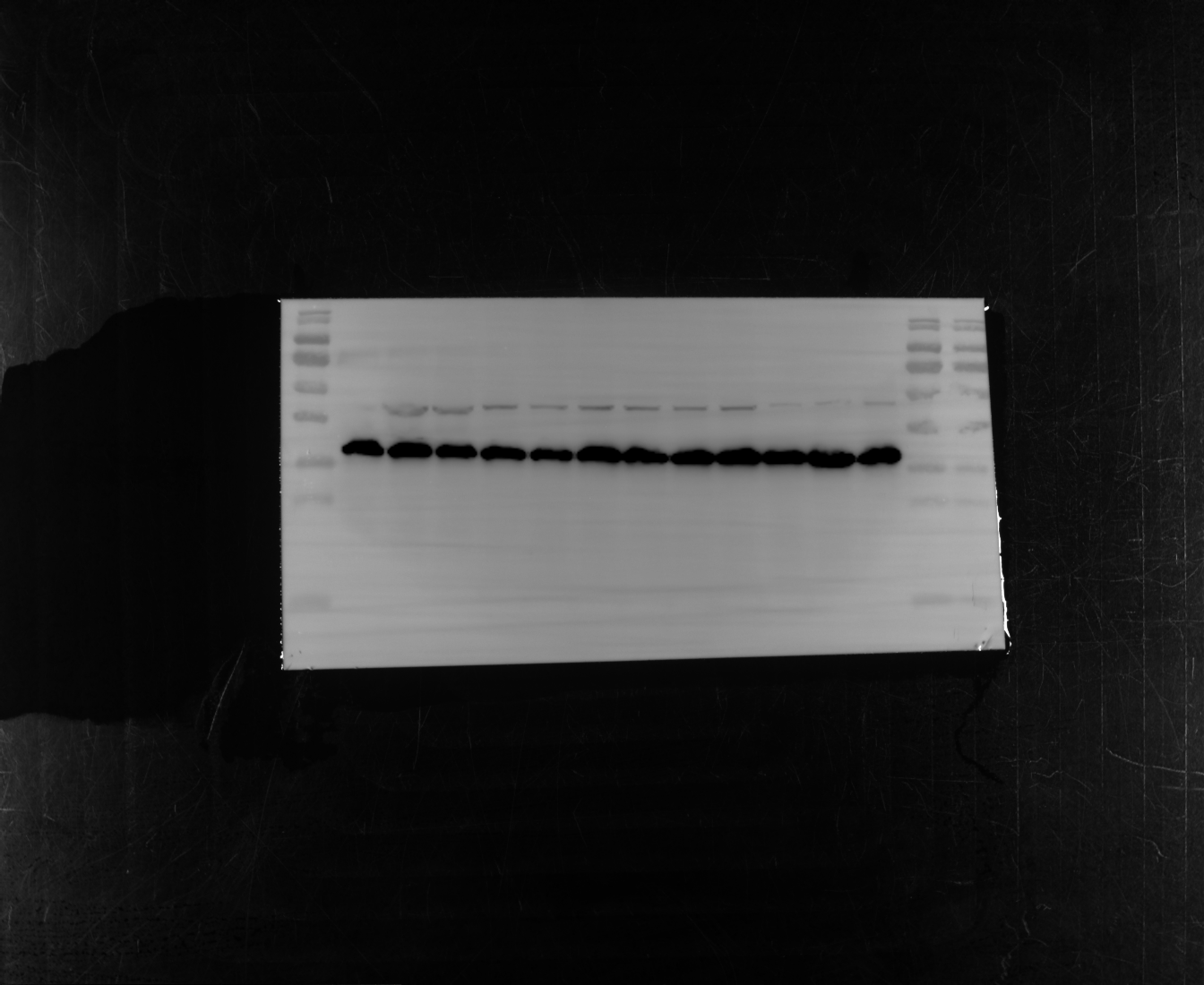

Supplement: Figure 3—source data 2. [file elife-99862-fig3-data2.zip › Figure 3-source data 2/GAPDH.Tif]

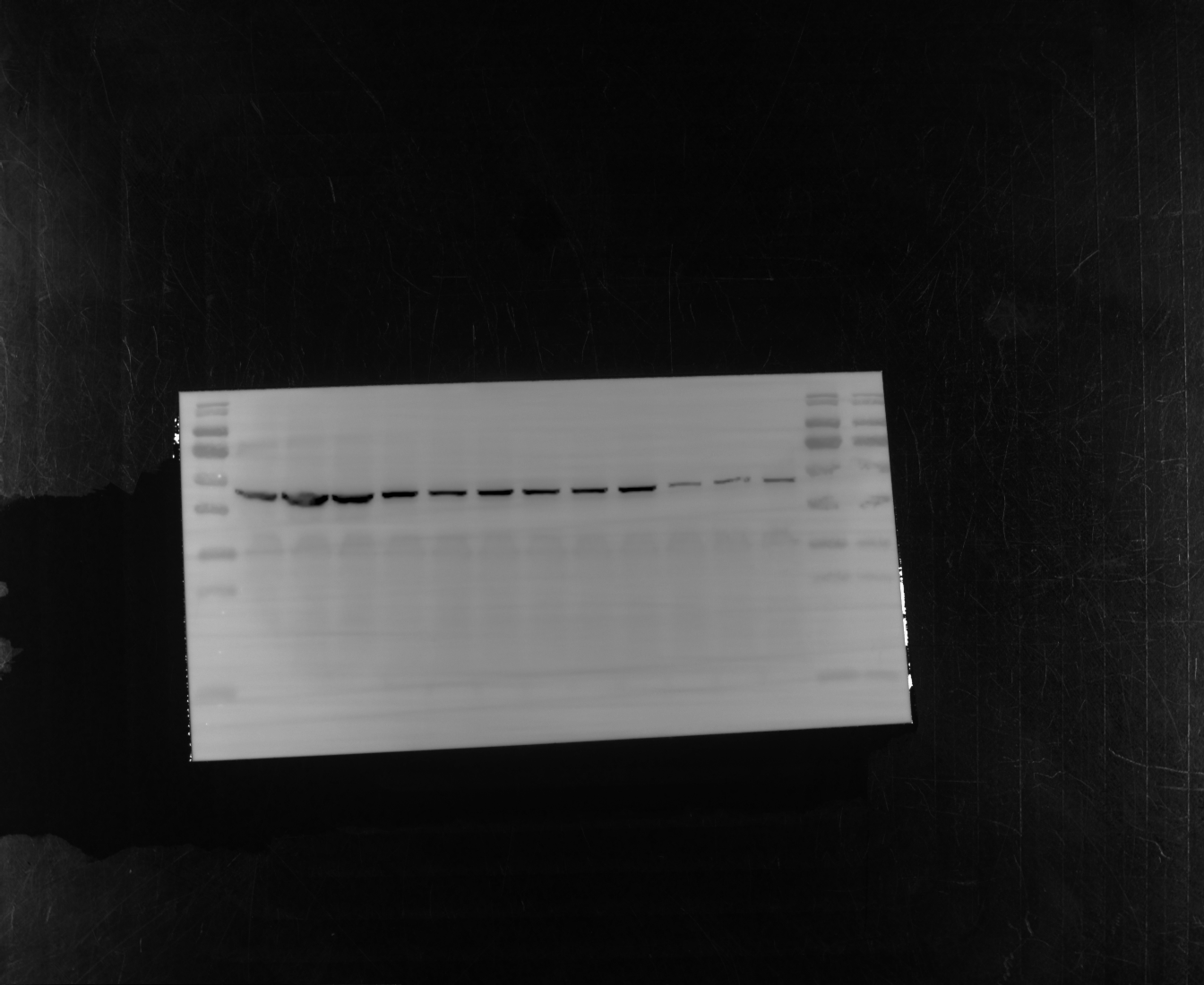

Supplement: Figure 3—source data 2. [file elife-99862-fig3-data2.zip › Figure 3-source data 2/s1pr1.Tif]

Full unedited blot for Figure 4C

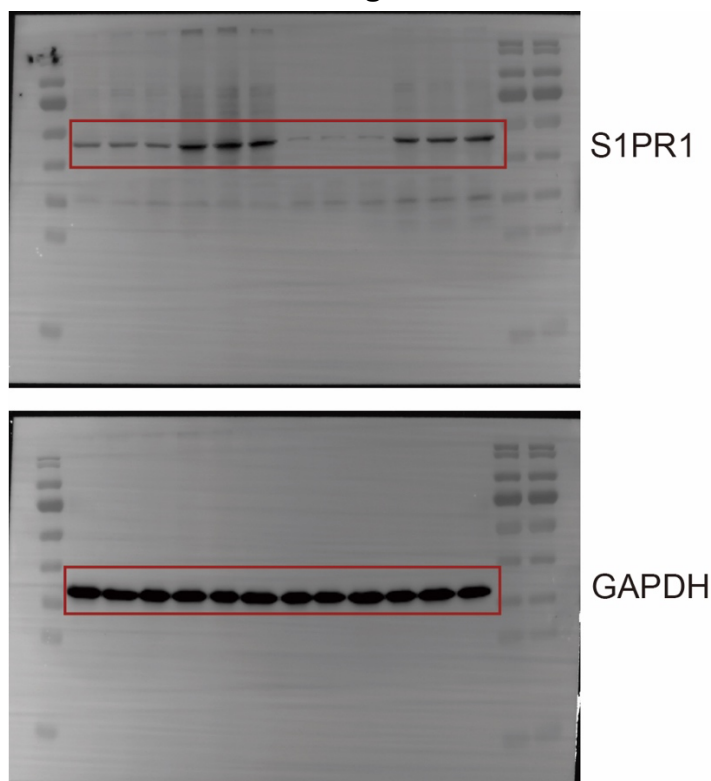

Supplement: Figure 4—source data 1. [file elife-99862-fig4-data1.pdf]

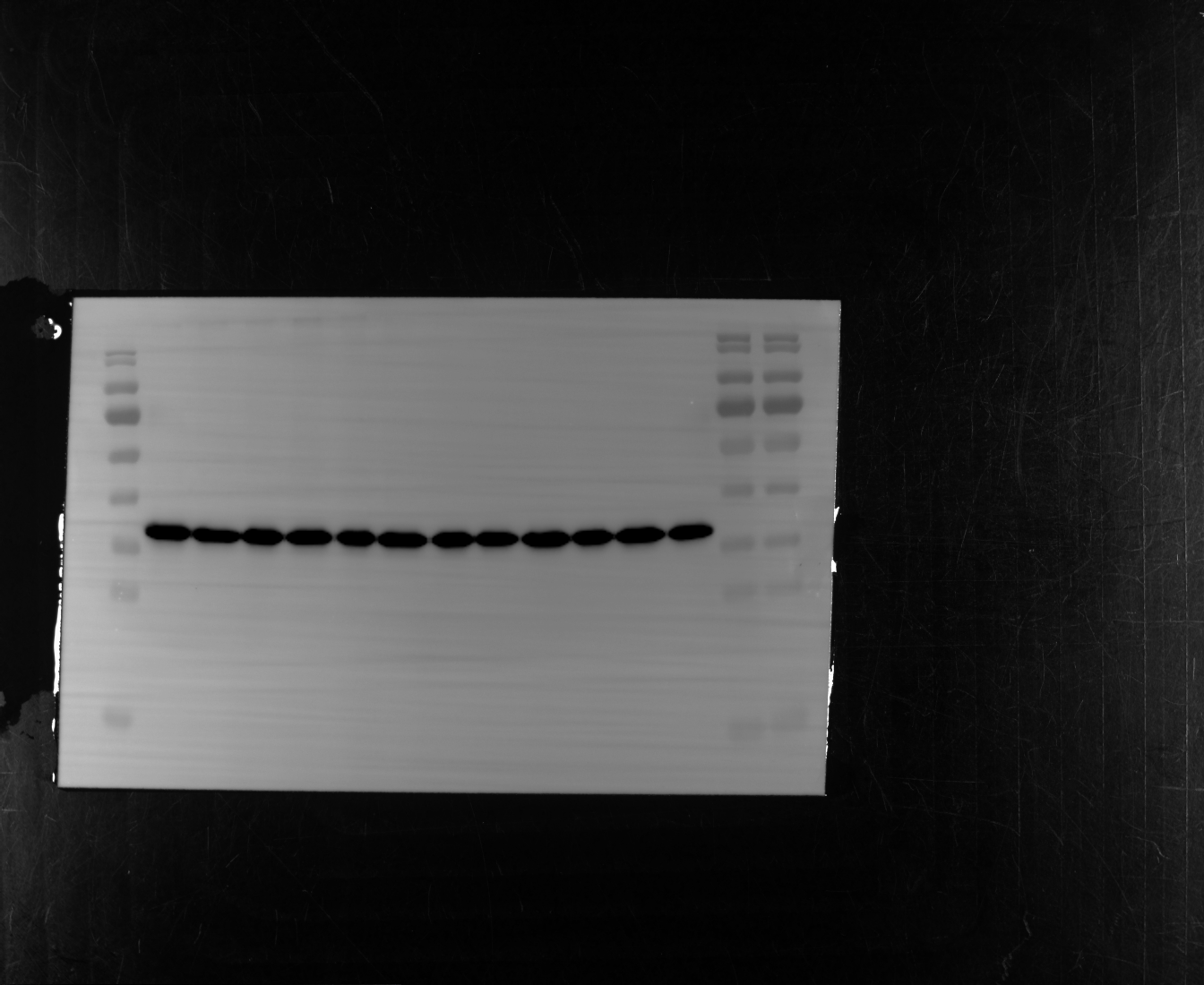

Supplement: Figure 4—source data 2. [file elife-99862-fig4-data2.zip › Figure 4-source data 2 /gapdh.Tif]

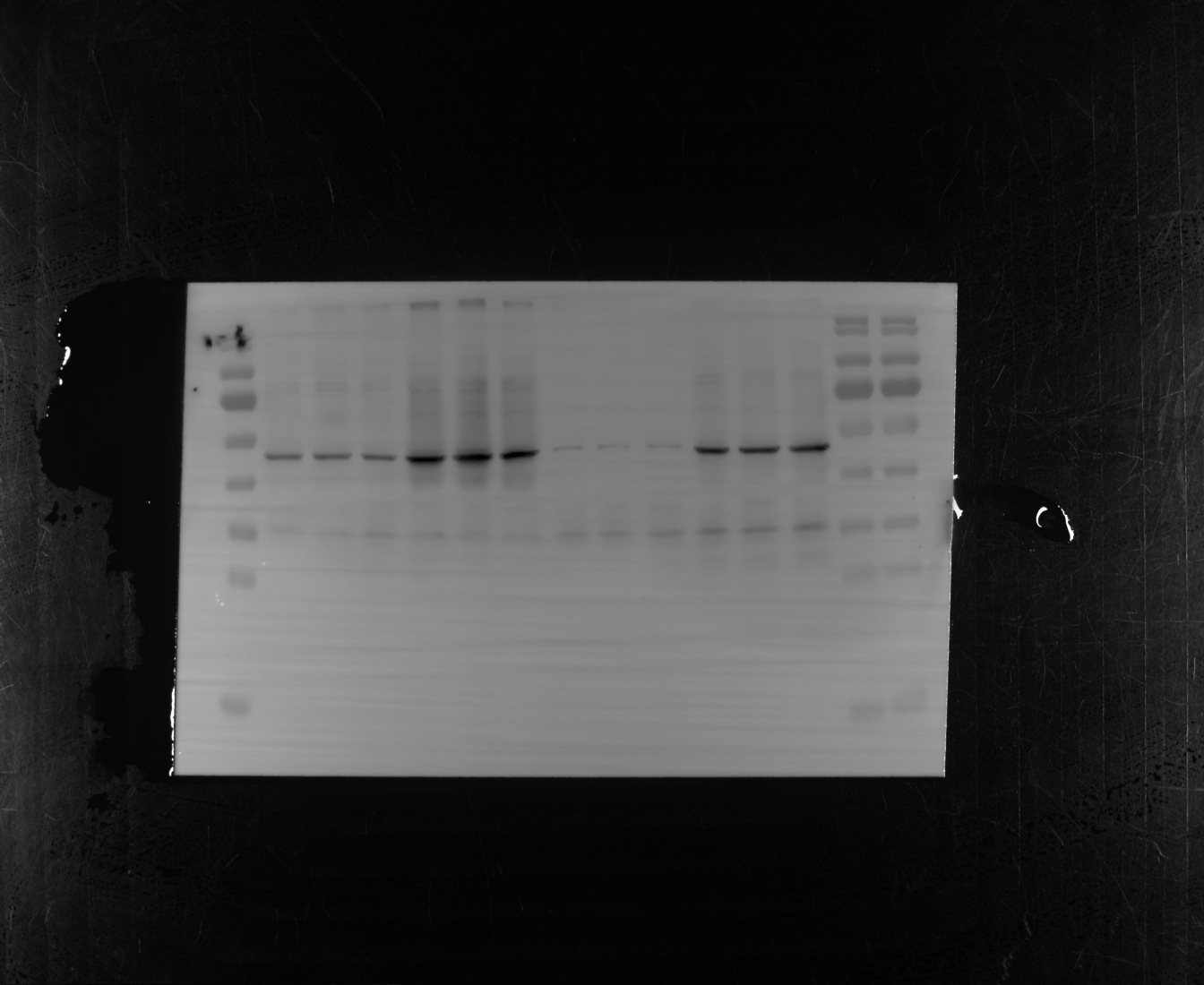

Supplement: Figure 4—source data 2. [file elife-99862-fig4-data2.zip › Figure 4-source data 2 /S1PR1.Tif]

Full unedited blot for Figure 5C

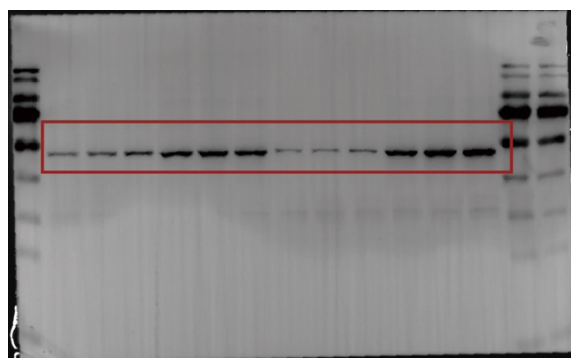

S1PR1

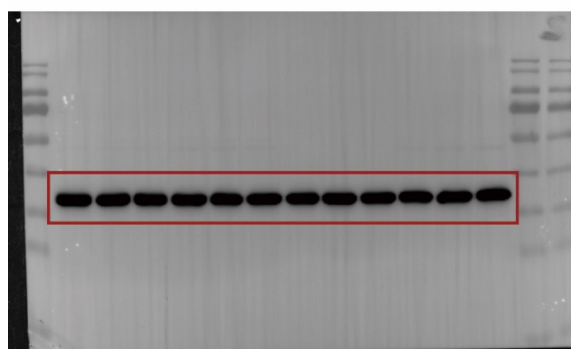

GAPDH

Supplement: Figure 5—source data 1. [file elife-99862-fig5-data1.pdf]

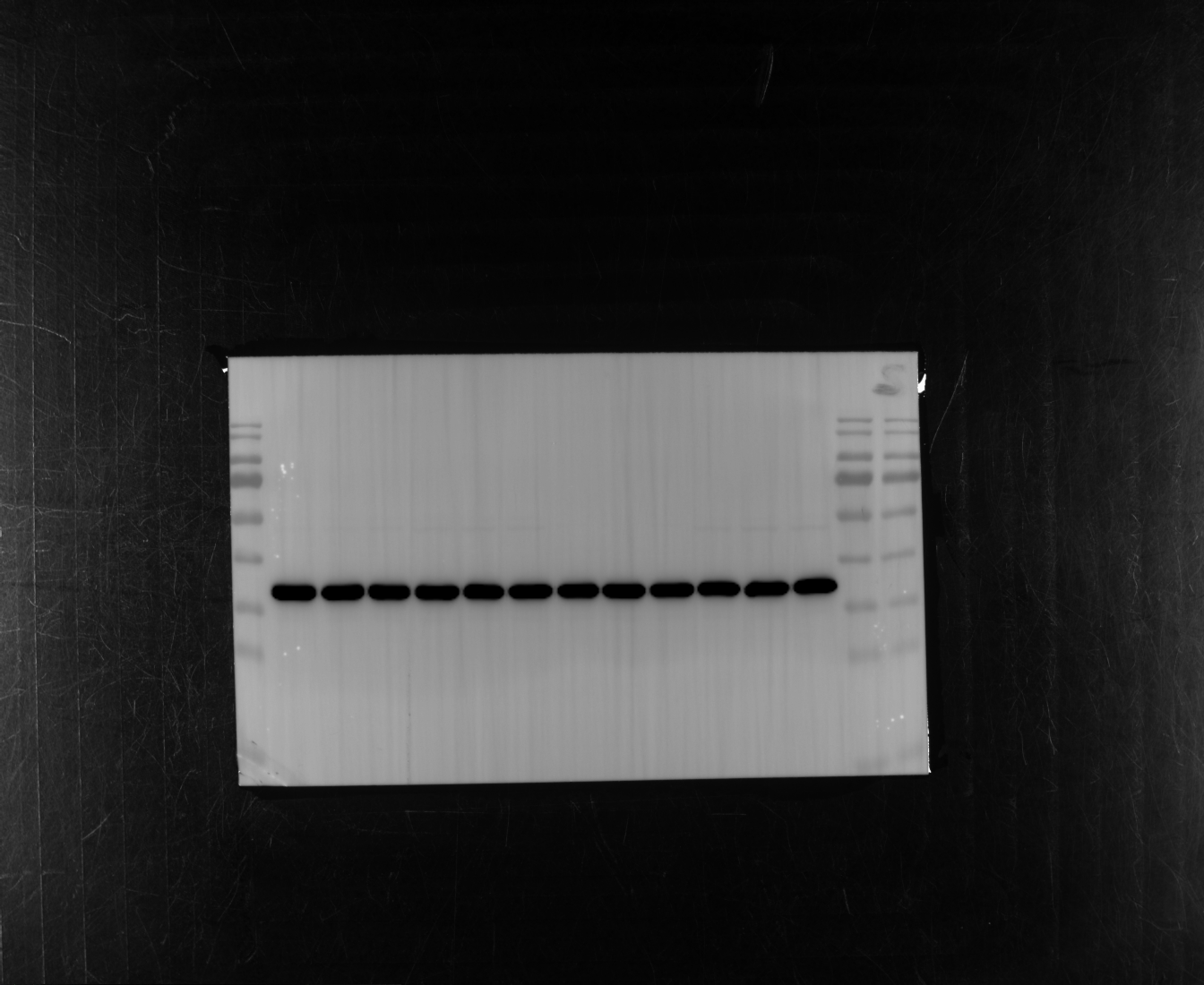

Supplement: Figure 5—source data 2. [file elife-99862-fig5-data2.zip › Figure 5-source data 2/GAPDH.Tif]

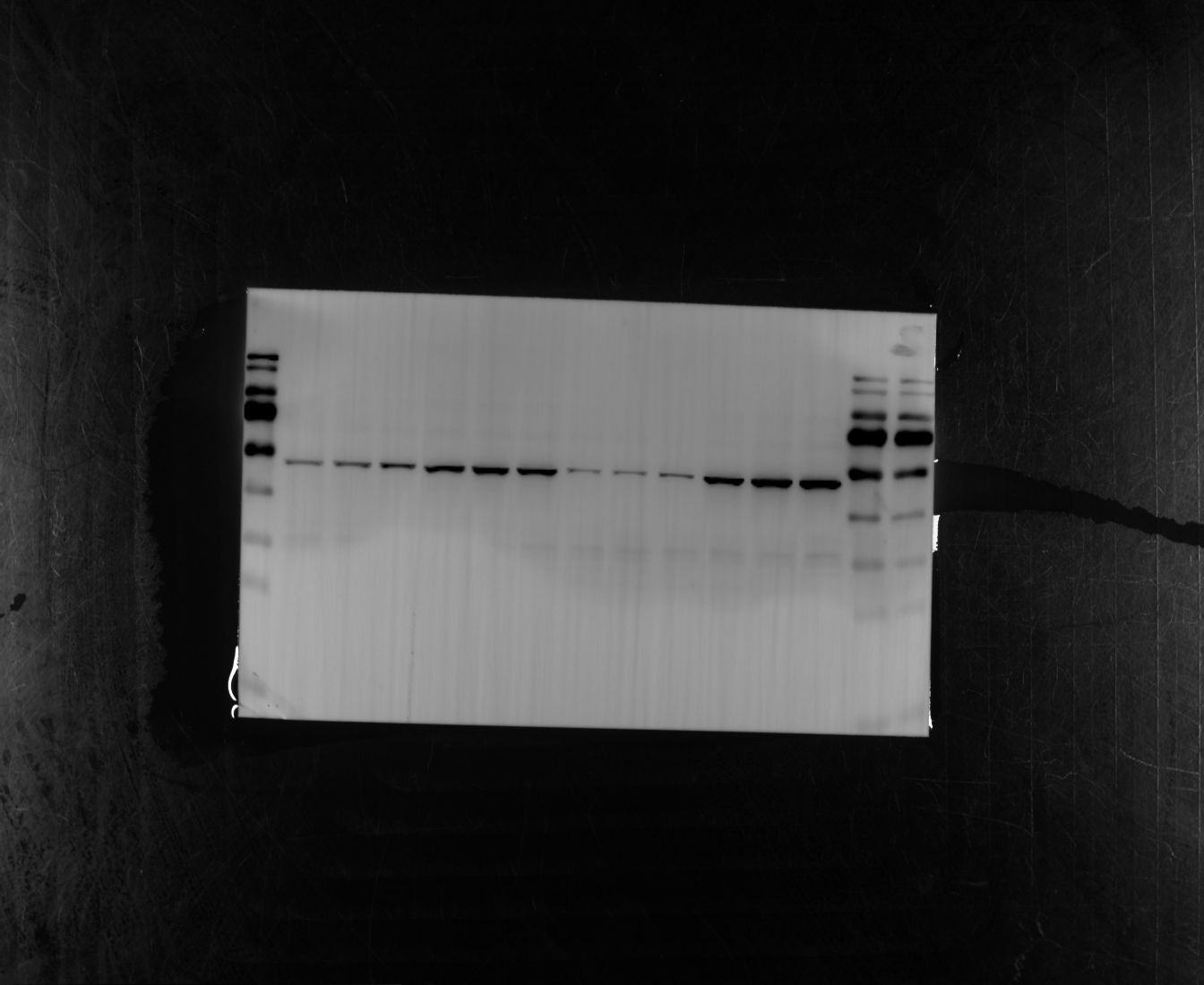

Supplement: Figure 5—source data 2. [file elife-99862-fig5-data2.zip › Figure 5-source data 2/S1PR1.Tif]

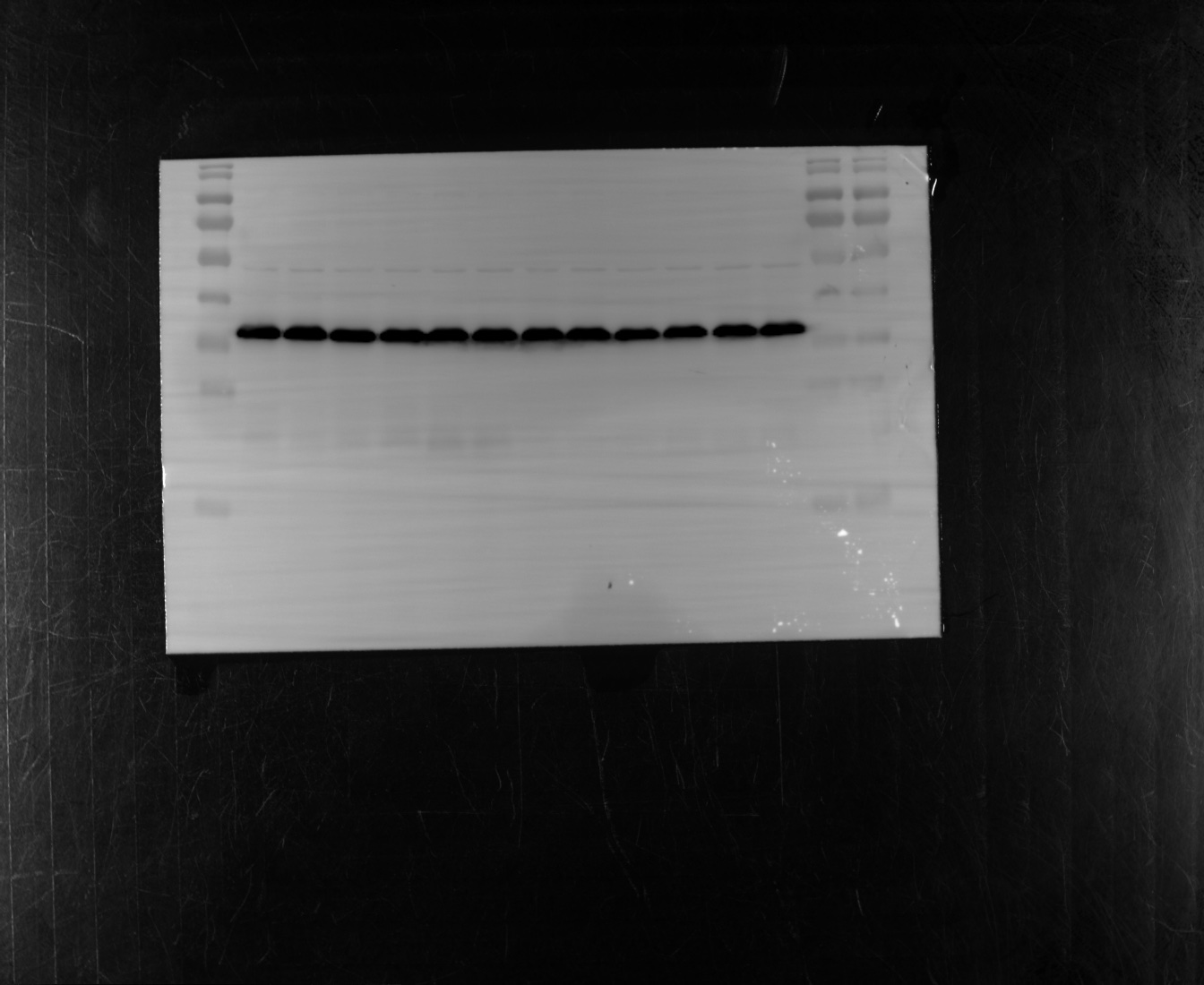

Supplement: Figure 8—source data 2. [file elife-99862-fig8-data2.zip › Figure 8-source data 2 jpgσ¢╛σ░Å/Figure 8I/RAC1/GADPH.jpg]

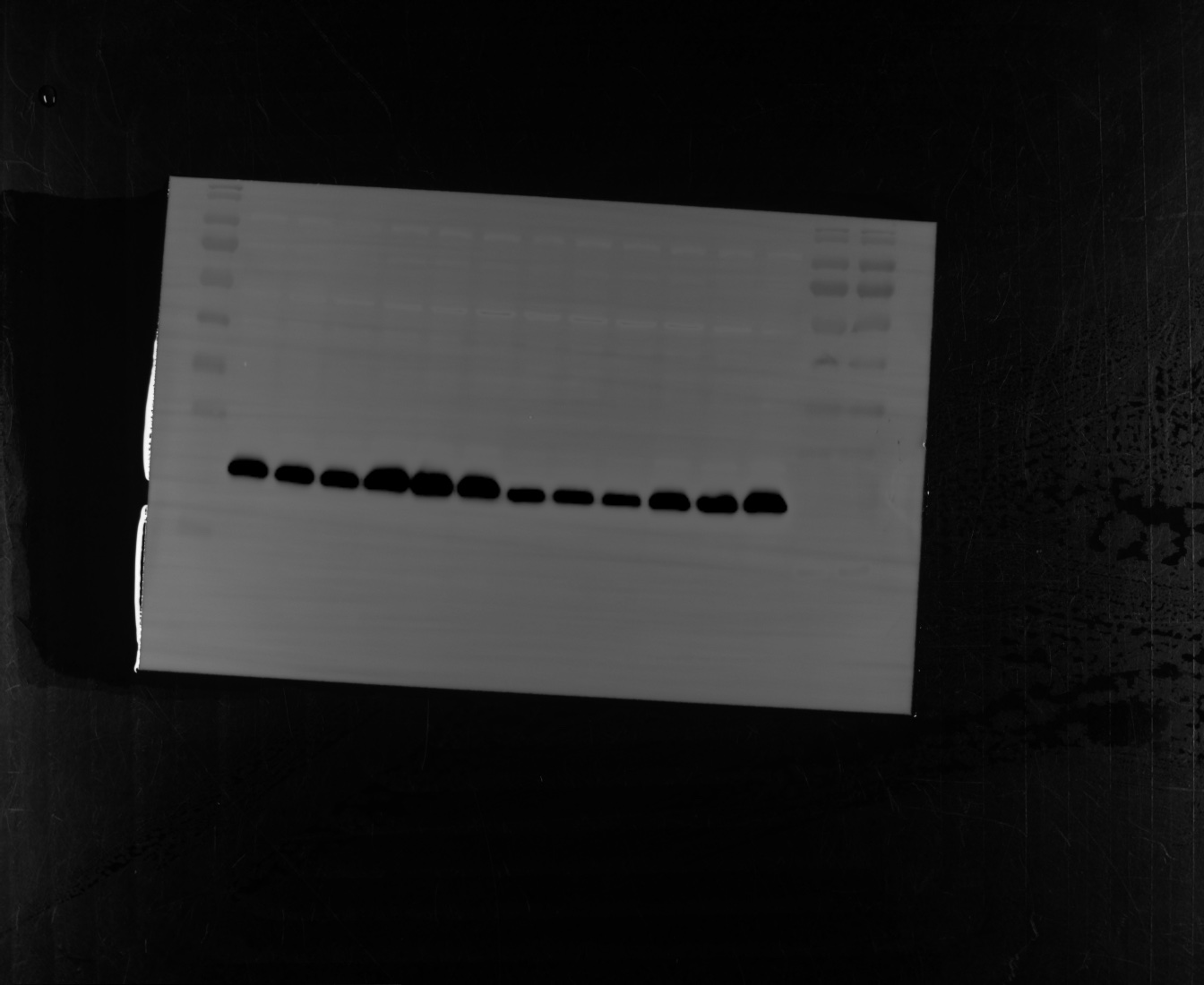

Supplement: Figure 8—source data 2. [file elife-99862-fig8-data2.zip › Figure 8-source data 2 jpgσ¢╛σ░Å/Figure 8I/RAC1/rac1.jpg]

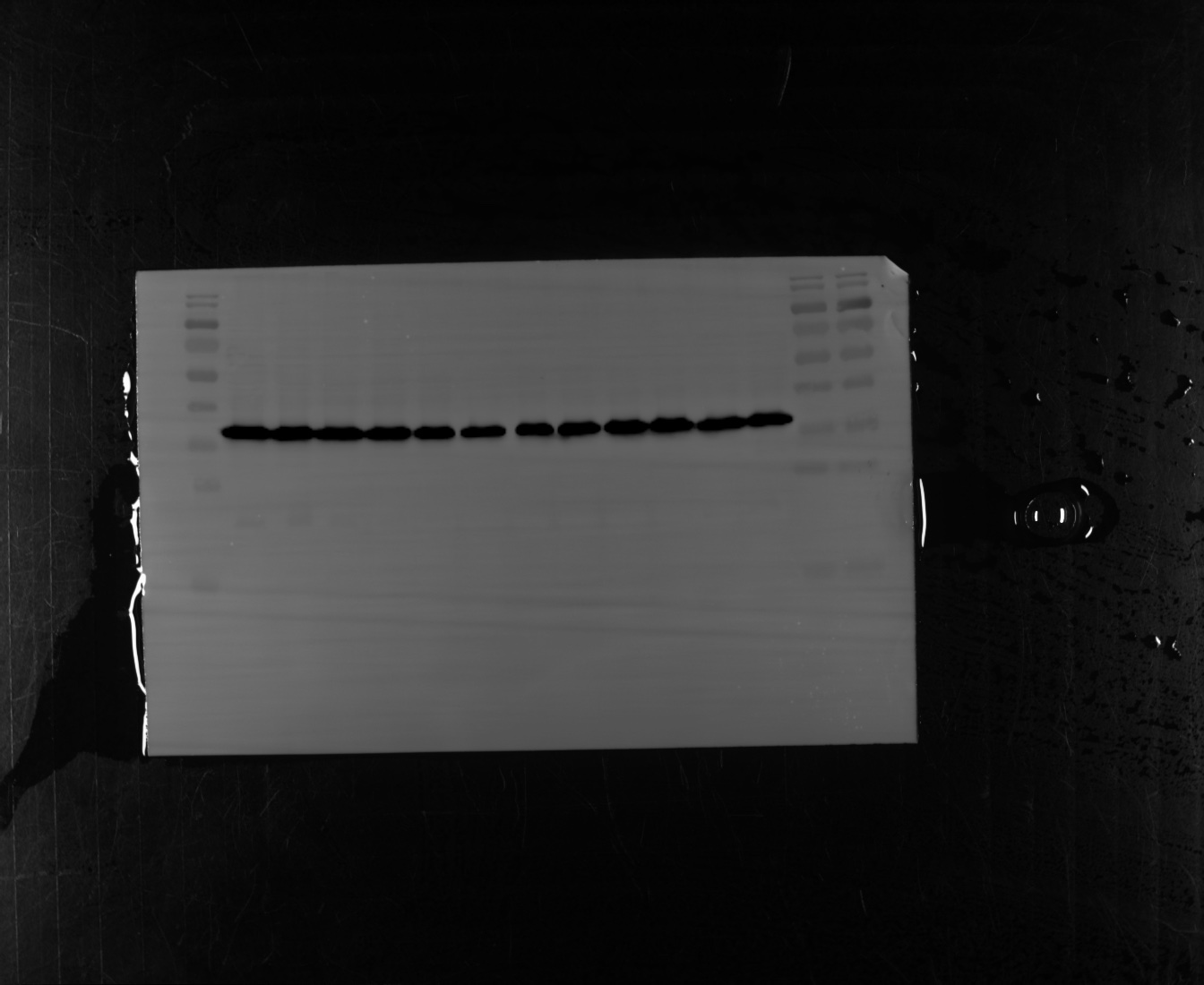

Supplement: Figure 8—source data 2. [file elife-99862-fig8-data2.zip › Figure 8-source data 2 jpgσ¢╛σ░Å/Figure 8I/CDC42/GADPH.jpg]

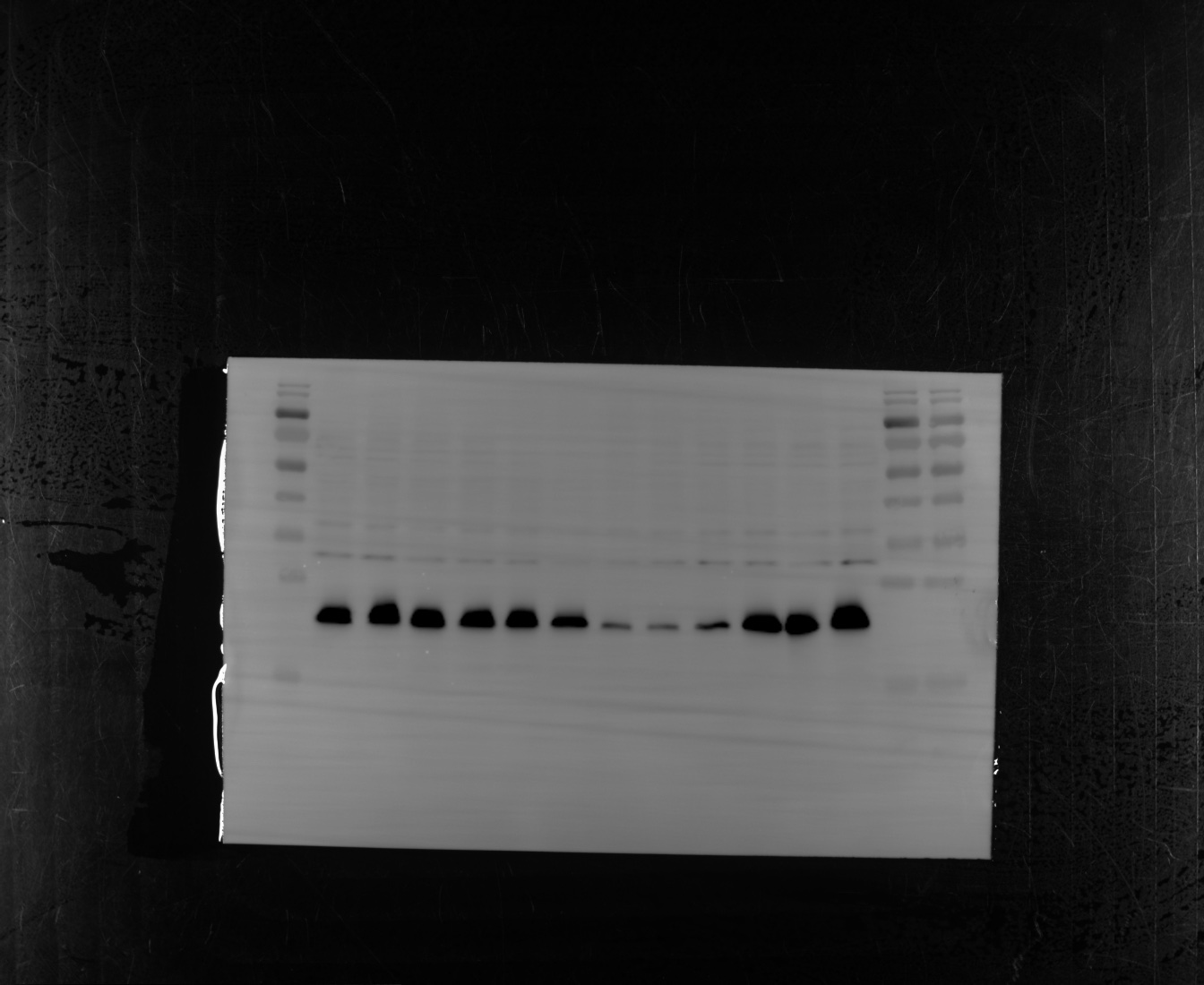

Supplement: Figure 8—source data 2. [file elife-99862-fig8-data2.zip › Figure 8-source data 2 jpgσ¢╛σ░Å/Figure 8I/CDC42/cdc42.jpg]

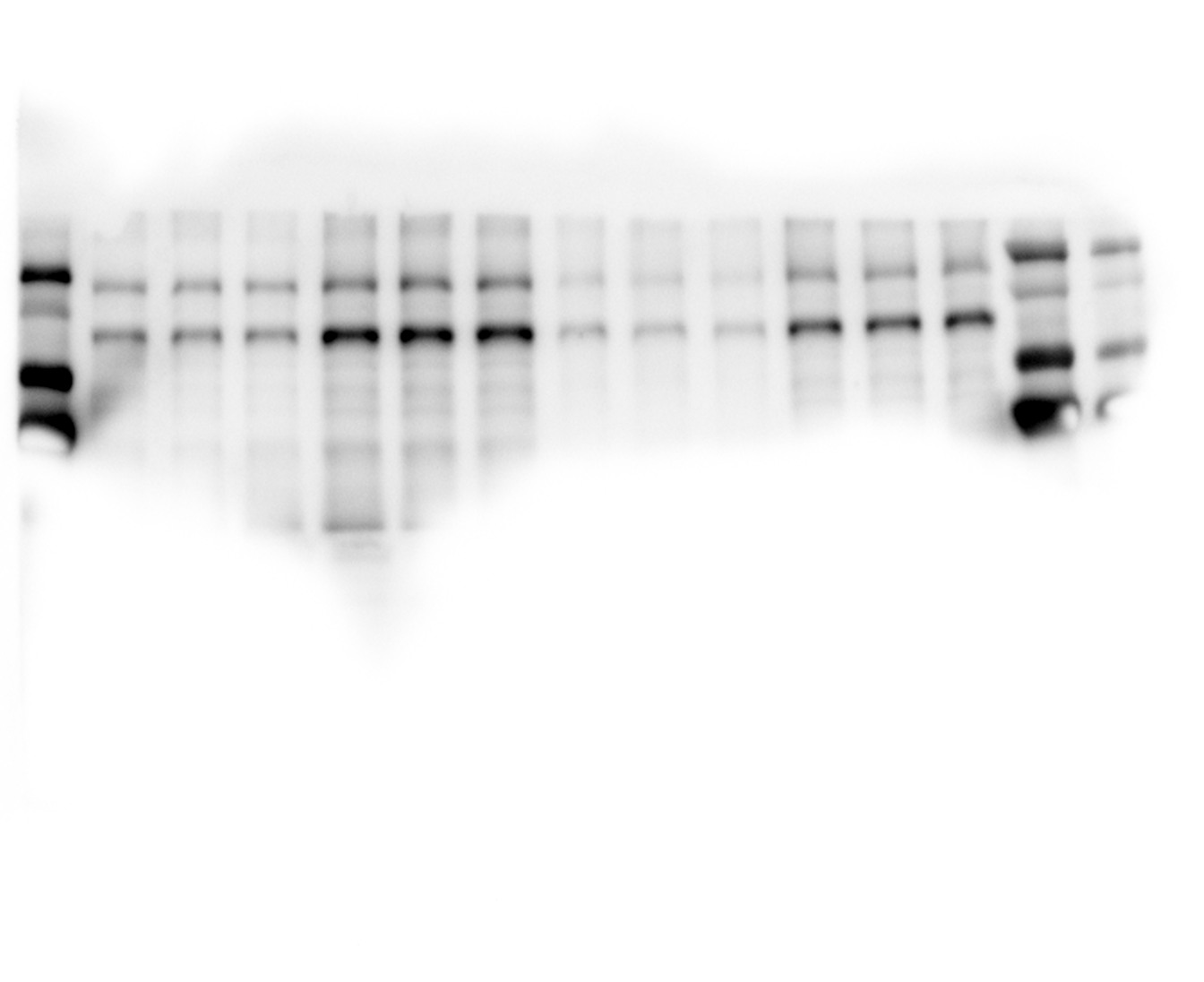

Supplement: Figure 8—source data 2. [file elife-99862-fig8-data2.zip › Figure 8-source data 2 jpgσ¢╛σ░Å/Figure 8I/ITGA2/itga2.Tif]

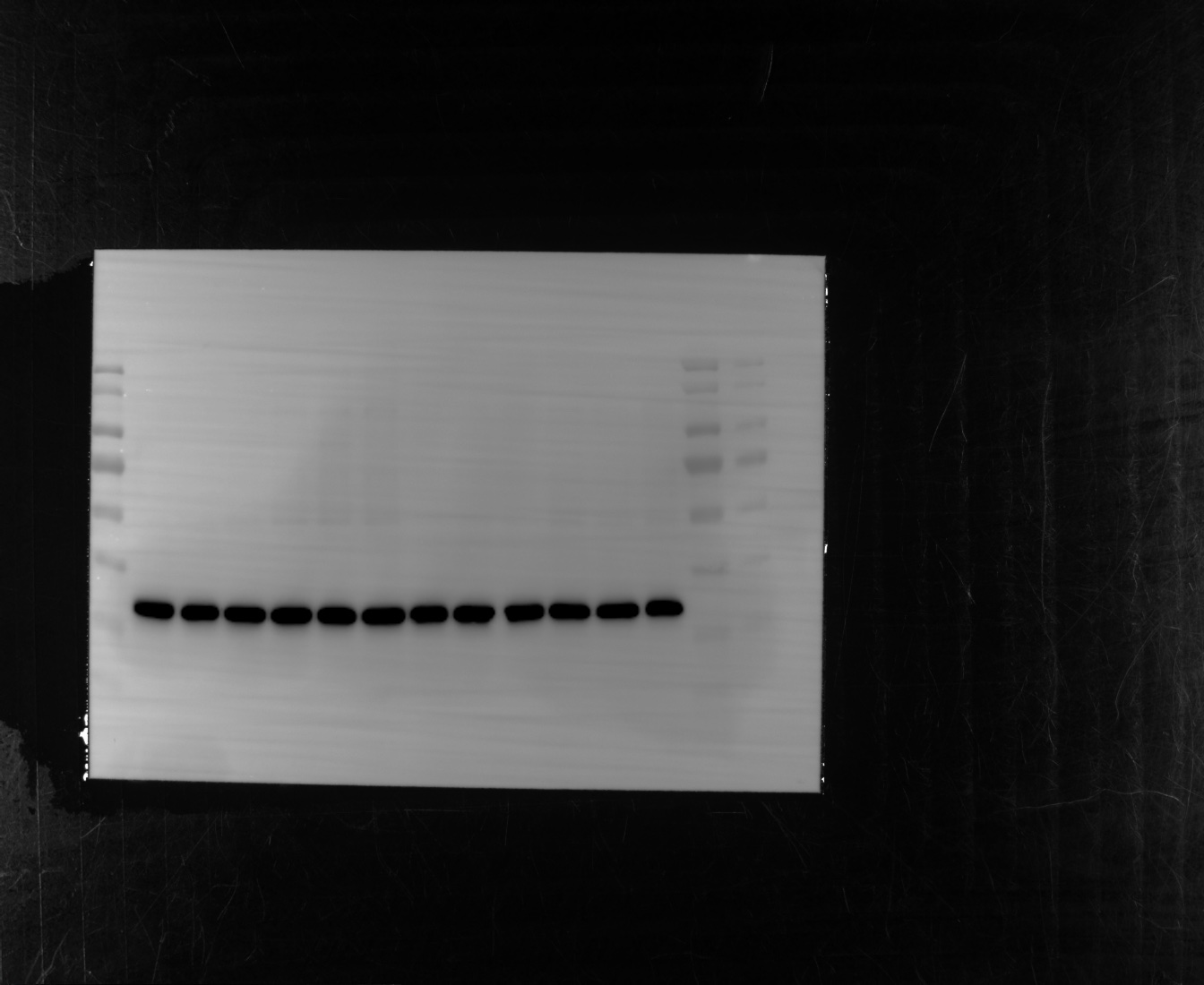

Supplement: Figure 8—source data 2. [file elife-99862-fig8-data2.zip › Figure 8-source data 2 jpgσ¢╛σ░Å/Figure 8I/ITGA2/gapdh.jpg]

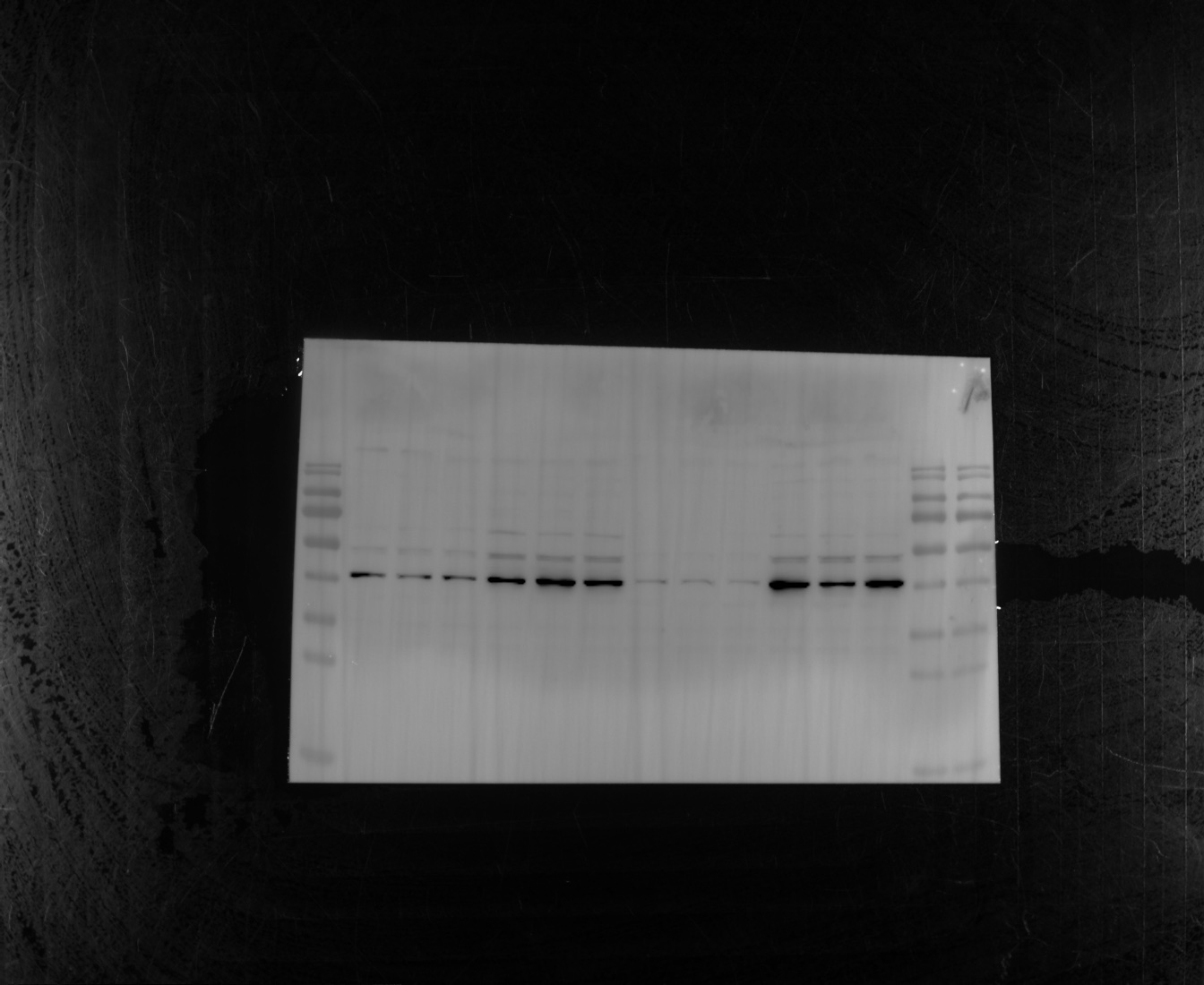

Supplement: Figure 8—source data 2. [file elife-99862-fig8-data2.zip › Figure 8-source data 2 jpgσ¢╛σ░Å/Figure 8I/ARP2/arp2.jpg]

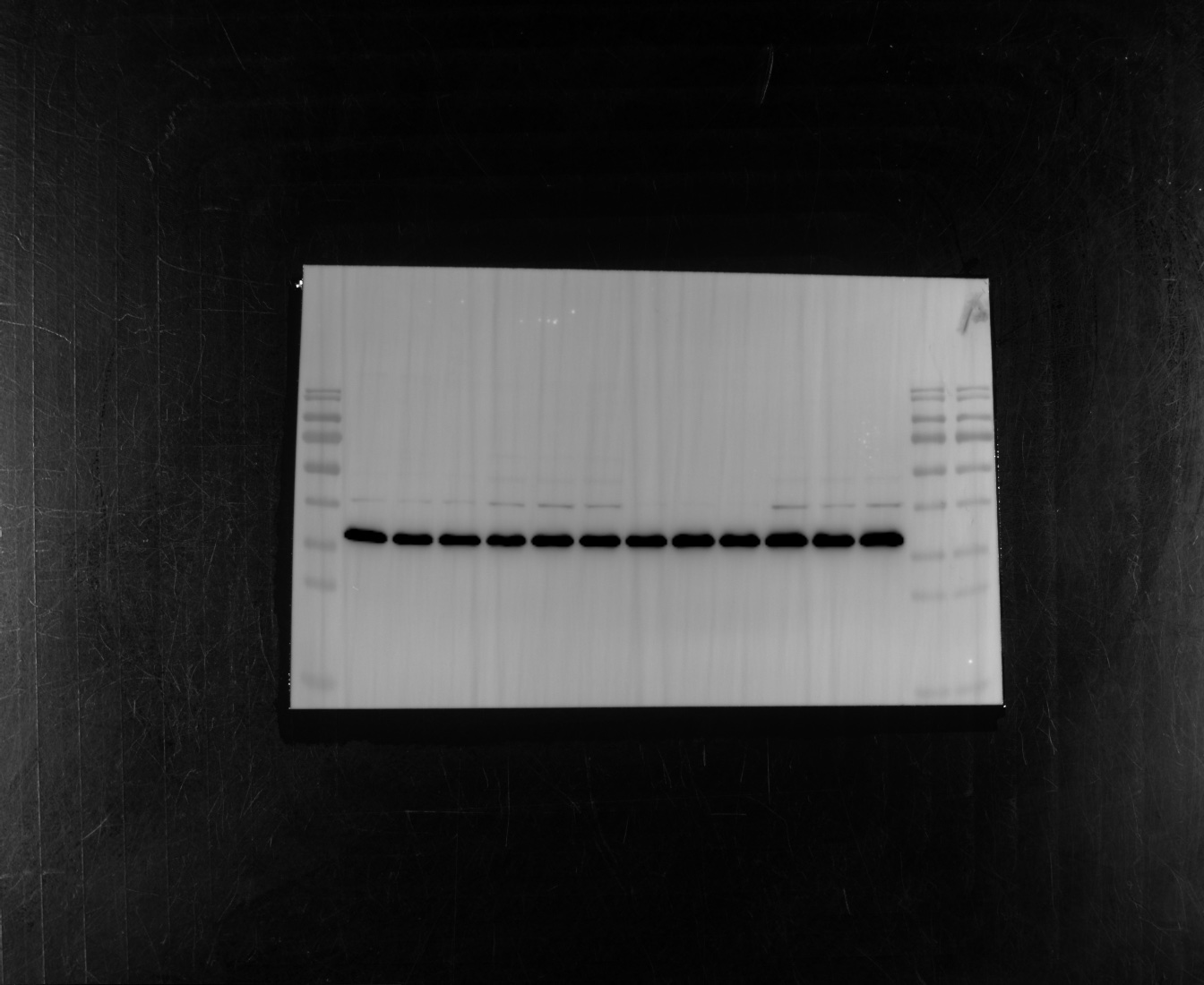

Supplement: Figure 8—source data 2. [file elife-99862-fig8-data2.zip › Figure 8-source data 2 jpgσ¢╛σ░Å/Figure 8I/ARP2/GAPDH.jpg]

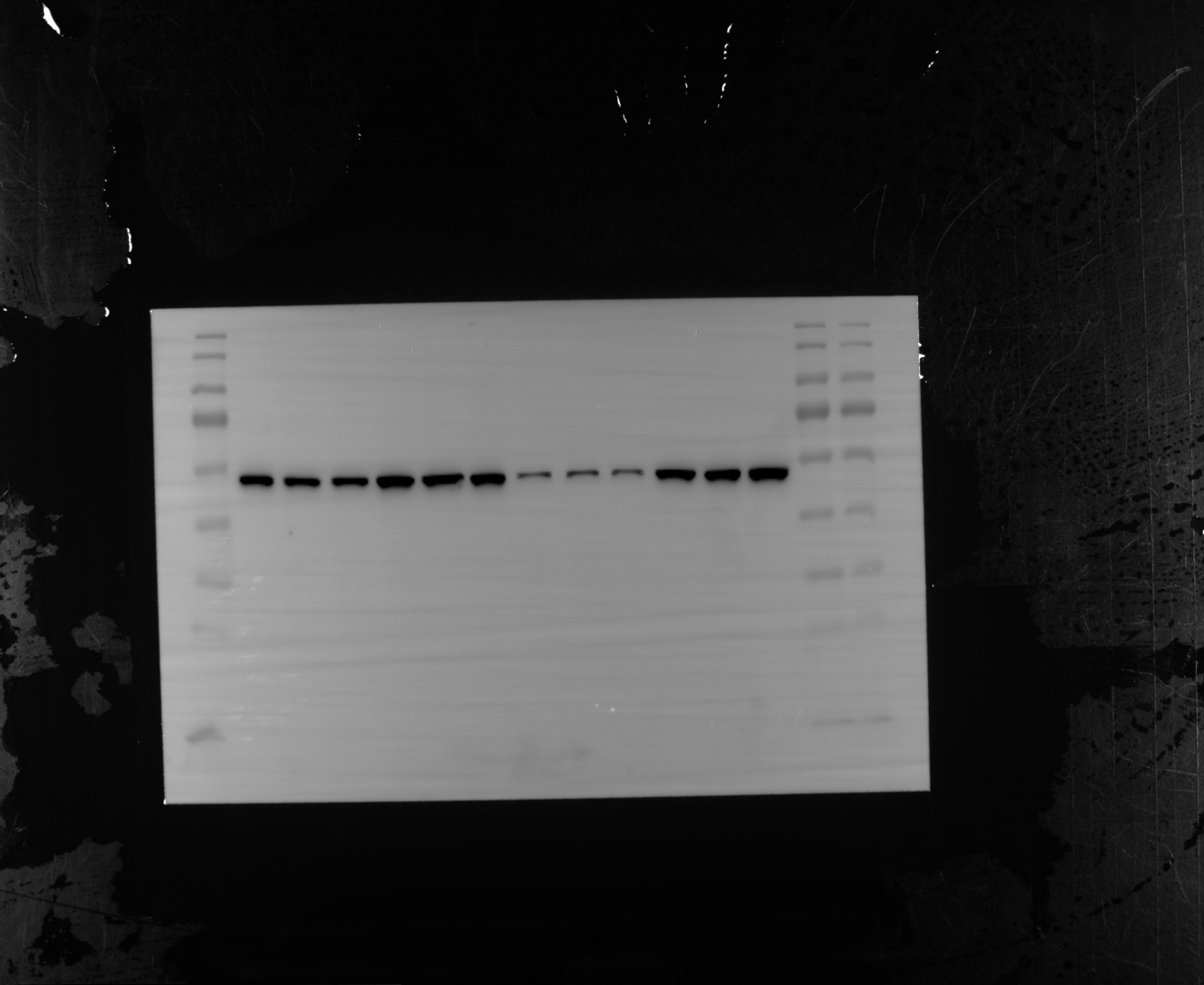

Supplement: Figure 8—source data 2. [file elife-99862-fig8-data2.zip › Figure 8-source data 2 jpgσ¢╛σ░Å/Figure 8I/ARP3/arp3.jpg]

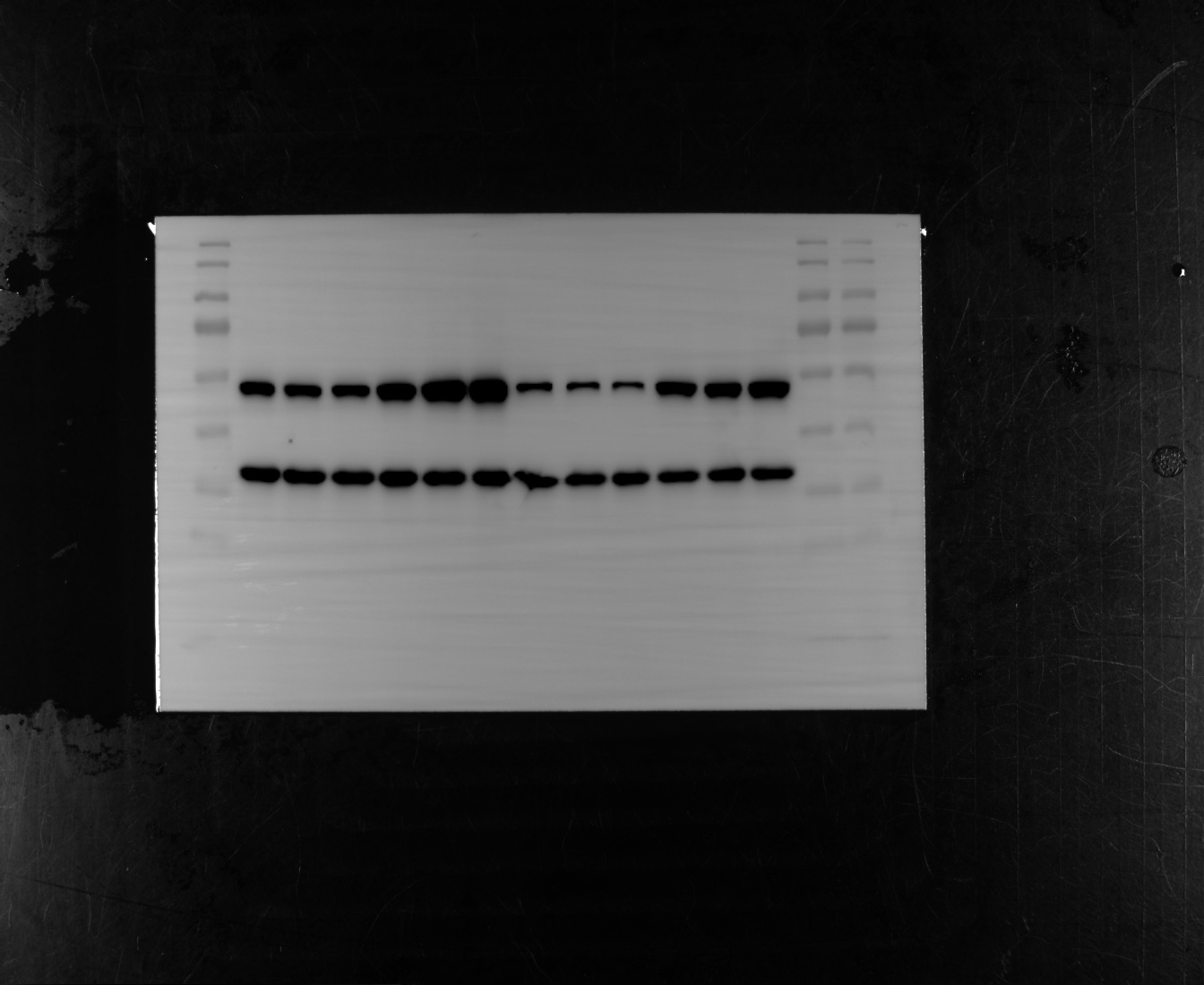

Supplement: Figure 8—source data 2. [file elife-99862-fig8-data2.zip › Figure 8-source data 2 jpgσ¢╛σ░Å/Figure 8I/ARP3/gapdh.jpg]

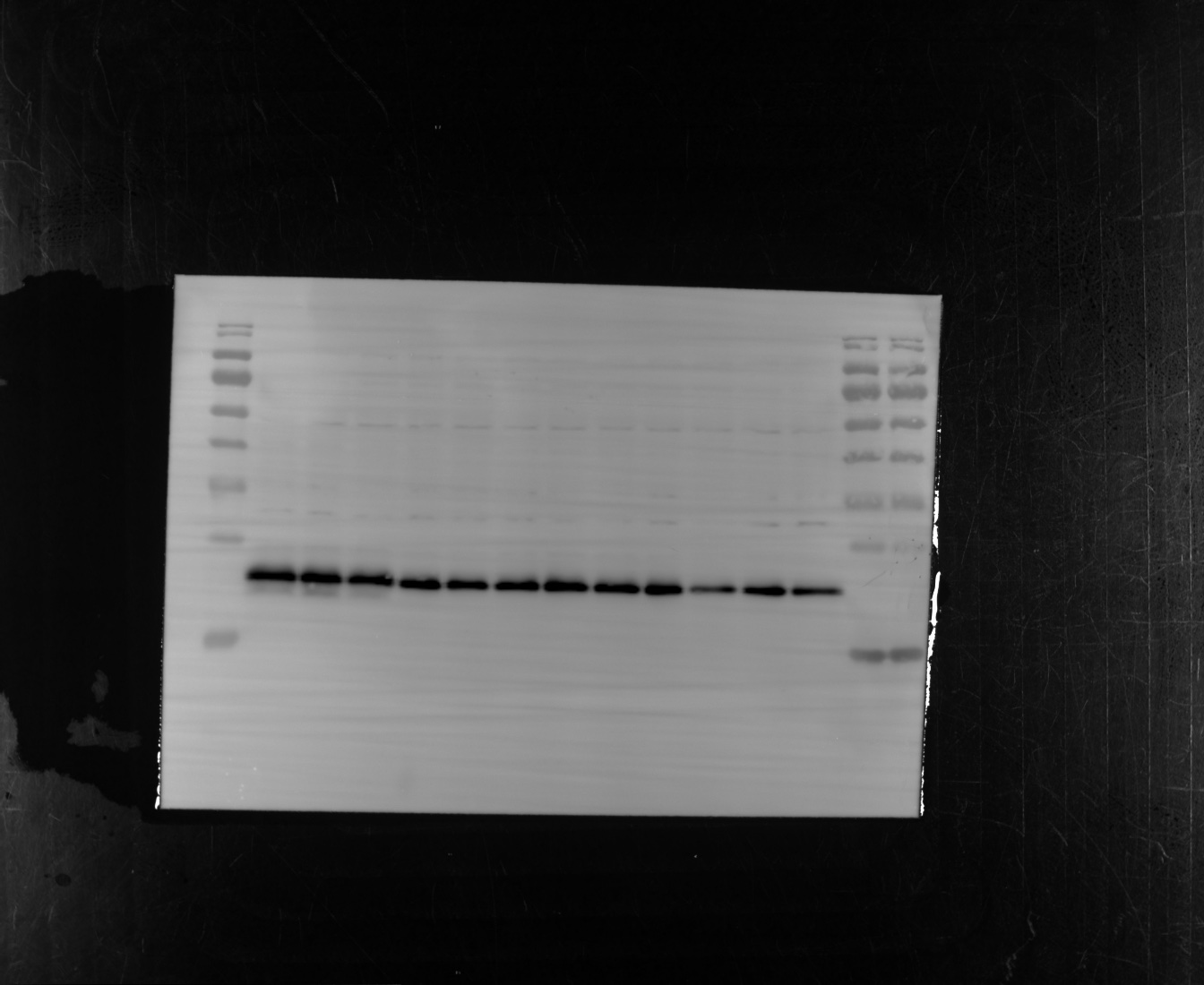

Supplement: Figure 8—source data 2. [file elife-99862-fig8-data2.zip › Figure 8-source data 2 jpgσ¢╛σ░Å/Figure 8G/RAC1/rac1.jpg]

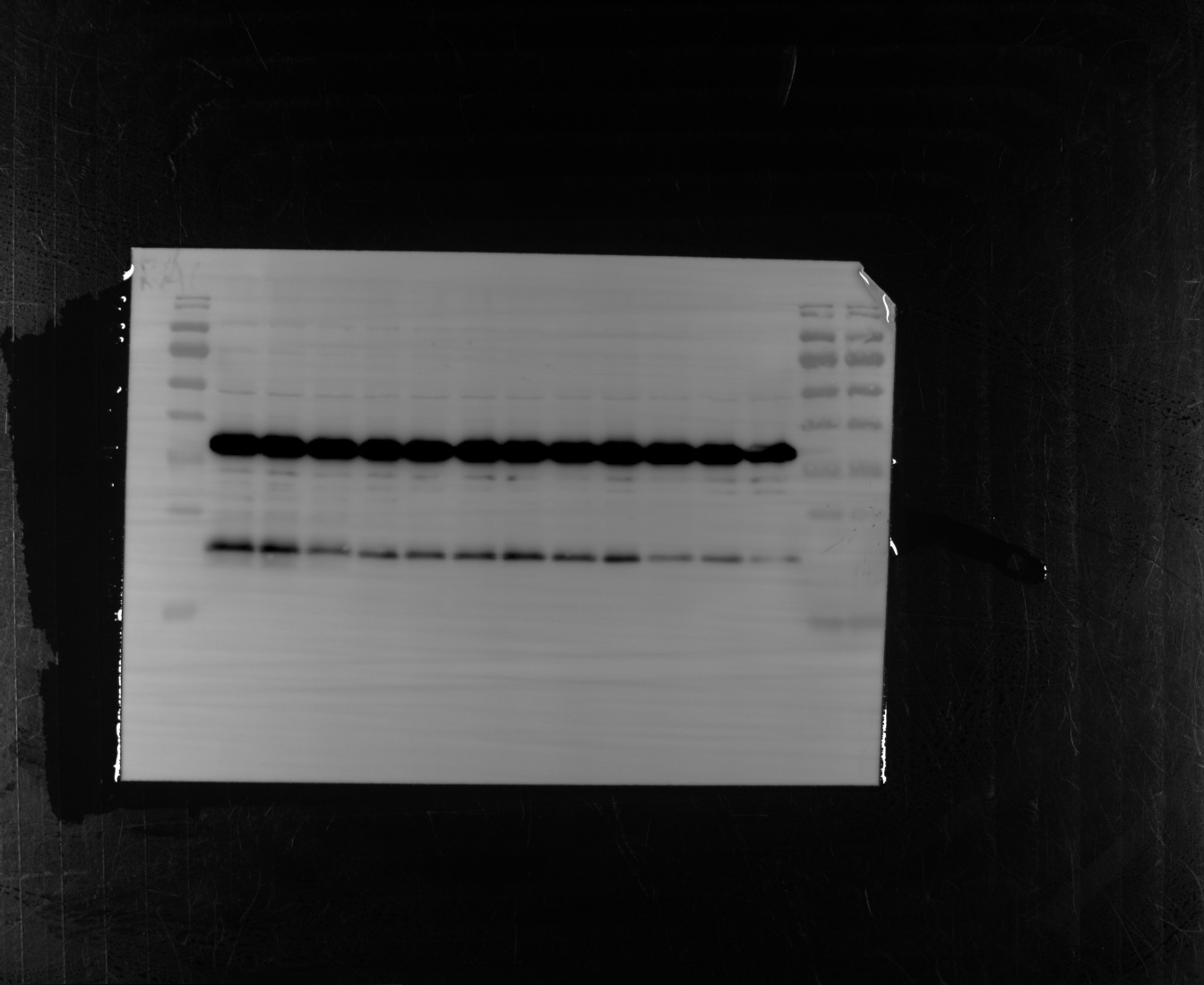

Supplement: Figure 8—source data 2. [file elife-99862-fig8-data2.zip › Figure 8-source data 2 jpgσ¢╛σ░Å/Figure 8G/RAC1/gapdh.jpg]

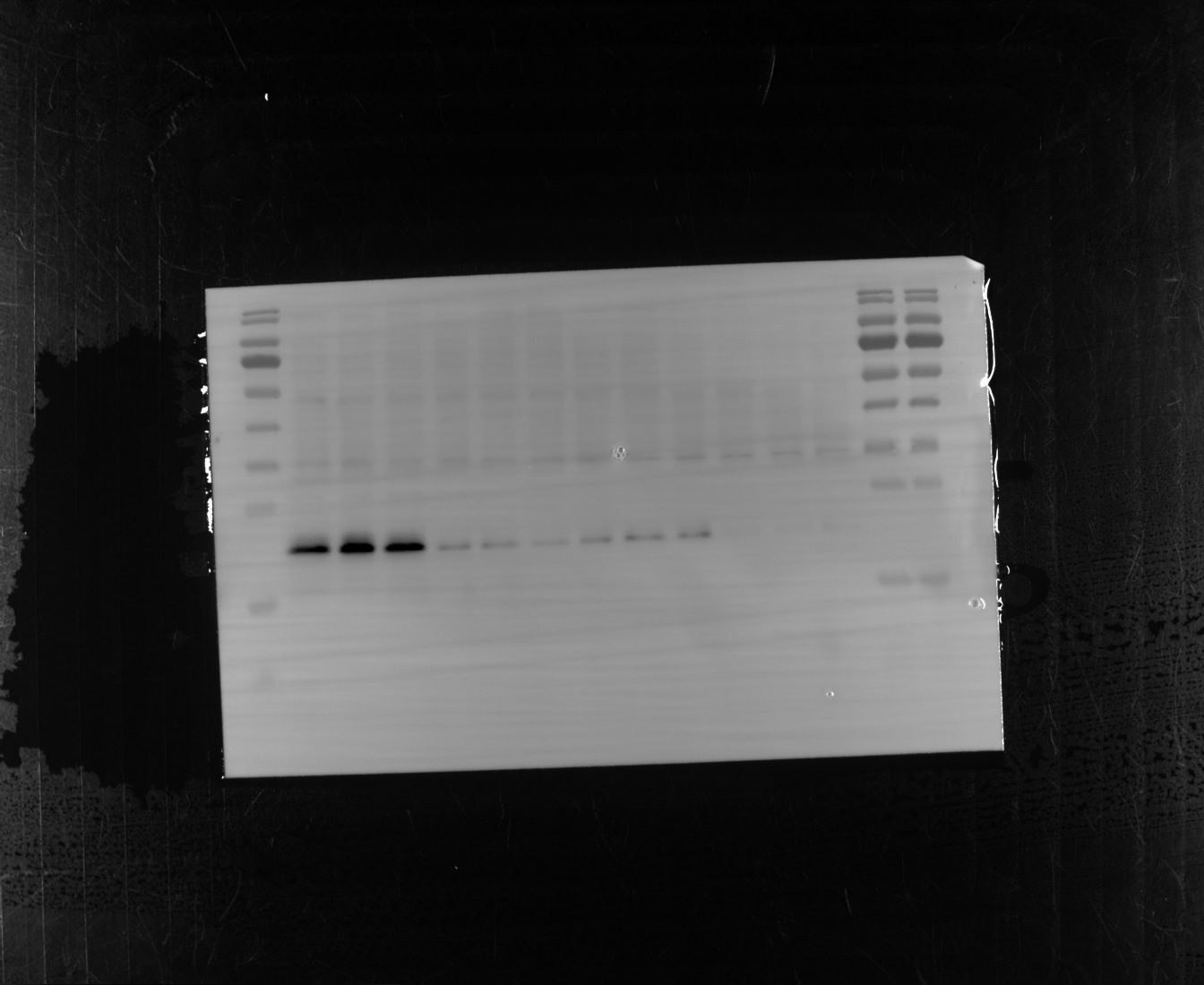

Supplement: Figure 8—source data 2. [file elife-99862-fig8-data2.zip › Figure 8-source data 2 jpgσ¢╛σ░Å/Figure 8G/CDC42/cdc42 2.jpg]

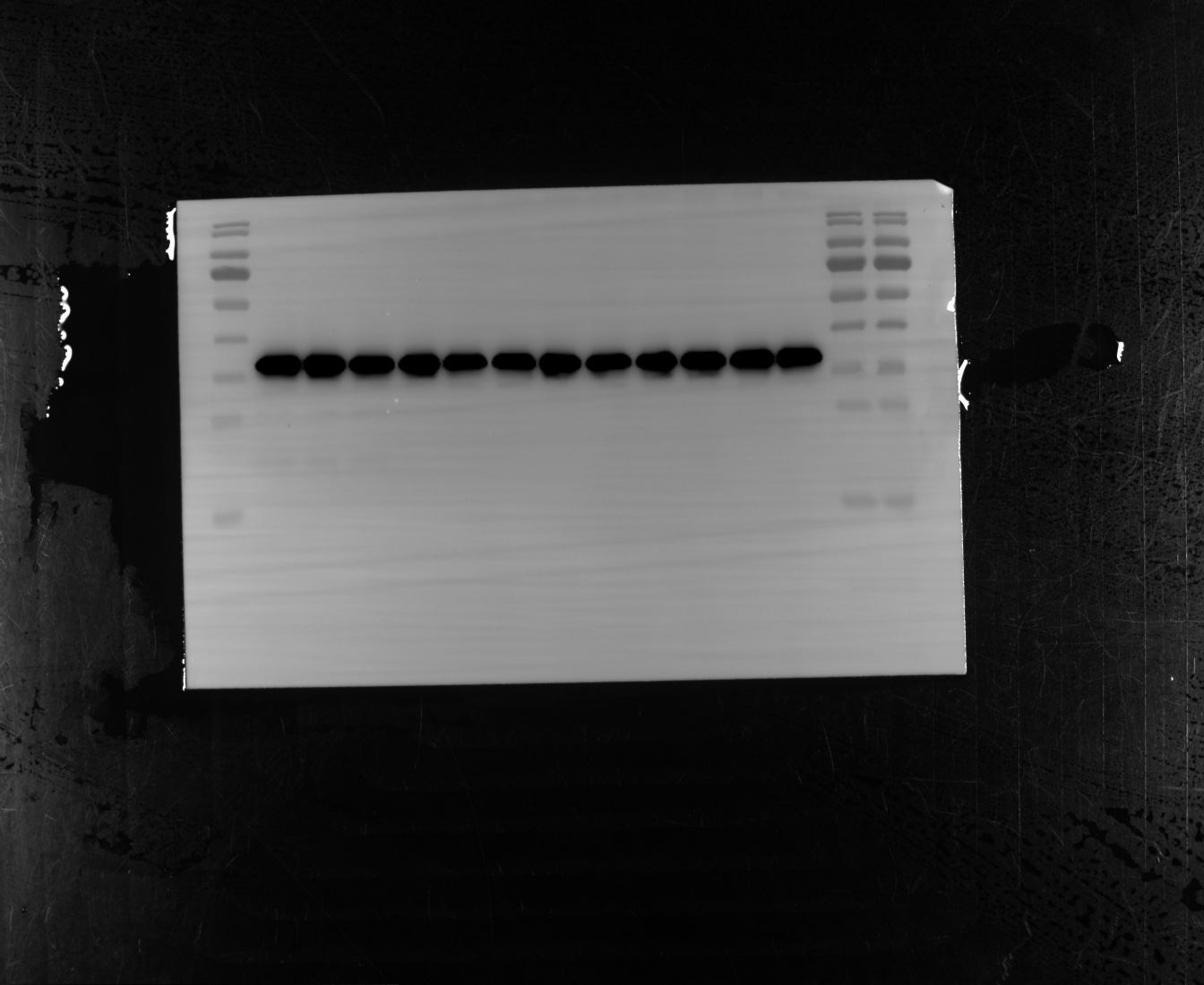

Supplement: Figure 8—source data 2. [file elife-99862-fig8-data2.zip › Figure 8-source data 2 jpgσ¢╛σ░Å/Figure 8G/CDC42/GAPDH 1.jpg]

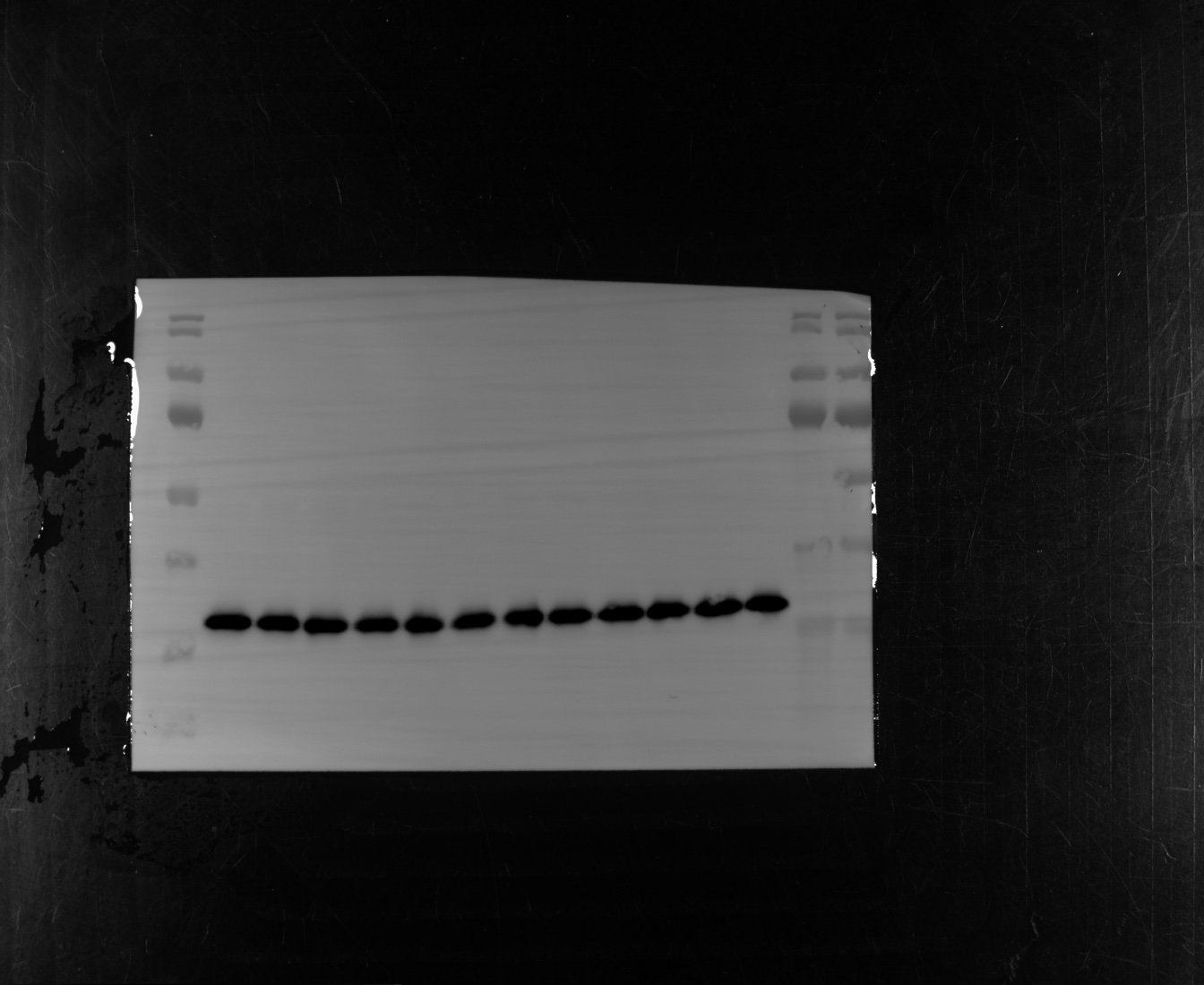

Supplement: Figure 8—source data 2. [file elife-99862-fig8-data2.zip › Figure 8-source data 2 jpgσ¢╛σ░Å/Figure 8G/ITGA2/GAPDH.jpg]

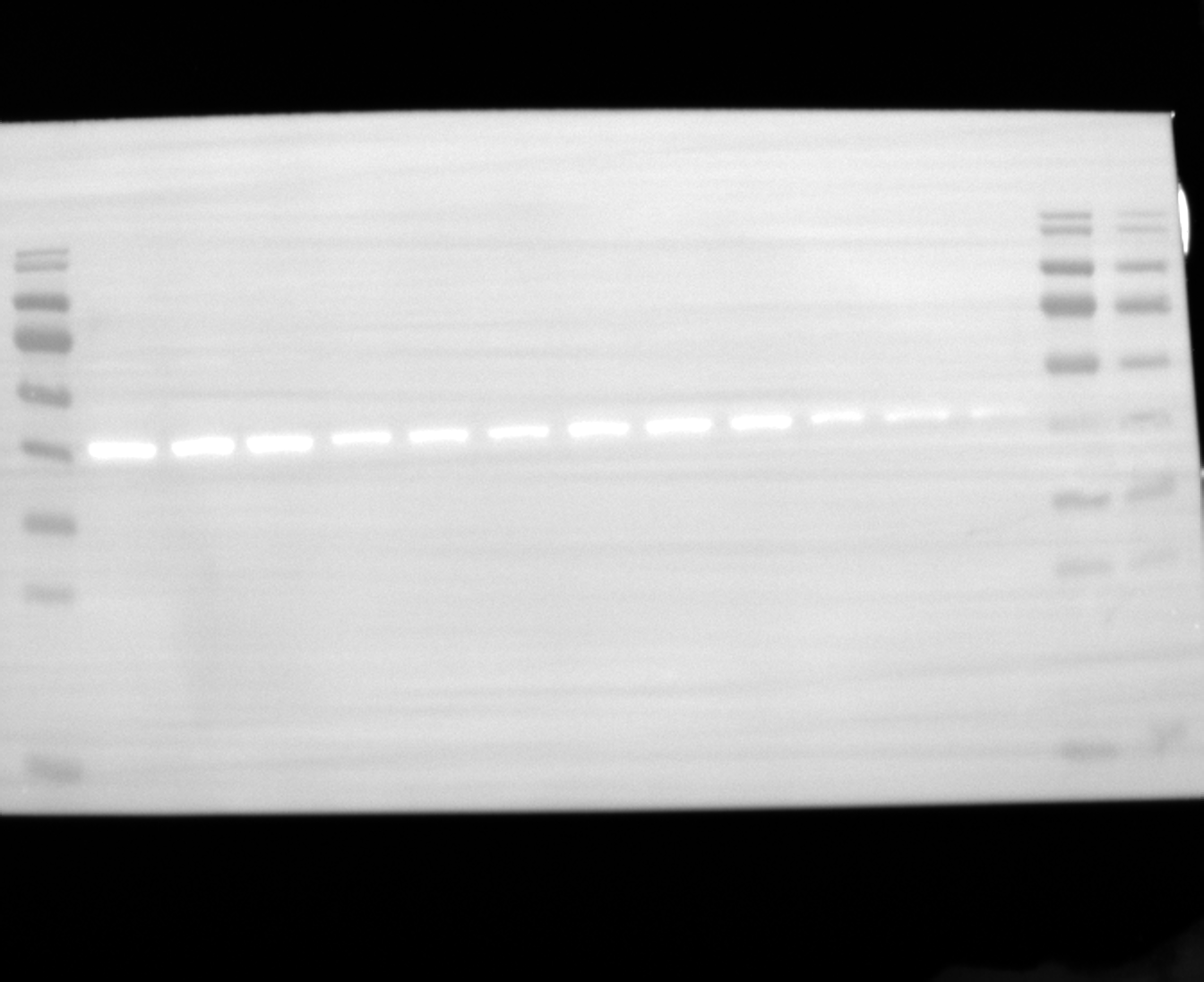

Supplement: Figure 8—source data 2. [file elife-99862-fig8-data2.zip › Figure 8-source data 2 jpgσ¢╛σ░Å/Figure 8G/ARP2/arp2.Tif]

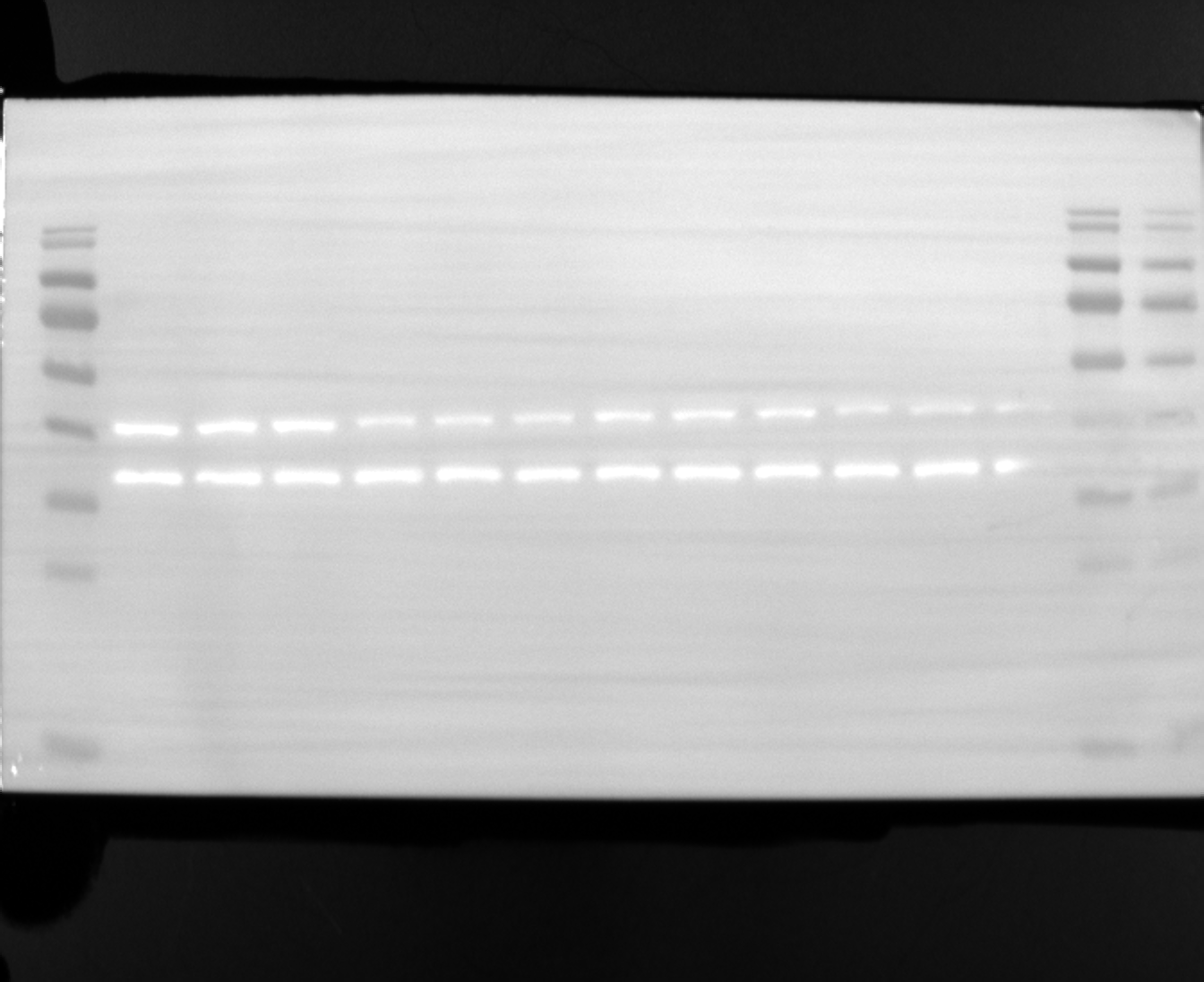

Supplement: Figure 8—source data 2. [file elife-99862-fig8-data2.zip › Figure 8-source data 2 jpgσ¢╛σ░Å/Figure 8G/ARP2/gapdh.Tif]

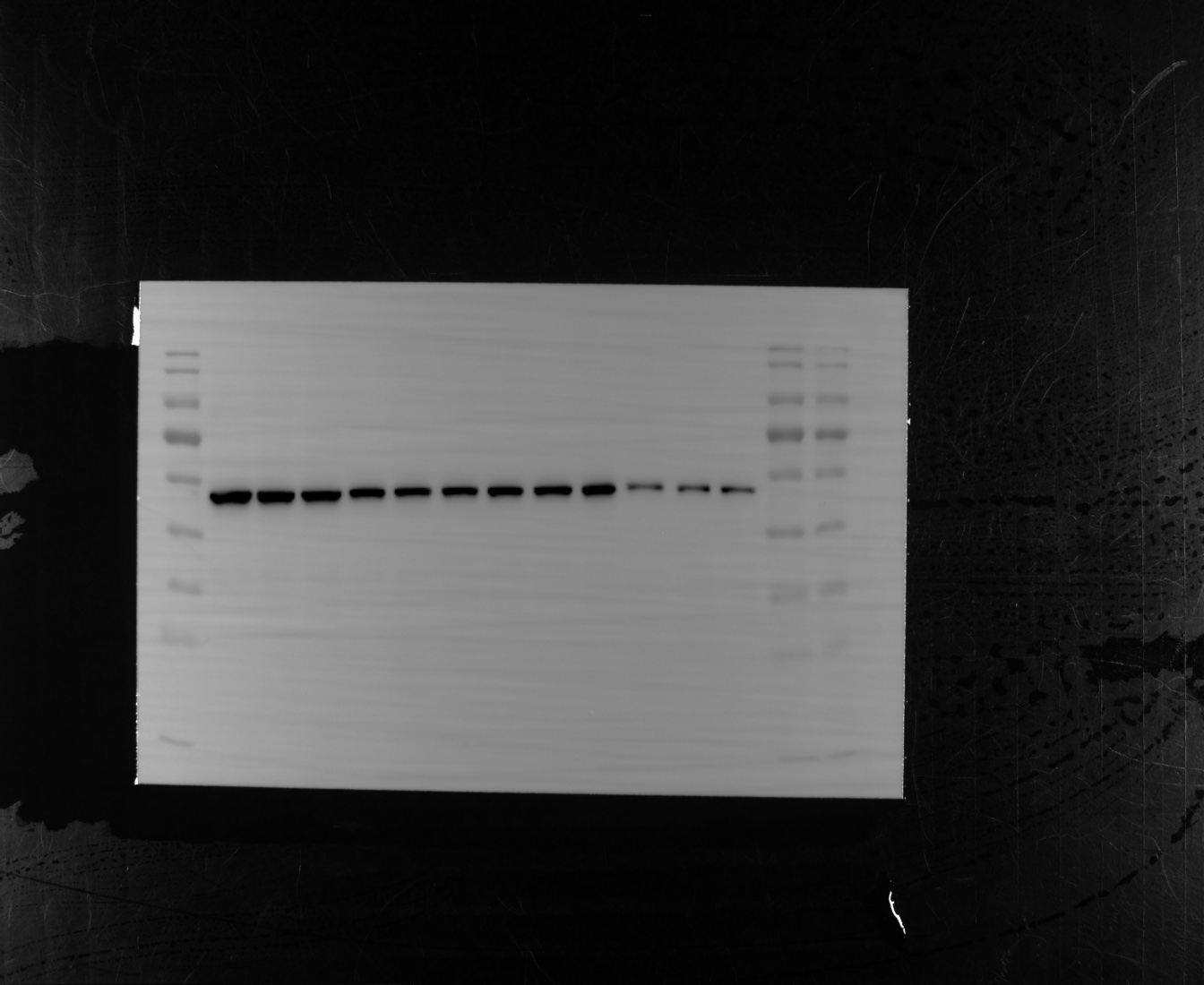

Supplement: Figure 8—source data 2. [file elife-99862-fig8-data2.zip › Figure 8-source data 2 jpgσ¢╛σ░Å/Figure 8G/ARP3/arp3.jpg]

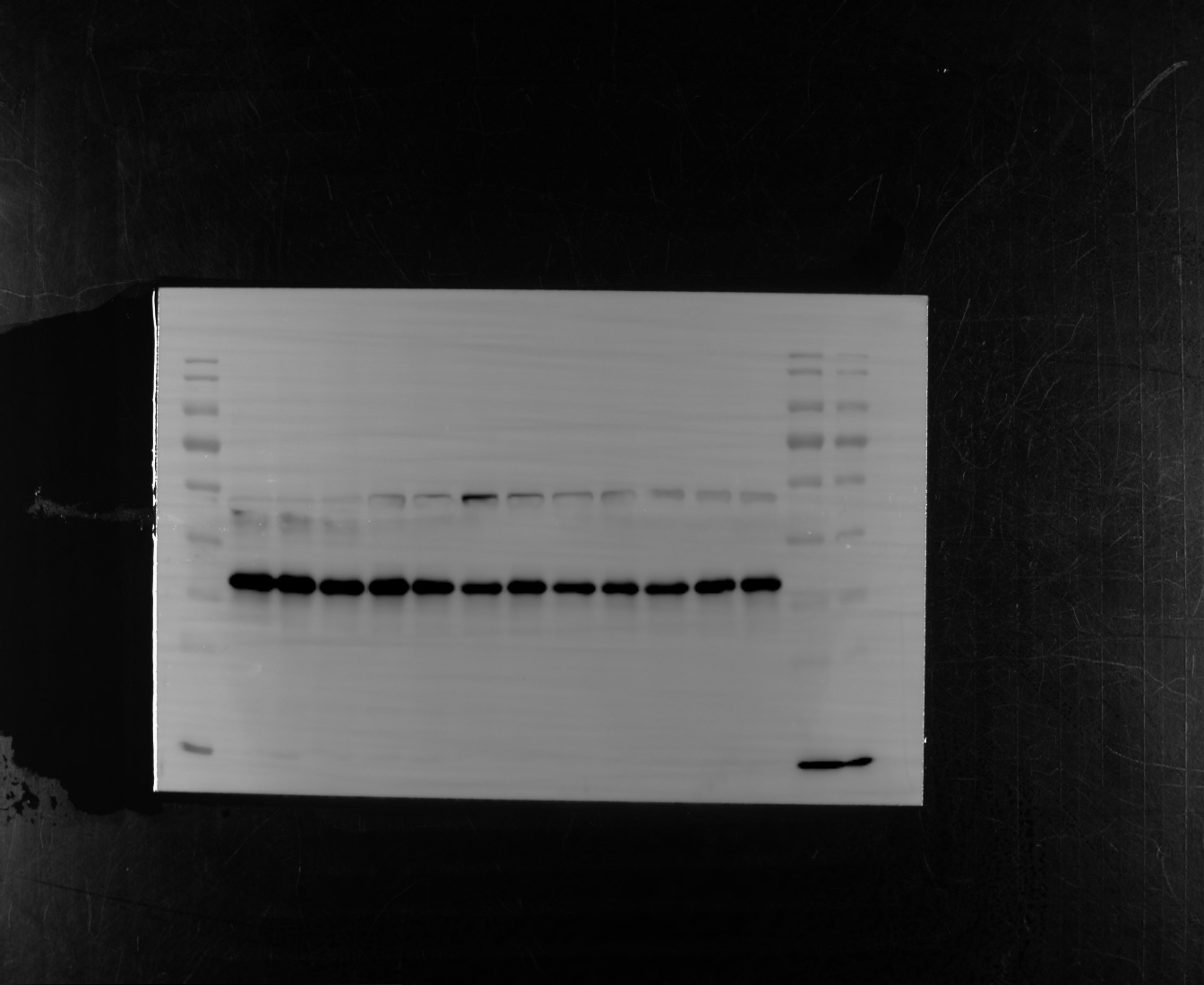

Supplement: Figure 8—source data 2. [file elife-99862-fig8-data2.zip › Figure 8-source data 2 jpgσ¢╛σ░Å/Figure 8G/ARP3/gapdh.jpg]

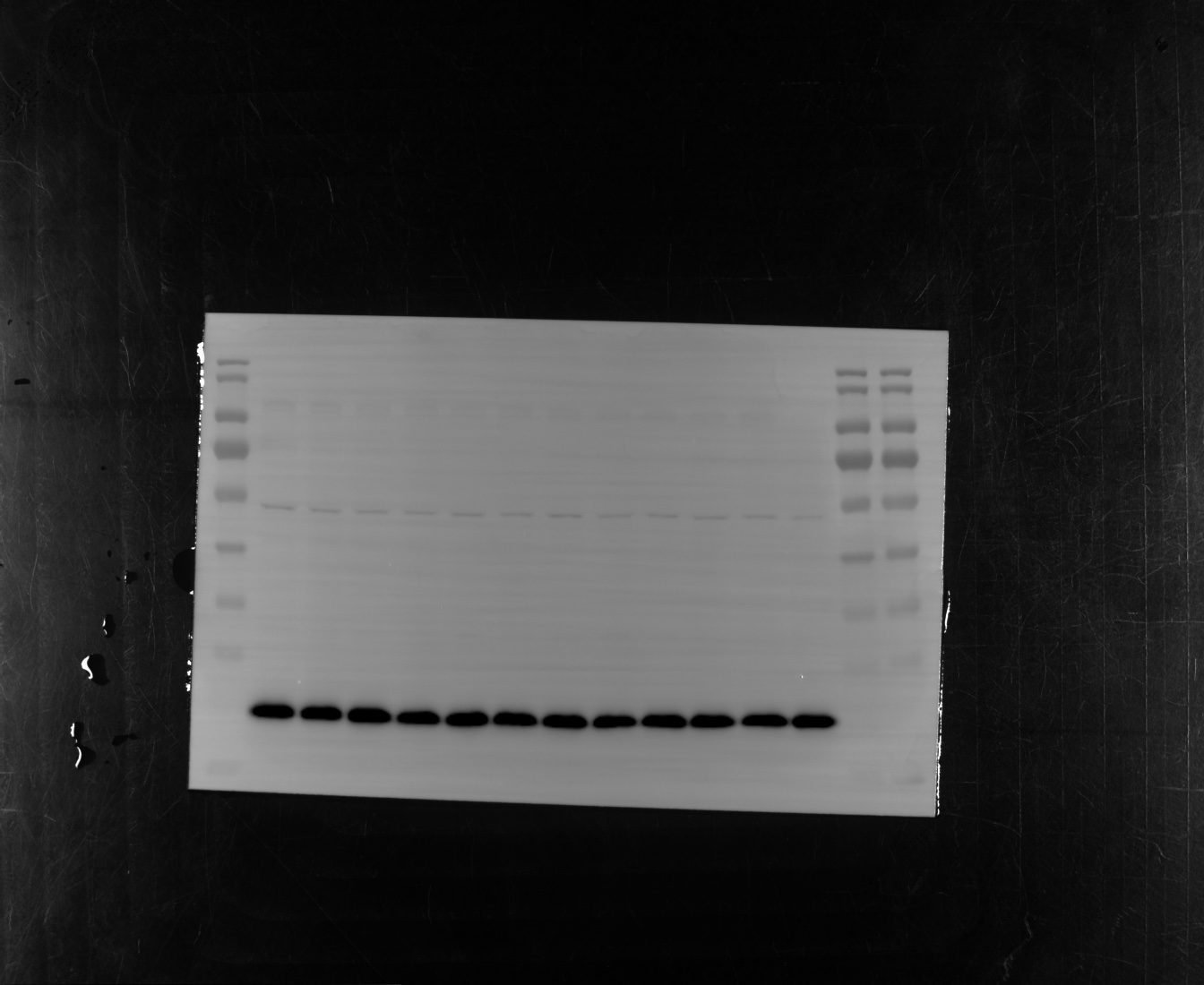

Supplement: Figure 8—source data 2. [file elife-99862-fig8-data2.zip › Figure 8-source data 2 jpgσ¢╛σ░Å/Figure 8C/RAC1/rac1.jpg]

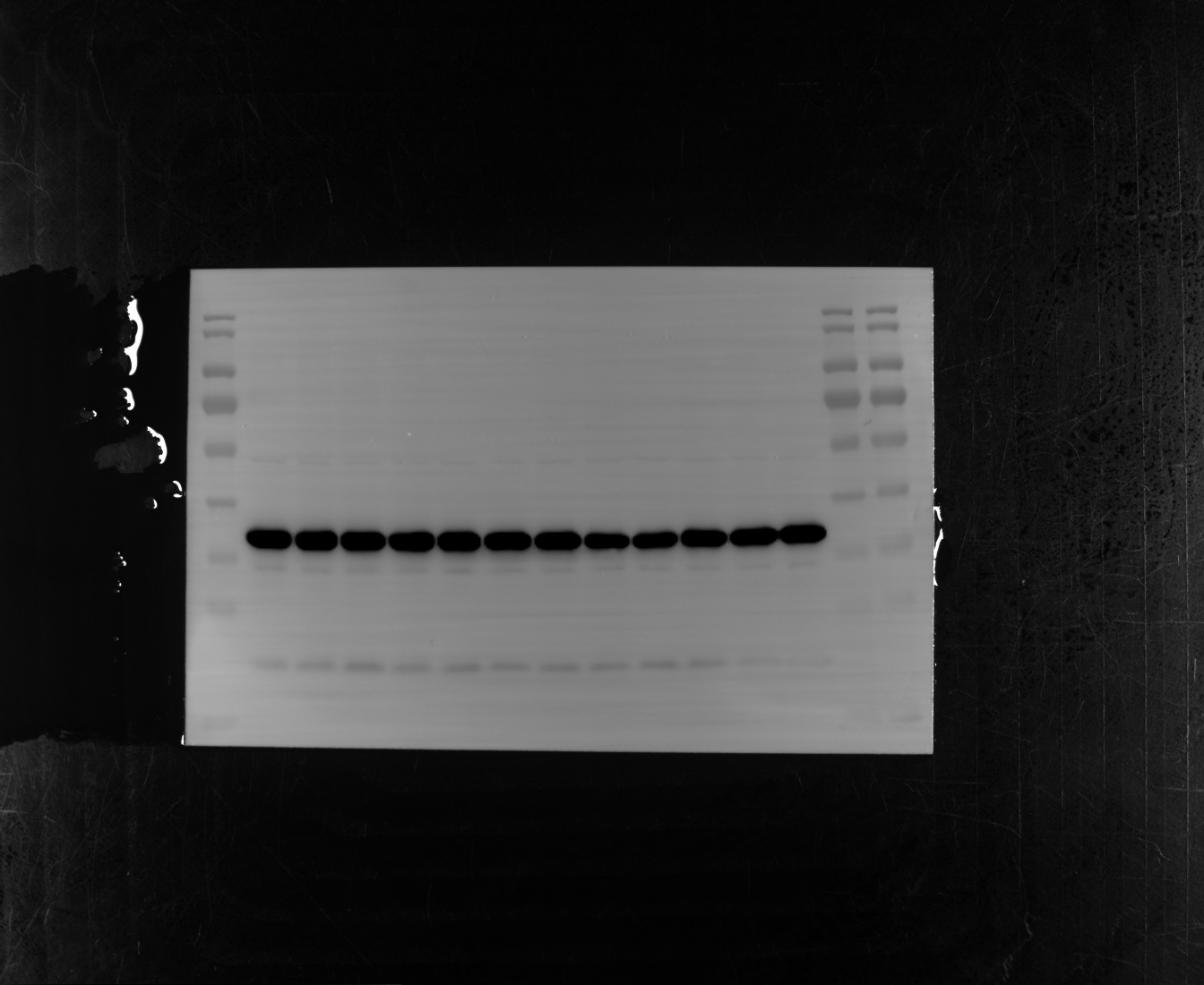

Supplement: Figure 8—source data 2. [file elife-99862-fig8-data2.zip › Figure 8-source data 2 jpgσ¢╛σ░Å/Figure 8C/RAC1/gapdh.jpg]

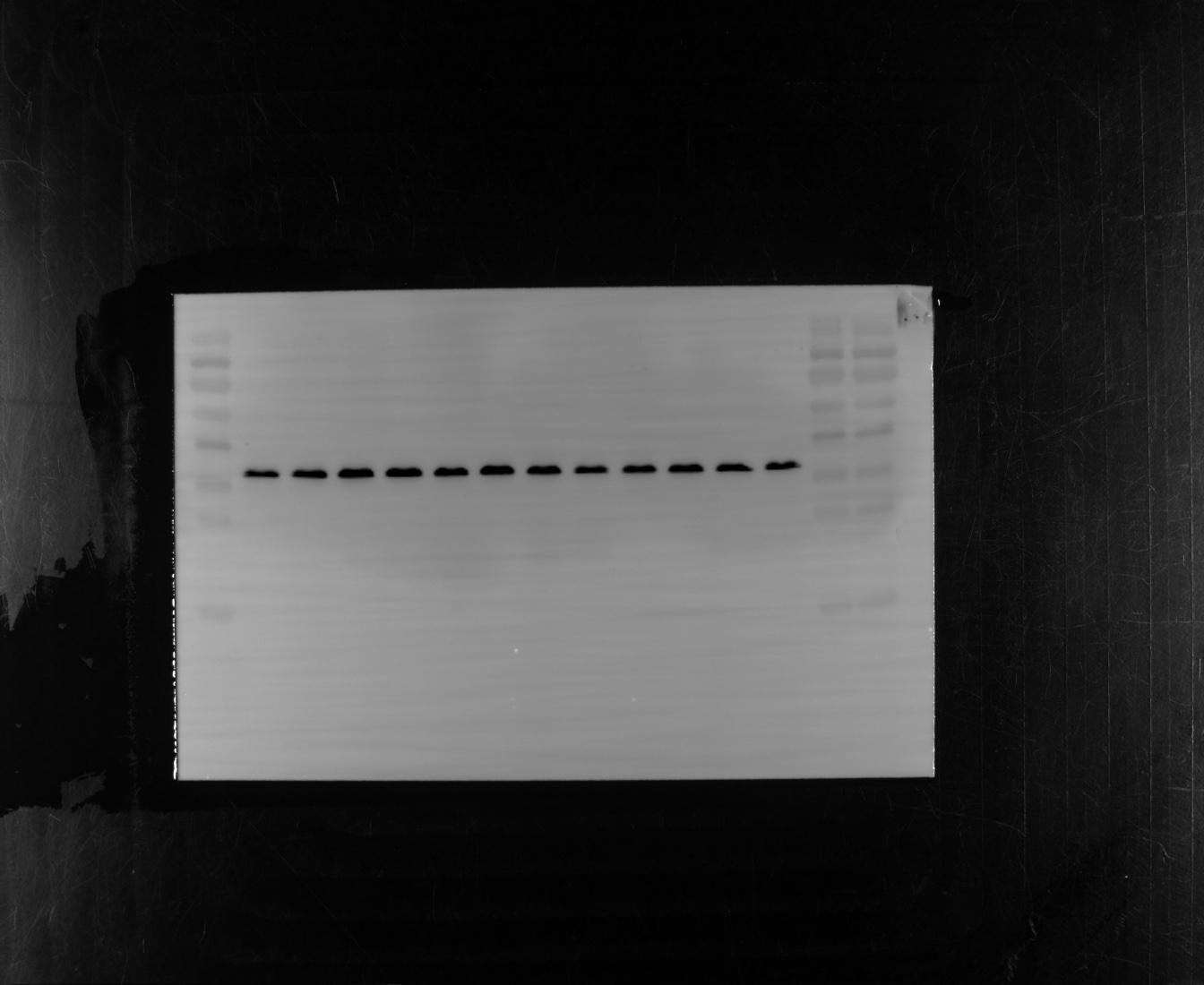

Supplement: Figure 8—source data 2. [file elife-99862-fig8-data2.zip › Figure 8-source data 2 jpgσ¢╛σ░Å/Figure 8C/CDC42/gapdh.jpg]

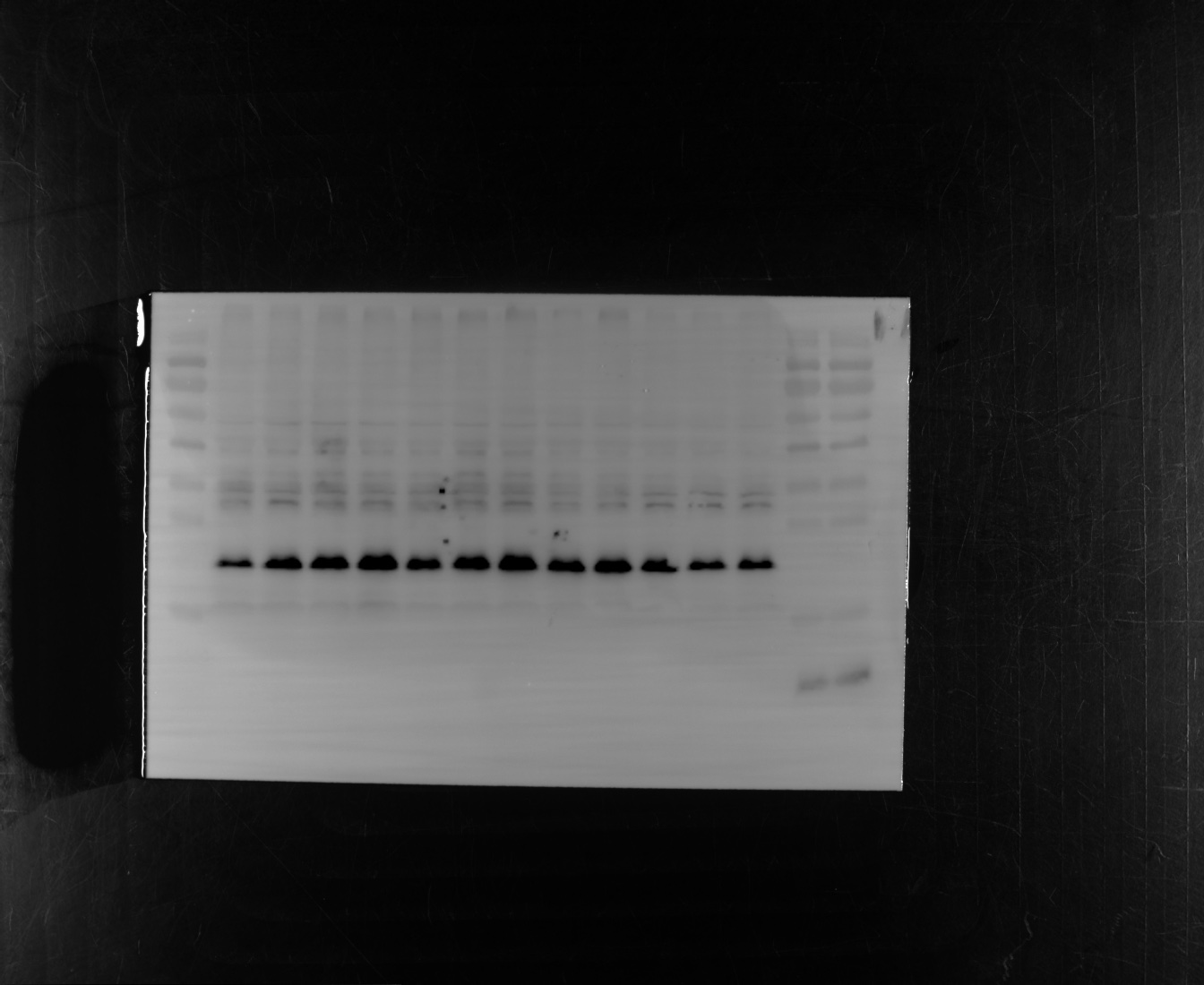

Supplement: Figure 8—source data 2. [file elife-99862-fig8-data2.zip › Figure 8-source data 2 jpgσ¢╛σ░Å/Figure 8C/CDC42/cdc42.jpg]

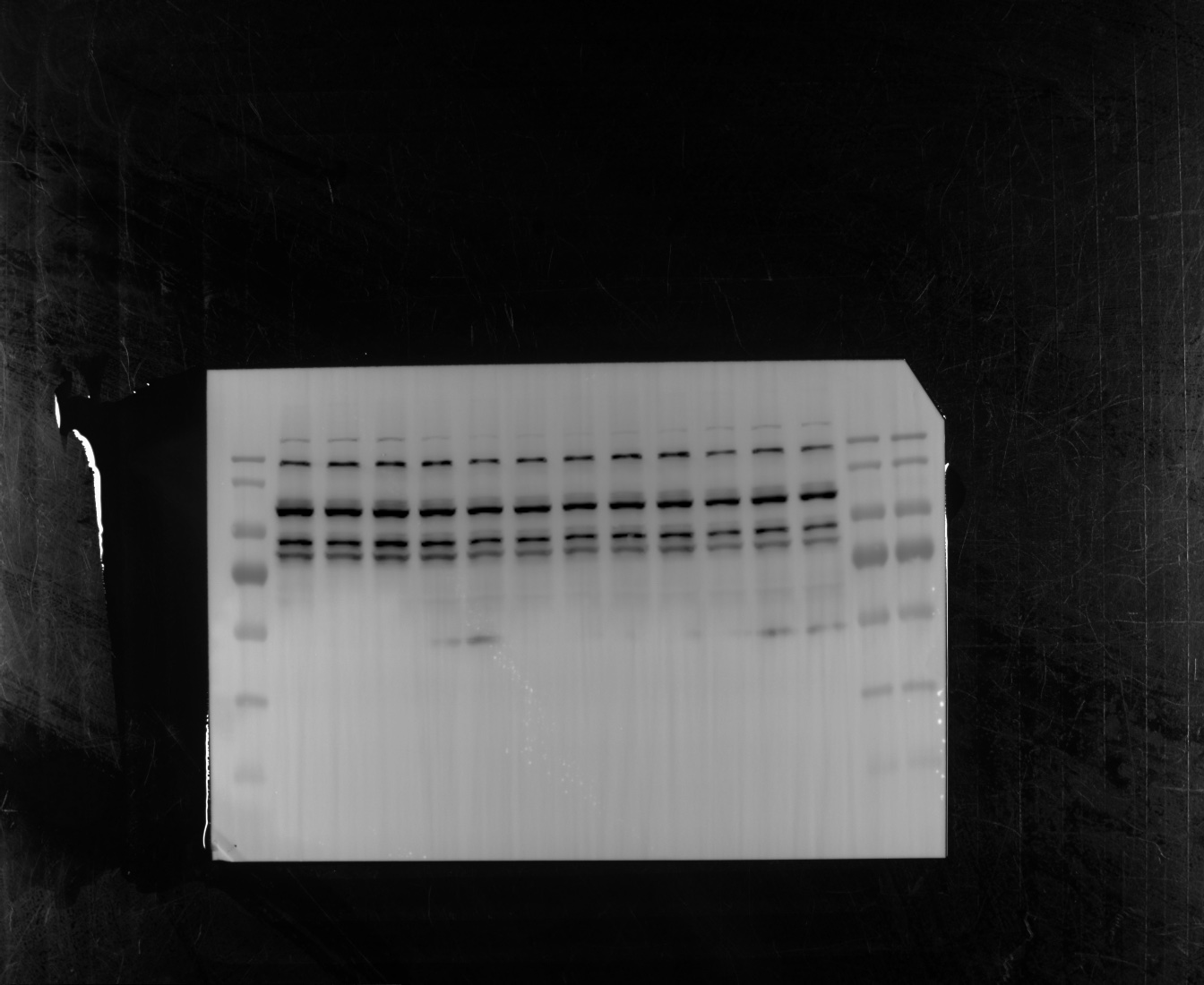

Supplement: Figure 8—source data 2. [file elife-99862-fig8-data2.zip › Figure 8-source data 2 jpgσ¢╛σ░Å/Figure 8C/ITGA2/itga2.jpg]

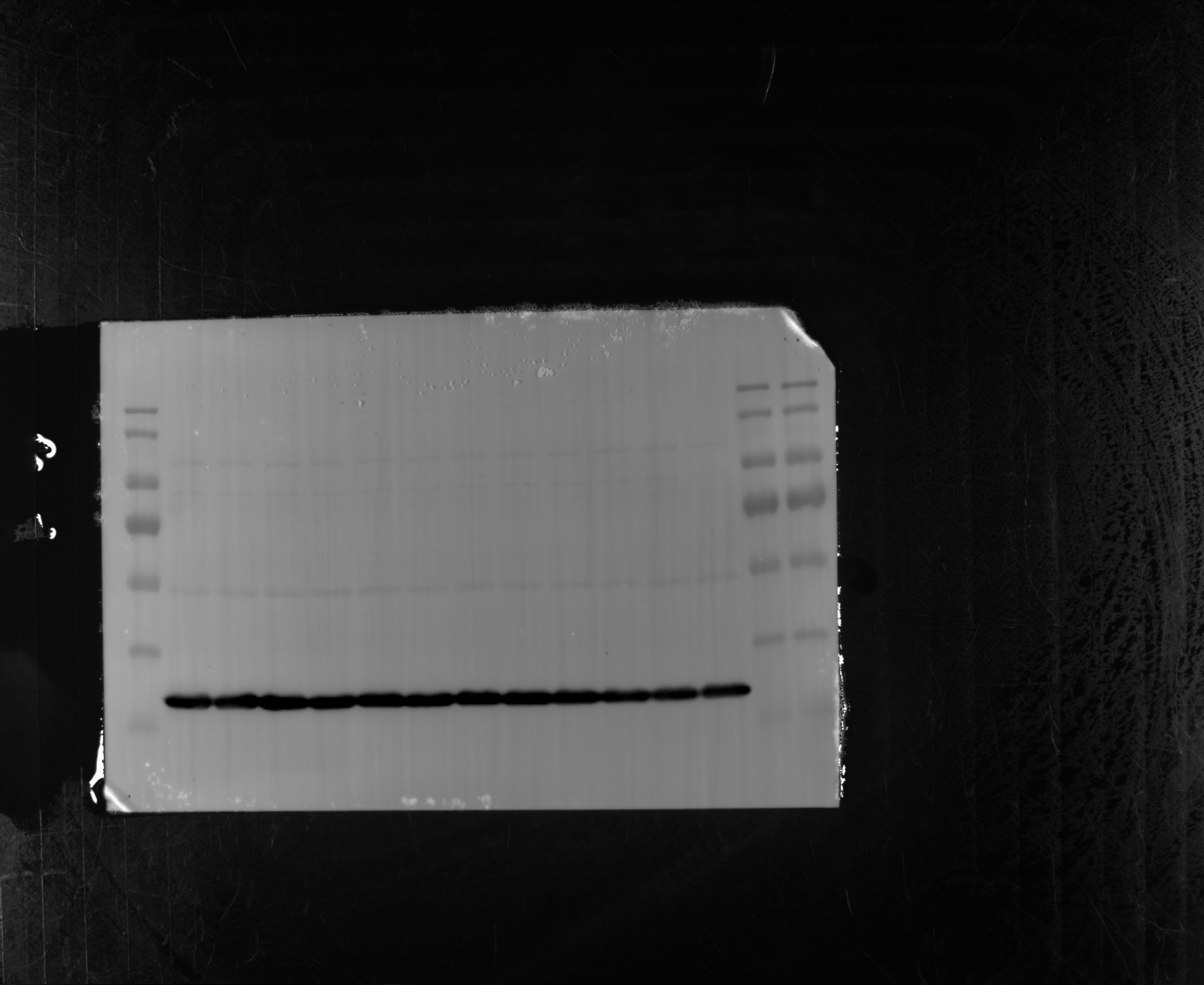

Supplement: Figure 8—source data 2. [file elife-99862-fig8-data2.zip › Figure 8-source data 2 jpgσ¢╛σ░Å/Figure 8C/ITGA2/gapdh.jpg]

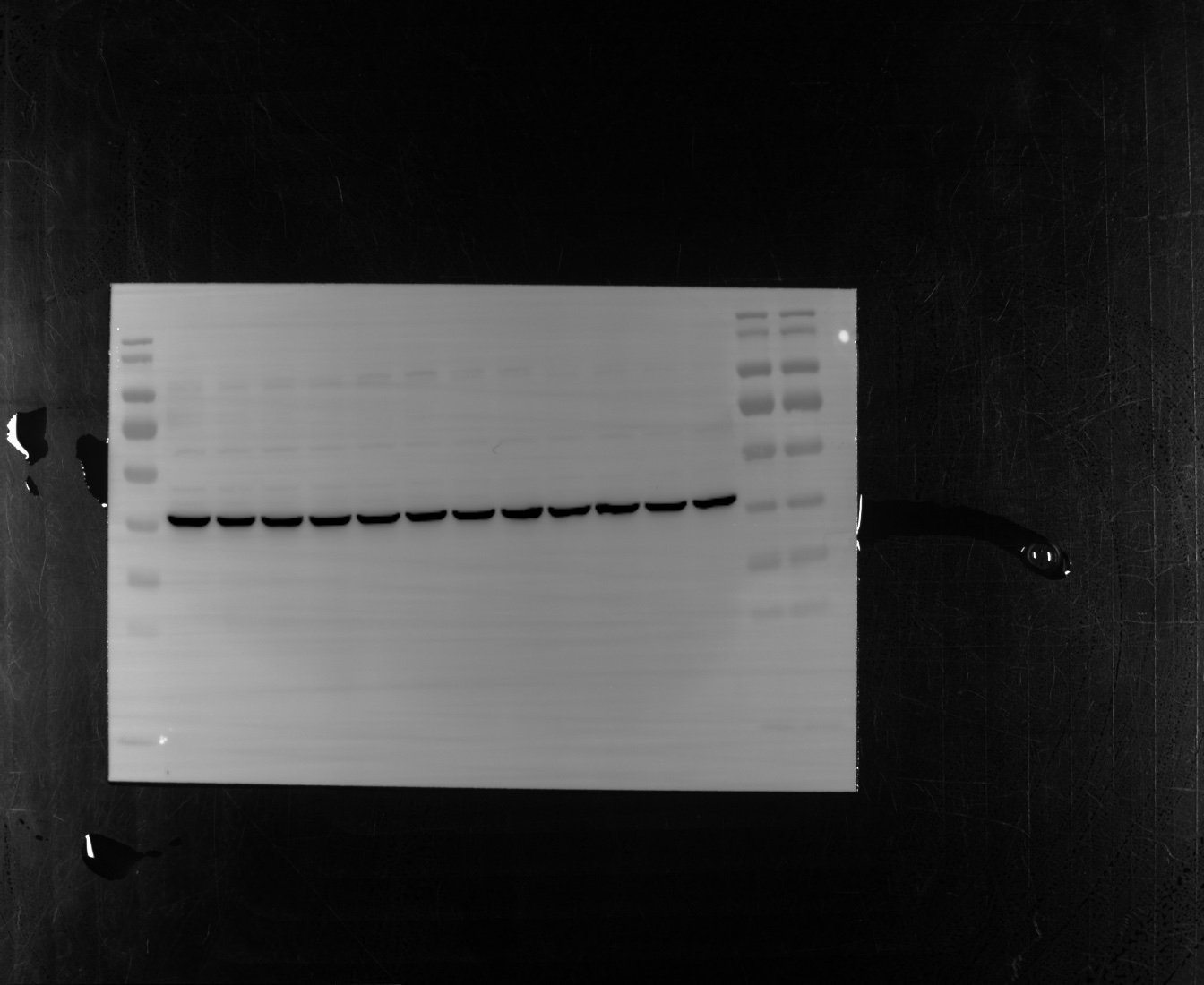

Supplement: Figure 8—source data 2. [file elife-99862-fig8-data2.zip › Figure 8-source data 2 jpgσ¢╛σ░Å/Figure 8C/ARP2/arp2.jpg]

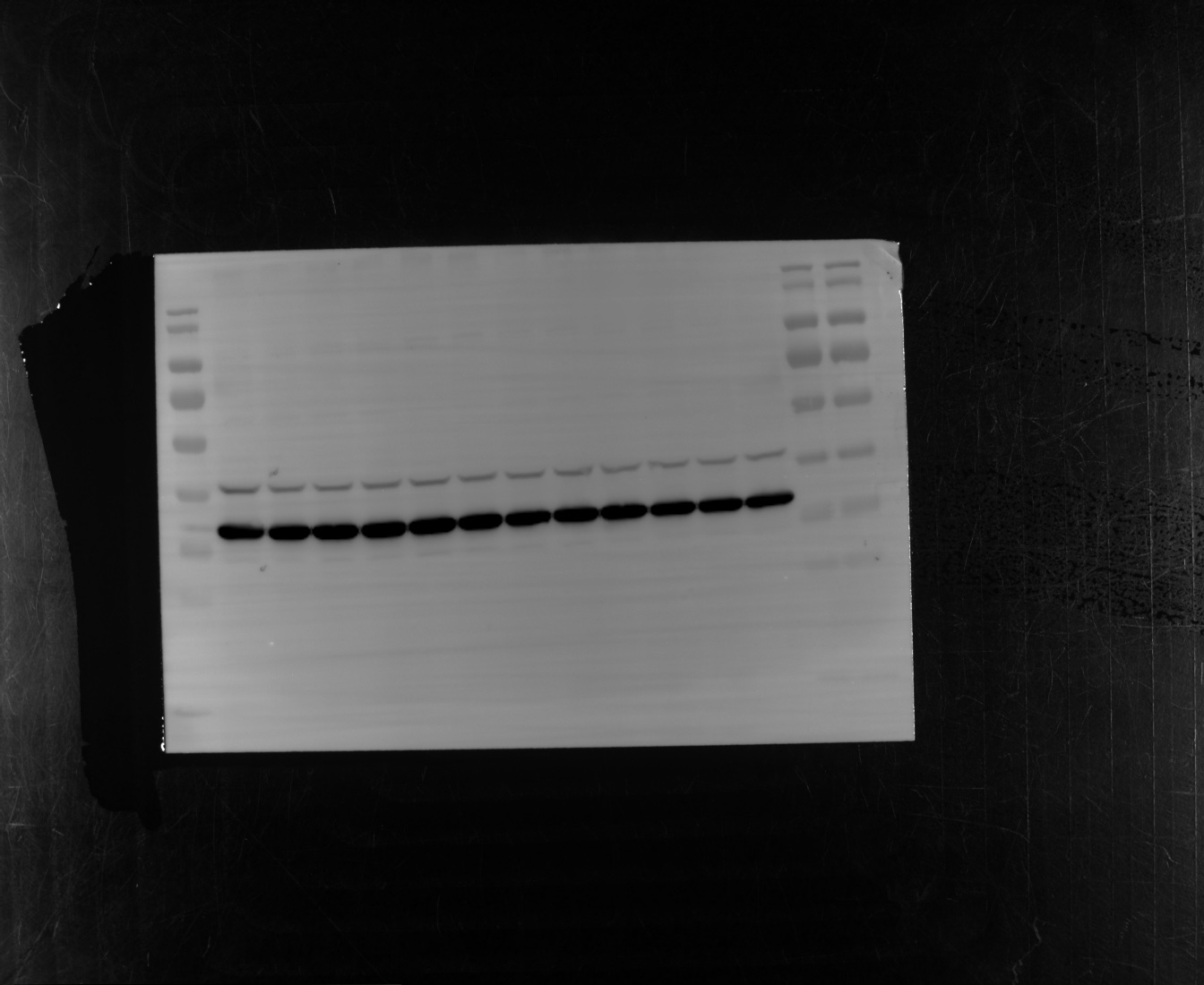

Supplement: Figure 8—source data 2. [file elife-99862-fig8-data2.zip › Figure 8-source data 2 jpgσ¢╛σ░Å/Figure 8C/ARP2/gapdh.jpg]

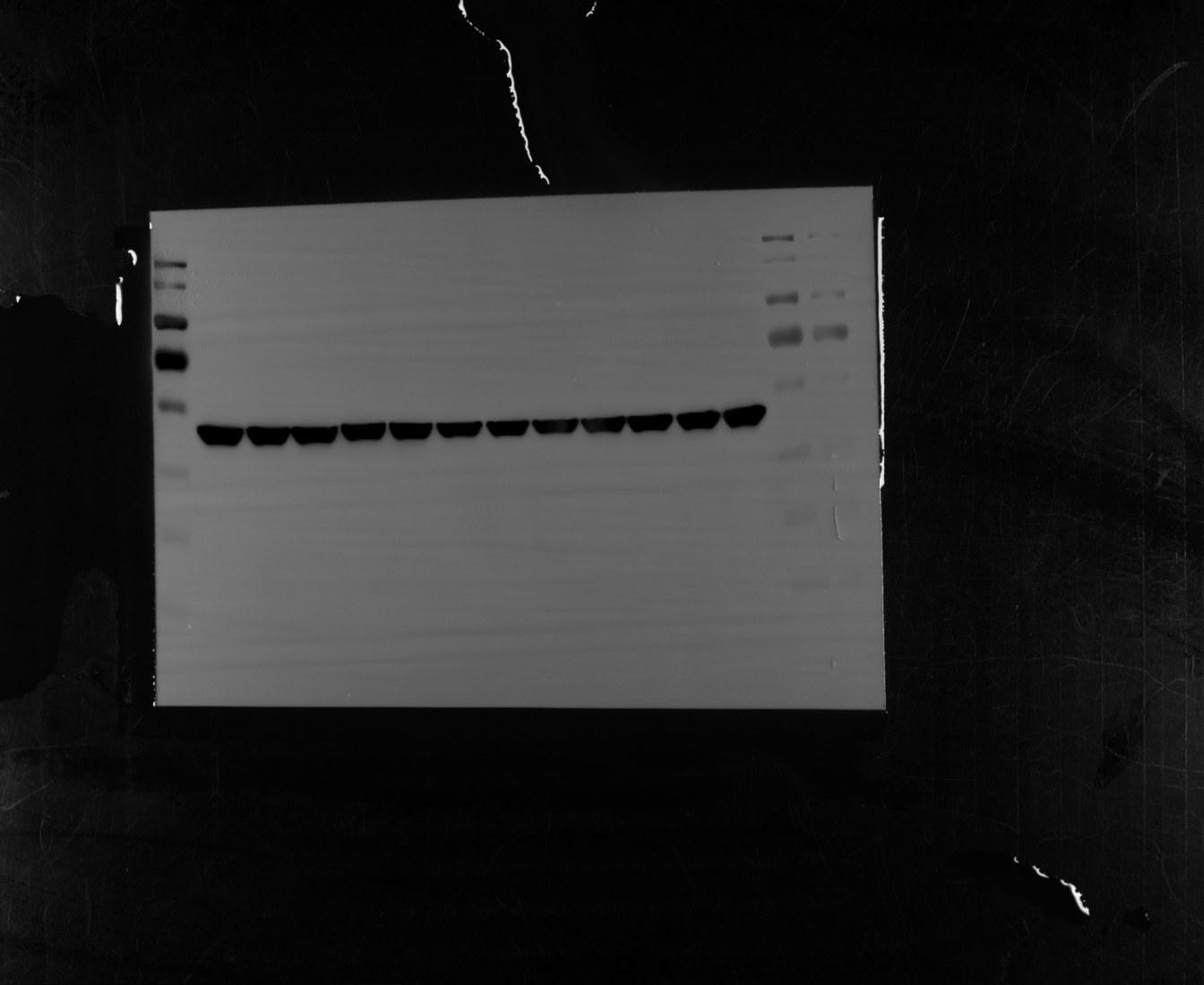

Supplement: Figure 8—source data 2. [file elife-99862-fig8-data2.zip › Figure 8-source data 2 jpgσ¢╛σ░Å/Figure 8C/ARP3/arp3.jpg]

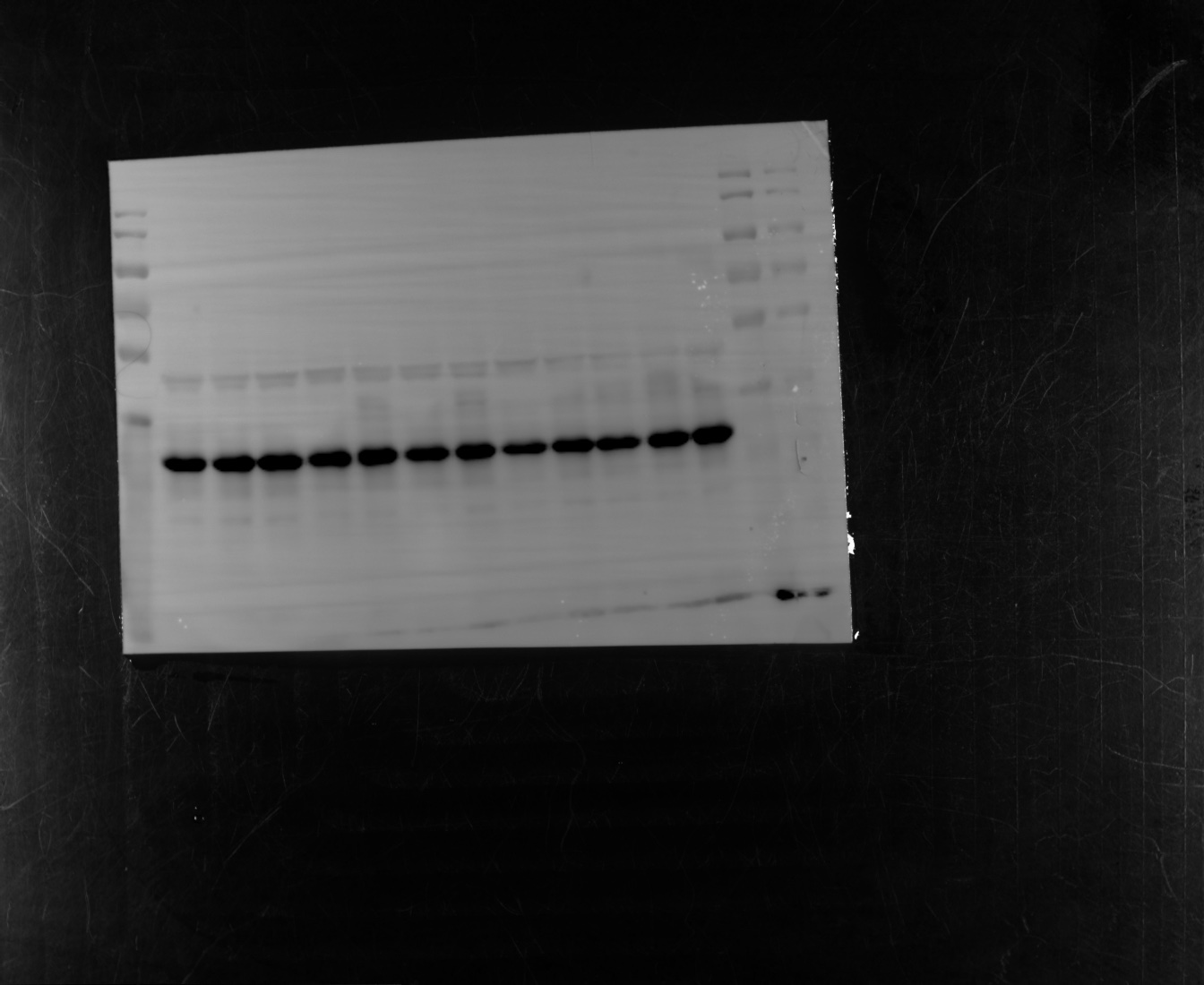

Supplement: Figure 8—source data 2. [file elife-99862-fig8-data2.zip › Figure 8-source data 2 jpgσ¢╛σ░Å/Figure 8C/ARP3/GAPDH.jpg]

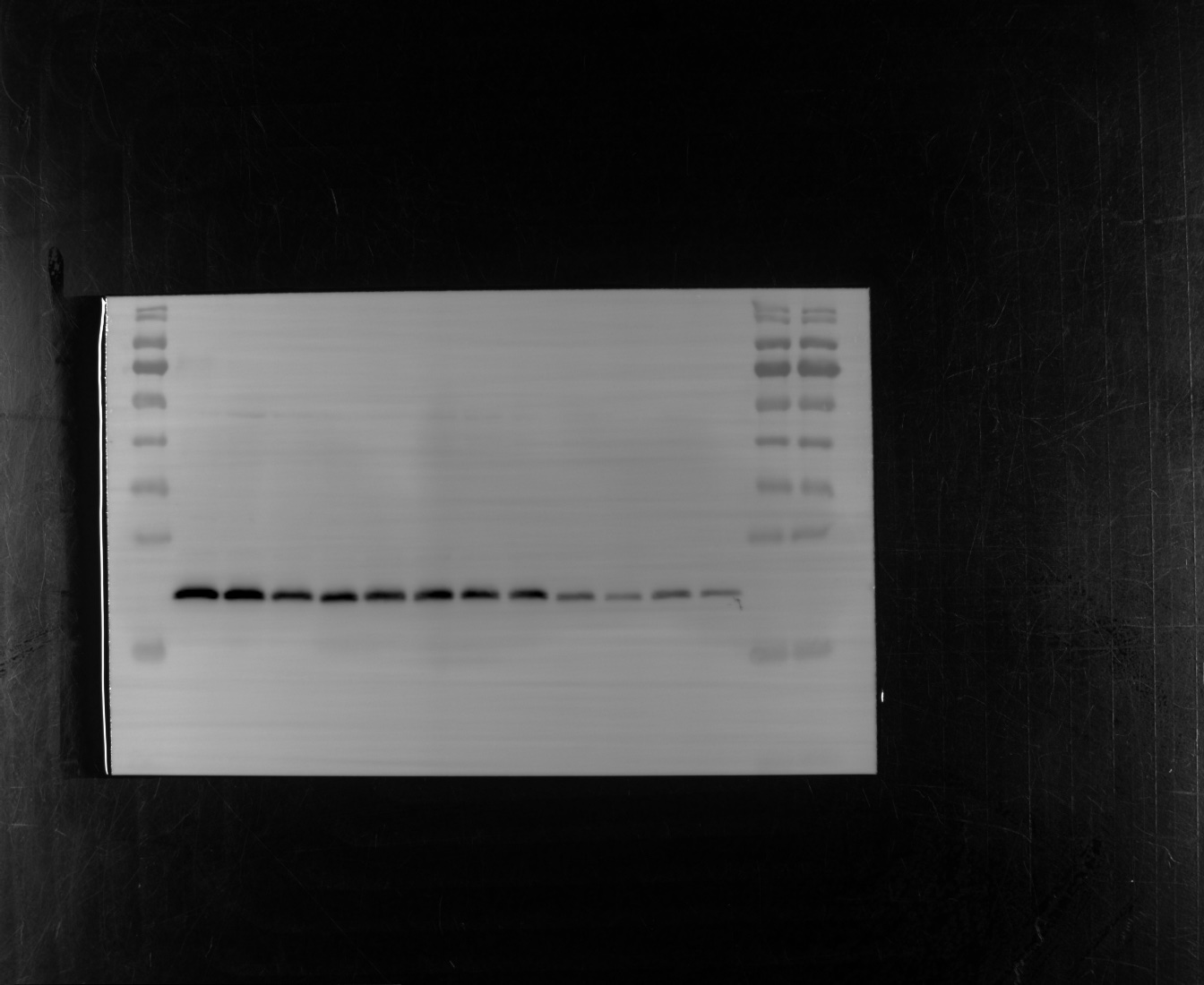

Supplement: Figure 8—source data 2. [file elife-99862-fig8-data2.zip › Figure 8-source data 2 jpgσ¢╛σ░Å/Figure 8E/RAC1/rac1 1.jpg]

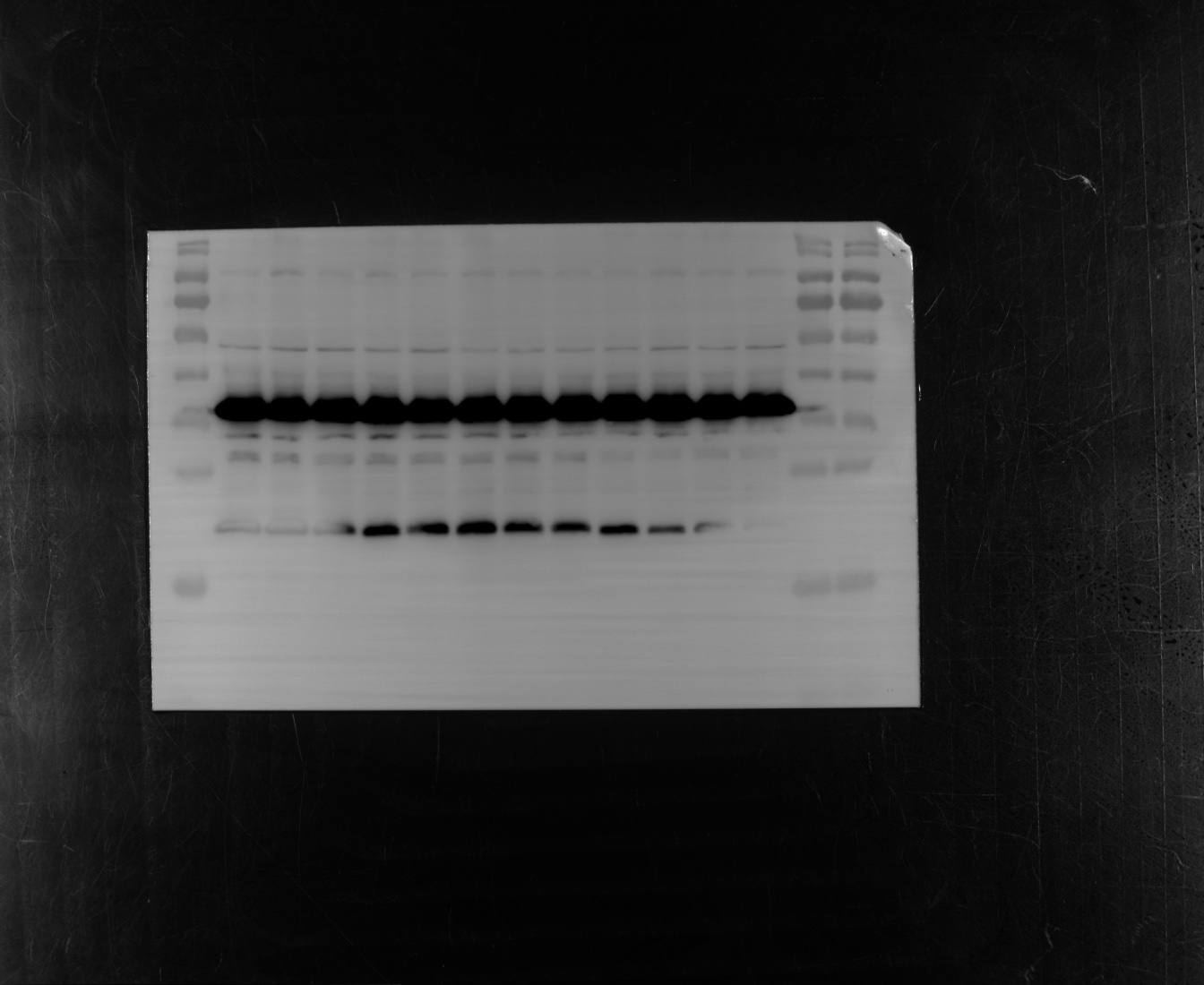

Supplement: Figure 8—source data 2. [file elife-99862-fig8-data2.zip › Figure 8-source data 2 jpgσ¢╛σ░Å/Figure 8E/RAC1/gapdh 1.jpg]

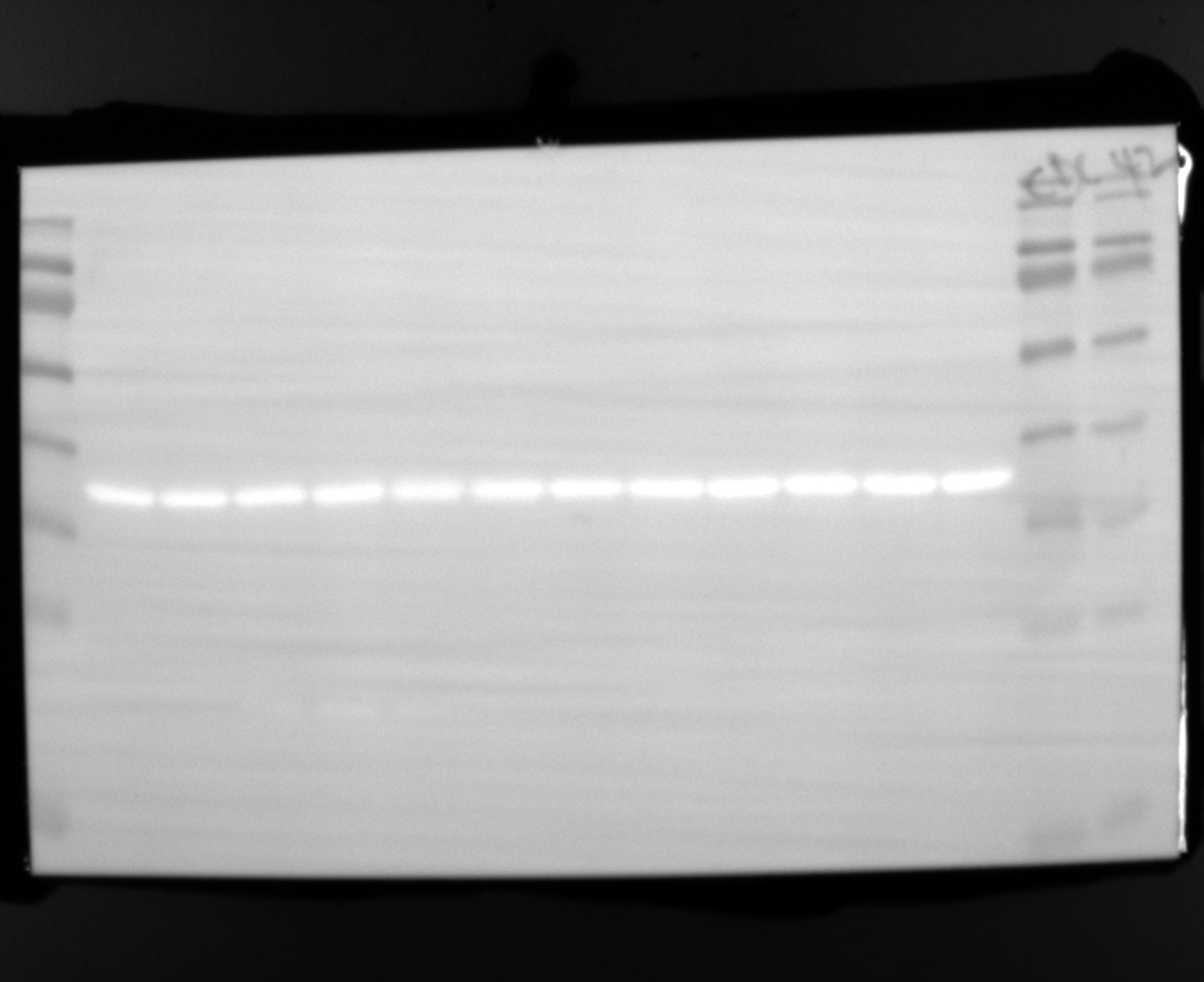

Supplement: Figure 8—source data 2. [file elife-99862-fig8-data2.zip › Figure 8-source data 2 jpgσ¢╛σ░Å/Figure 8E/CDC42/gapdh.jpg]

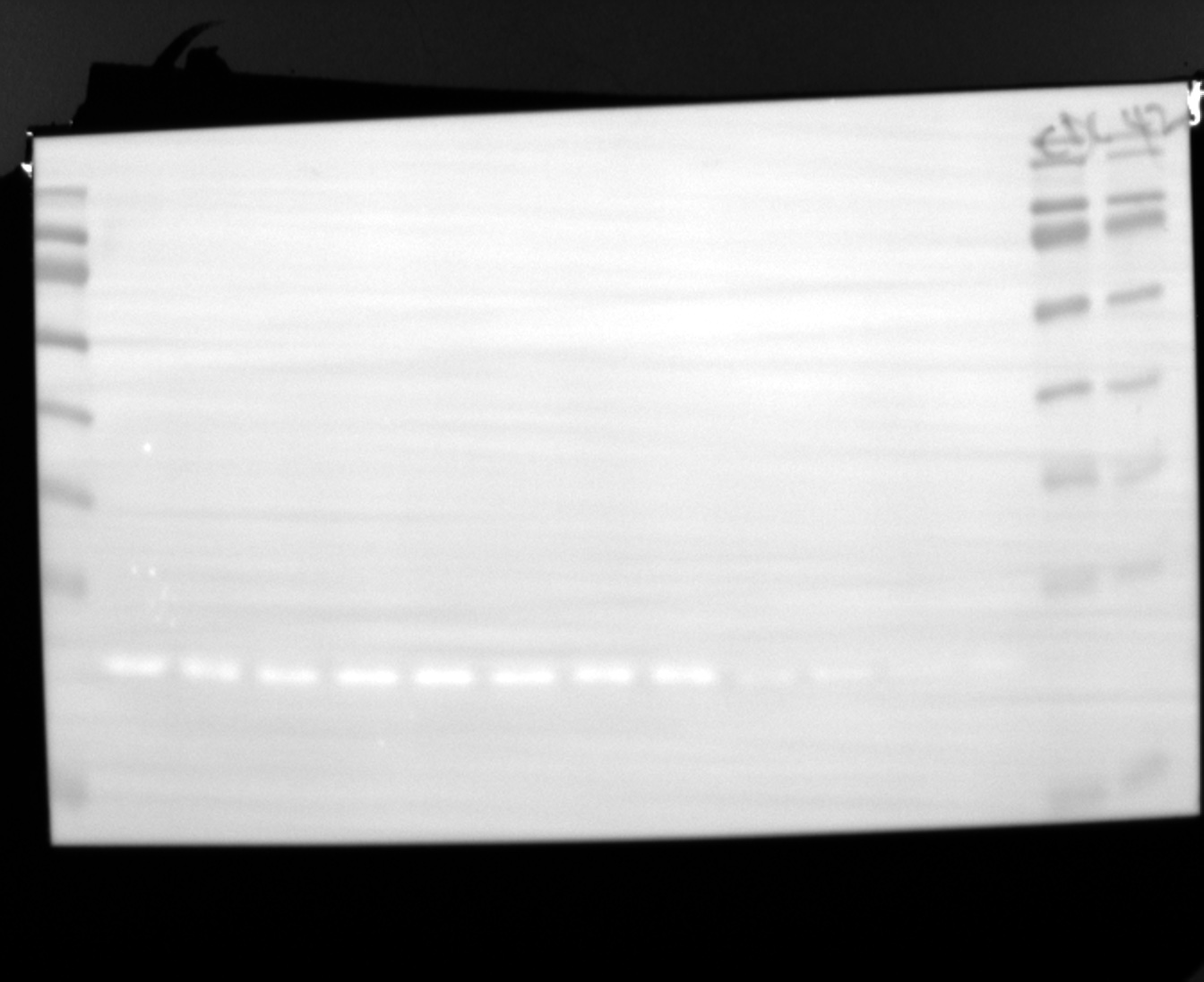

Supplement: Figure 8—source data 2. [file elife-99862-fig8-data2.zip › Figure 8-source data 2 jpgσ¢╛σ░Å/Figure 8E/CDC42/cdc42.jpg]

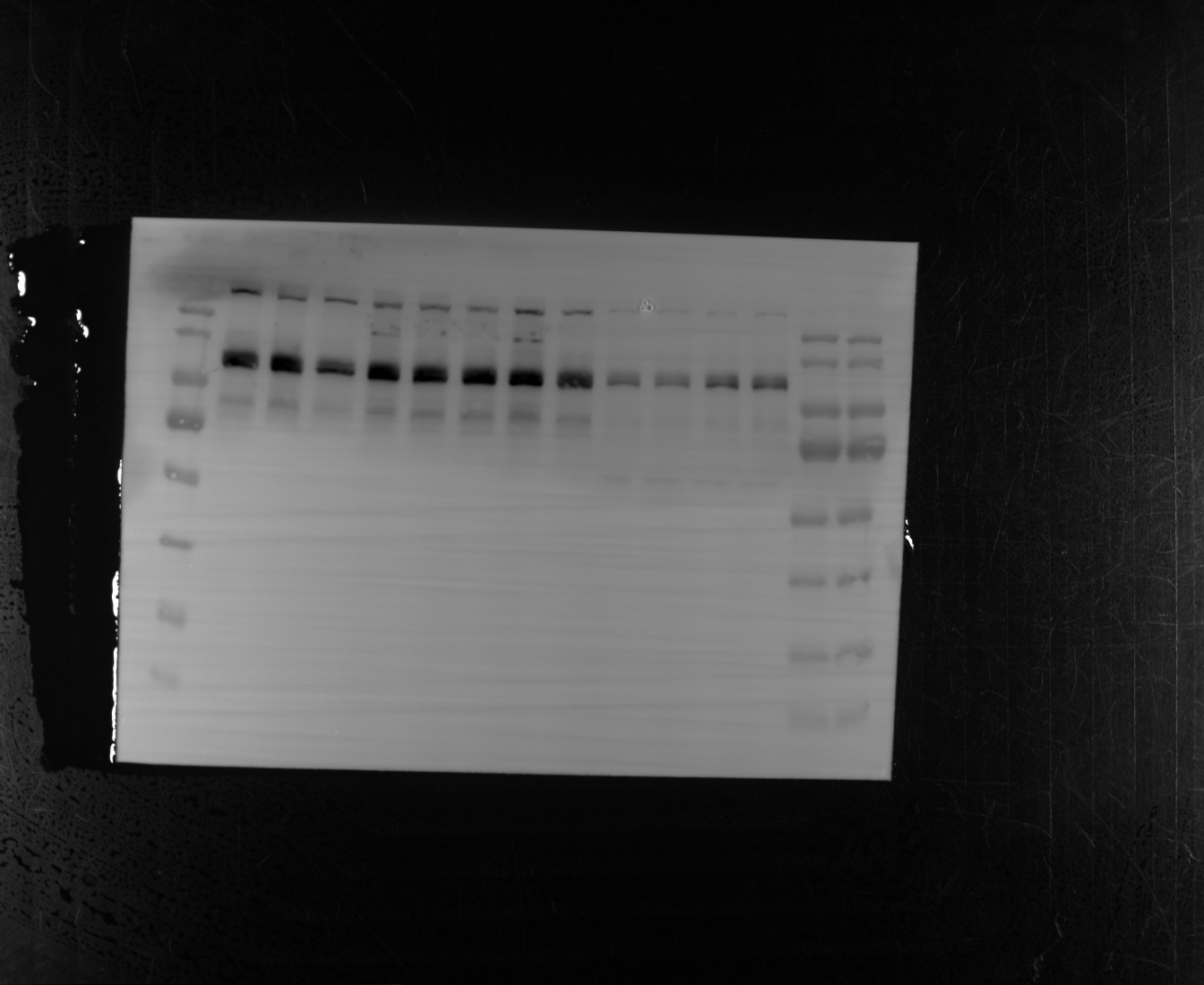

Supplement: Figure 8—source data 2. [file elife-99862-fig8-data2.zip › Figure 8-source data 2 jpgσ¢╛σ░Å/Figure 8E/ITGA2/itga2.jpg]

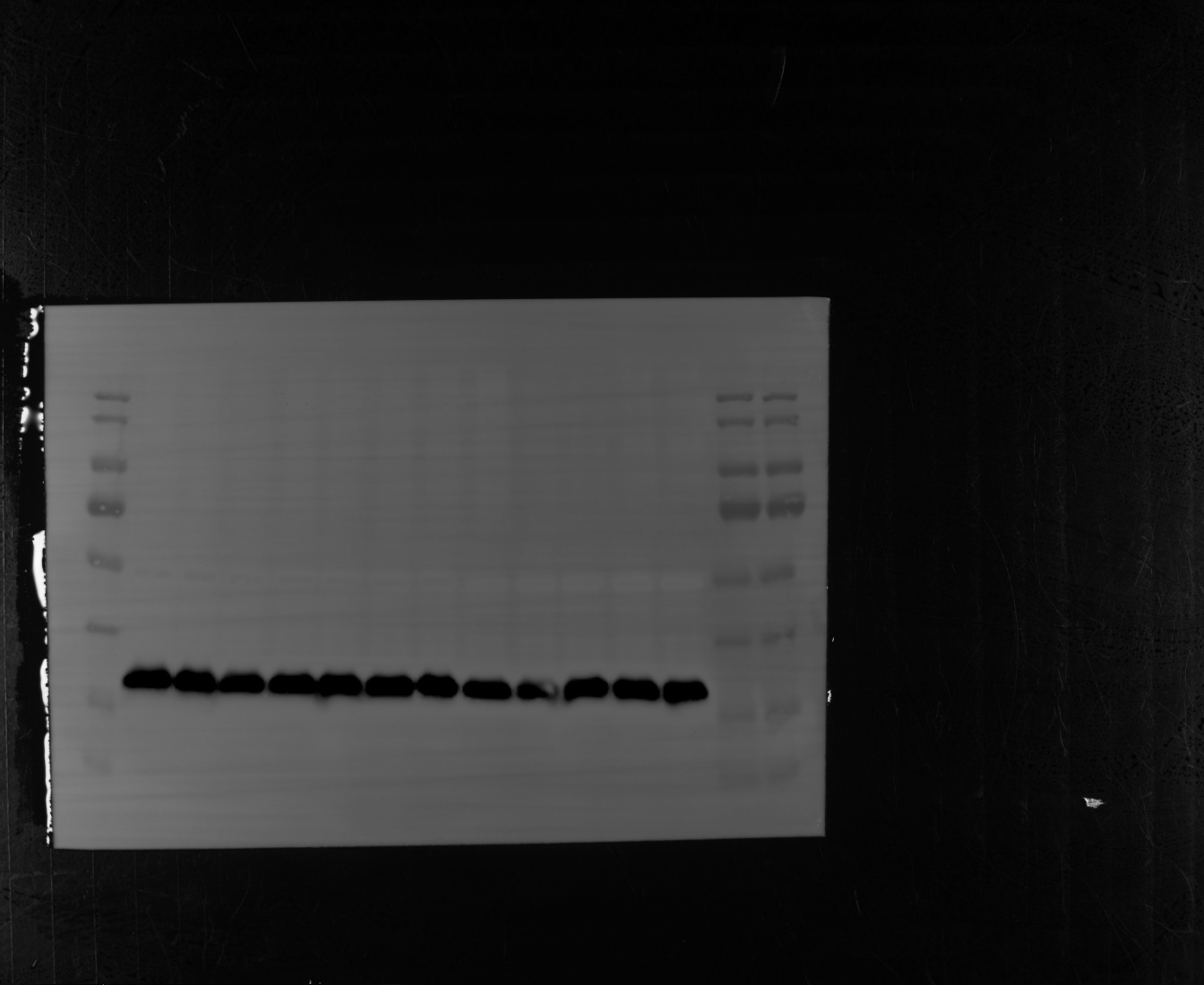

Supplement: Figure 8—source data 2. [file elife-99862-fig8-data2.zip › Figure 8-source data 2 jpgσ¢╛σ░Å/Figure 8E/ITGA2/gapdh.jpg]

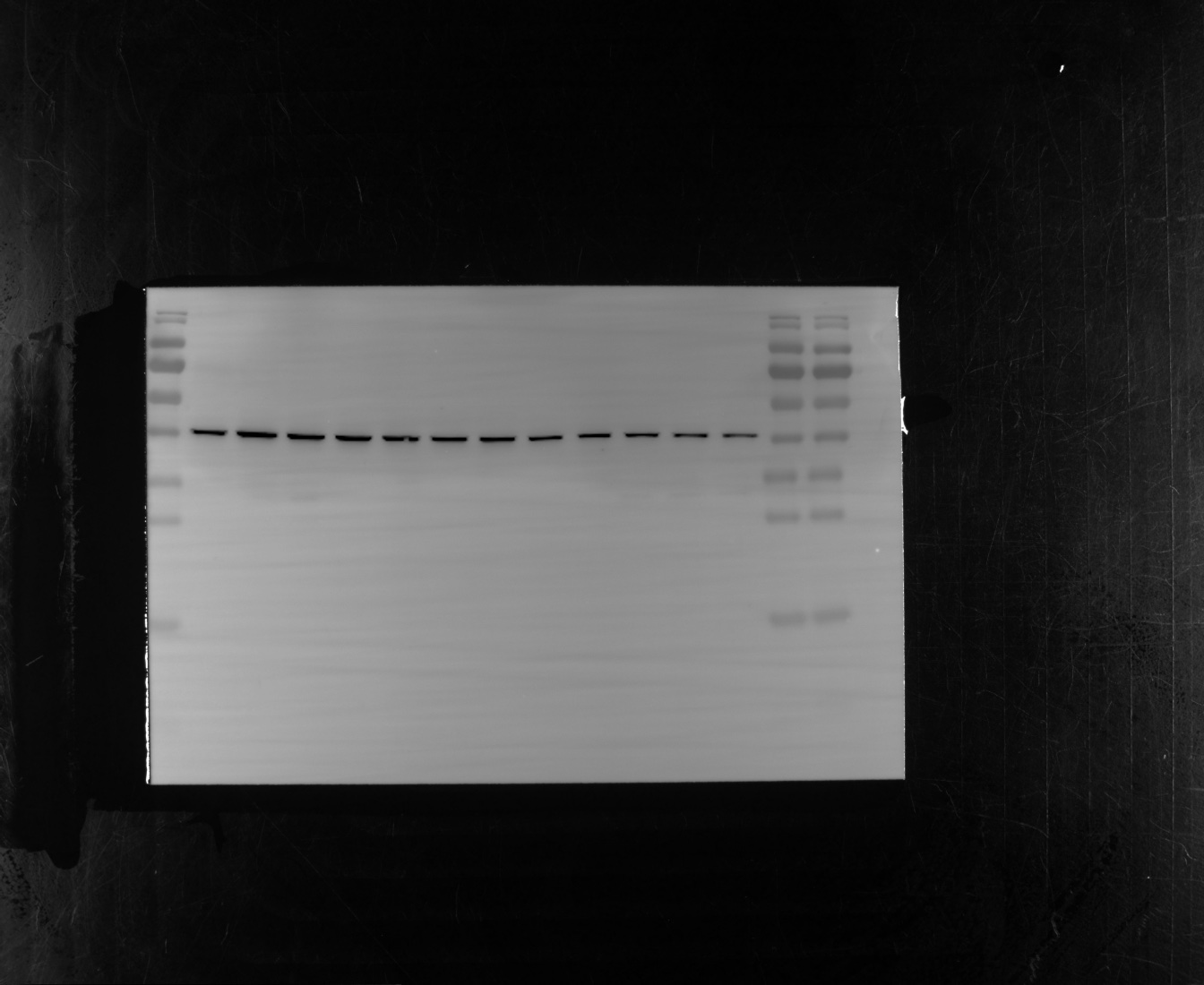

Supplement: Figure 8—source data 2. [file elife-99862-fig8-data2.zip › Figure 8-source data 2 jpgσ¢╛σ░Å/Figure 8E/ARP2/arp2.jpg]

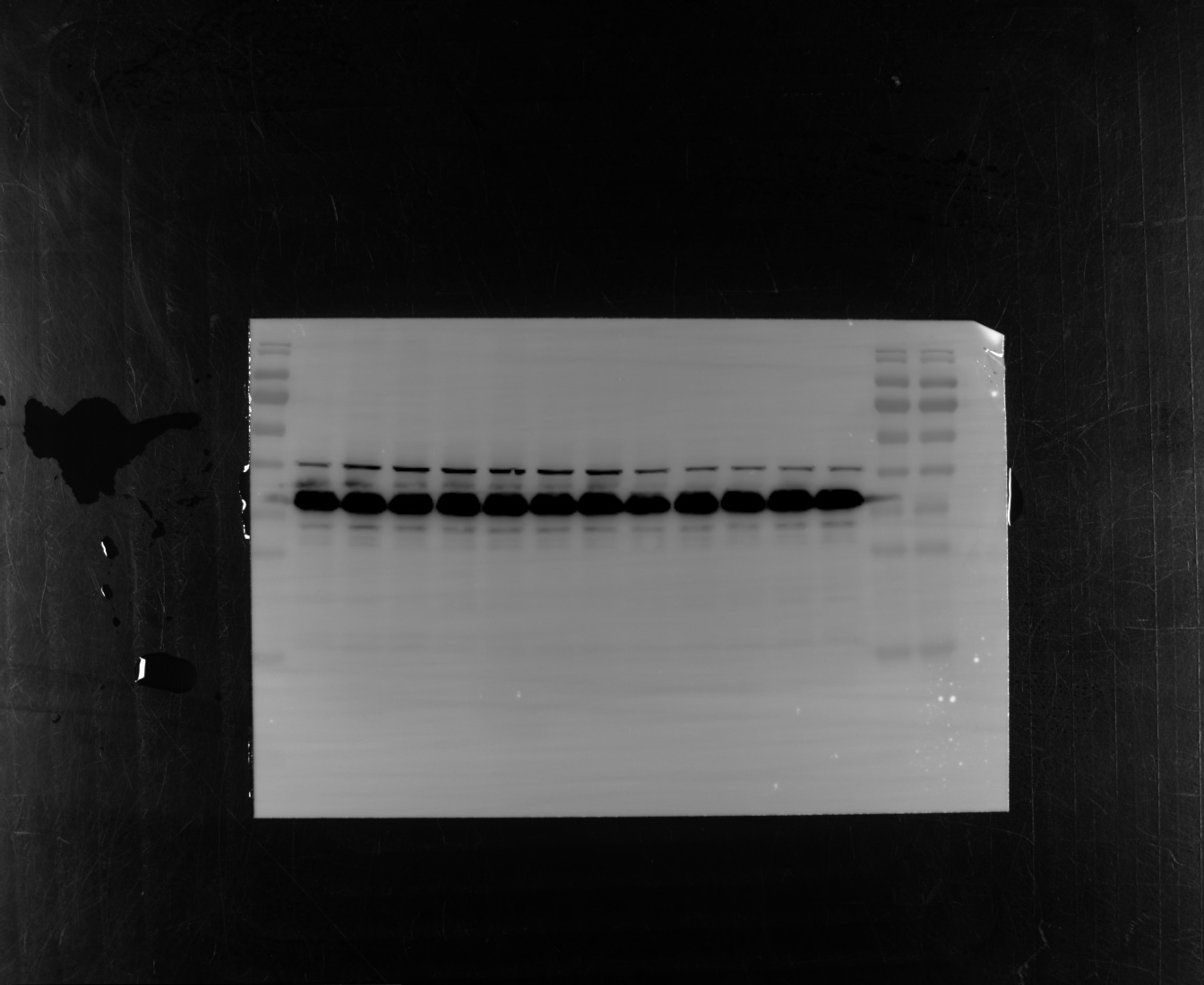

Supplement: Figure 8—source data 2. [file elife-99862-fig8-data2.zip › Figure 8-source data 2 jpgσ¢╛σ░Å/Figure 8E/ARP2/gapdh.jpg]

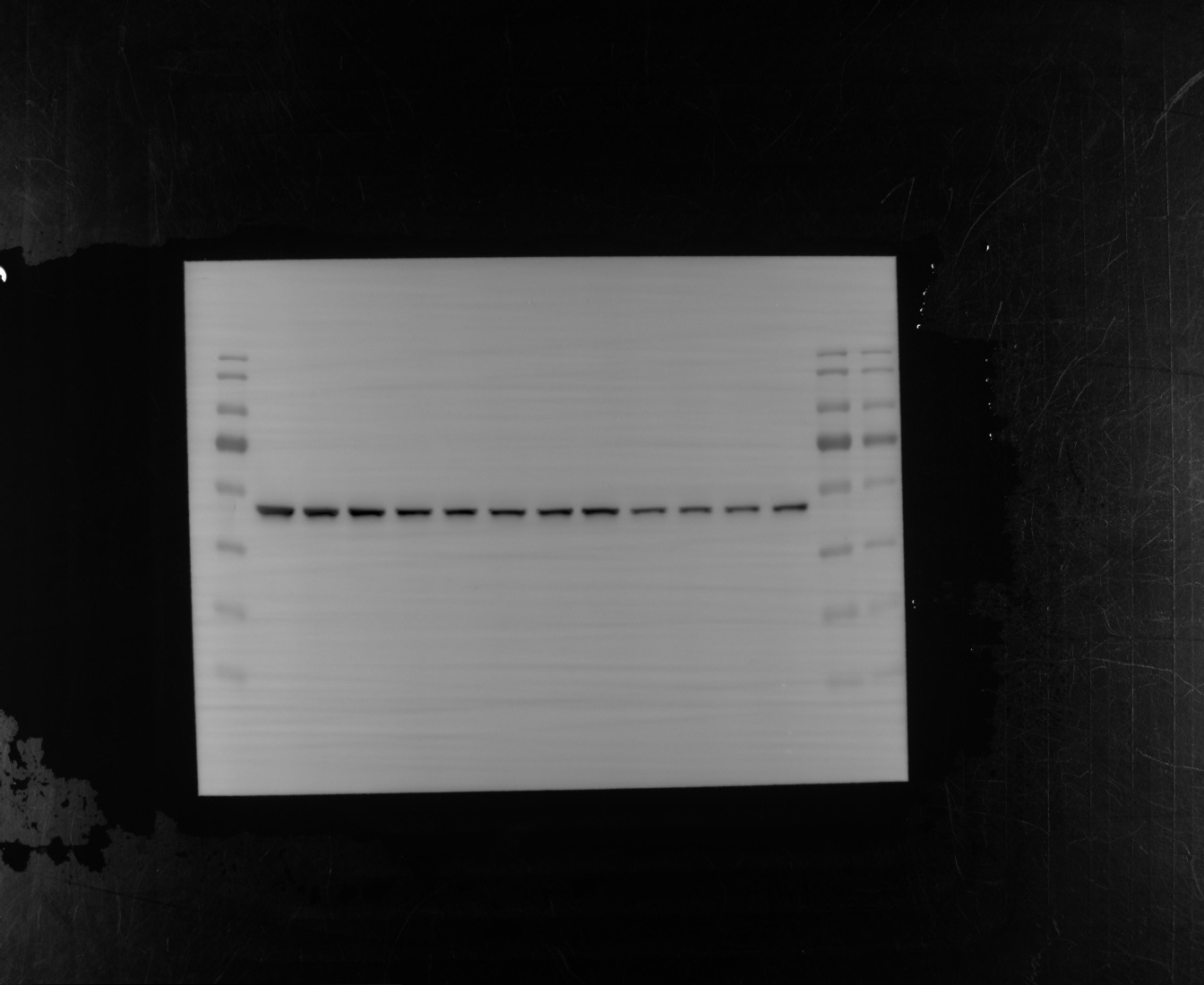

Supplement: Figure 8—source data 2. [file elife-99862-fig8-data2.zip › Figure 8-source data 2 jpgσ¢╛σ░Å/Figure 8E/ARP3/arp3.jpg]

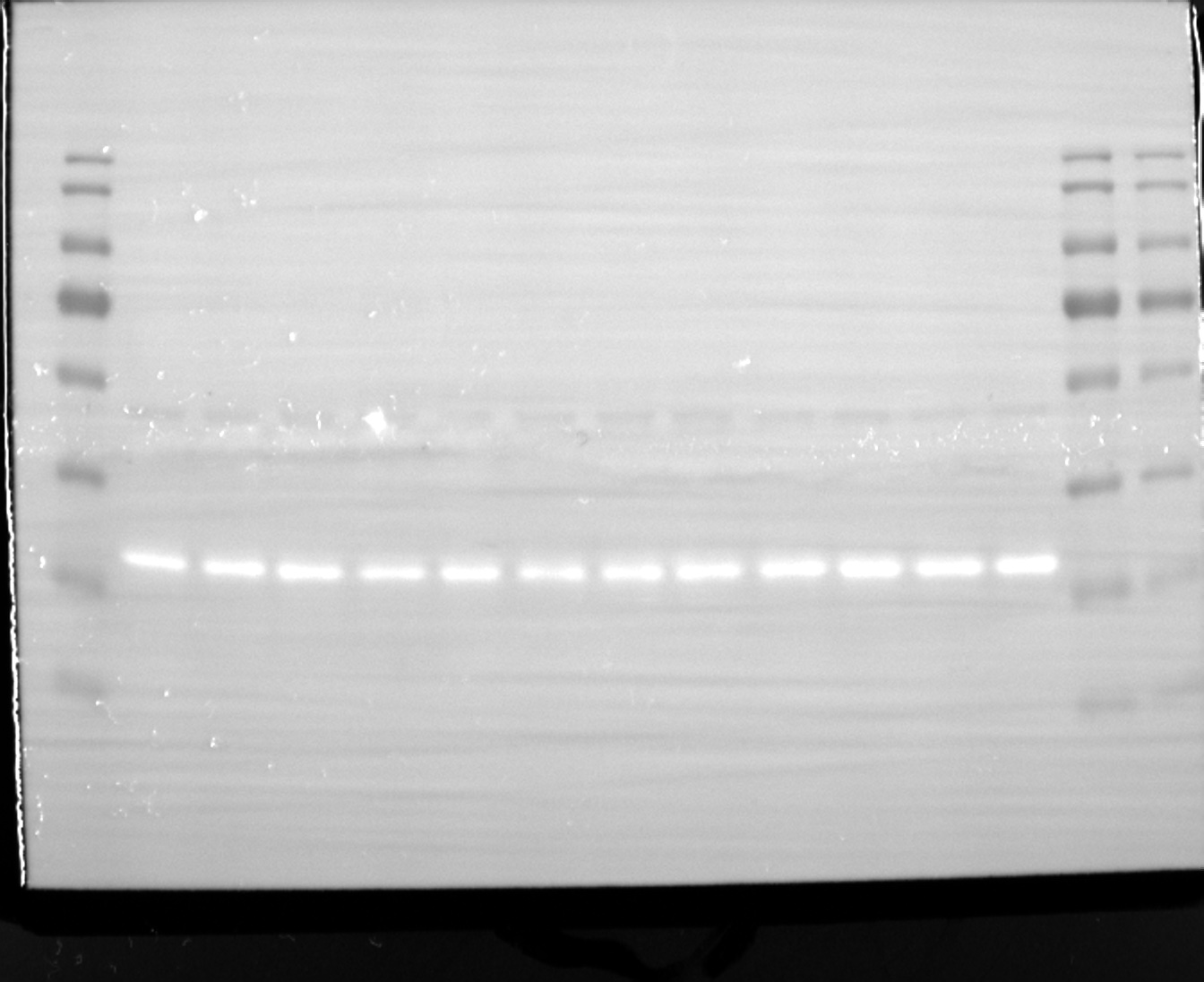

Supplement: Figure 8—source data 2. [file elife-99862-fig8-data2.zip › Figure 8-source data 2 jpgσ¢╛σ░Å/Figure 8E/ARP3/gapdh.jpg]

CCI-Acute

(1) RhoA

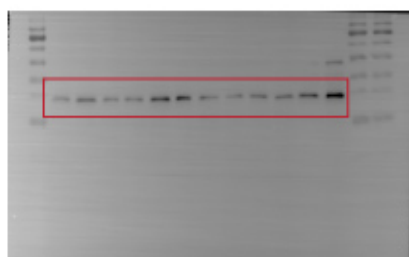

RhoA

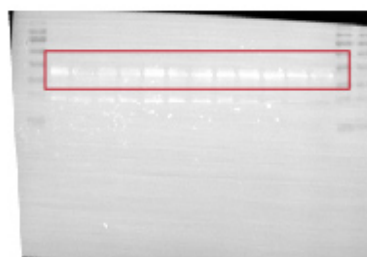

GAPDH

(2) S1PR2

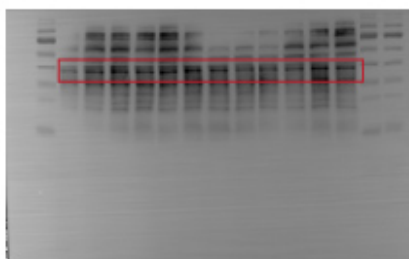

S1PR2

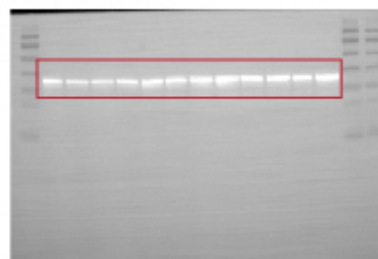

GAPDH

CCI-Chronic

(1) RhoA

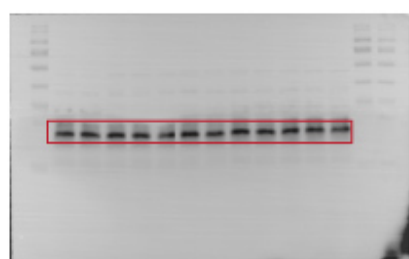

RhoA

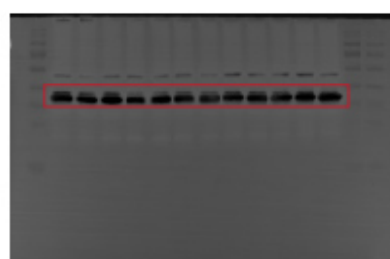

GAPDH

(2) S1PR2

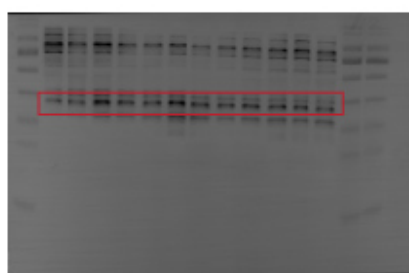

S1PR2

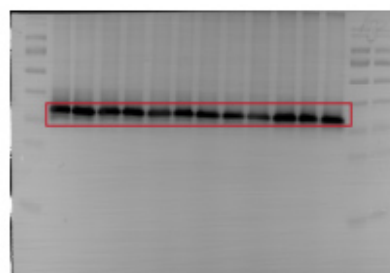

GAPDH

Supplement: Figure 8—figure supplement 1—source data 1. [file elife-99862-fig8-figsupp1-data1.pdf]

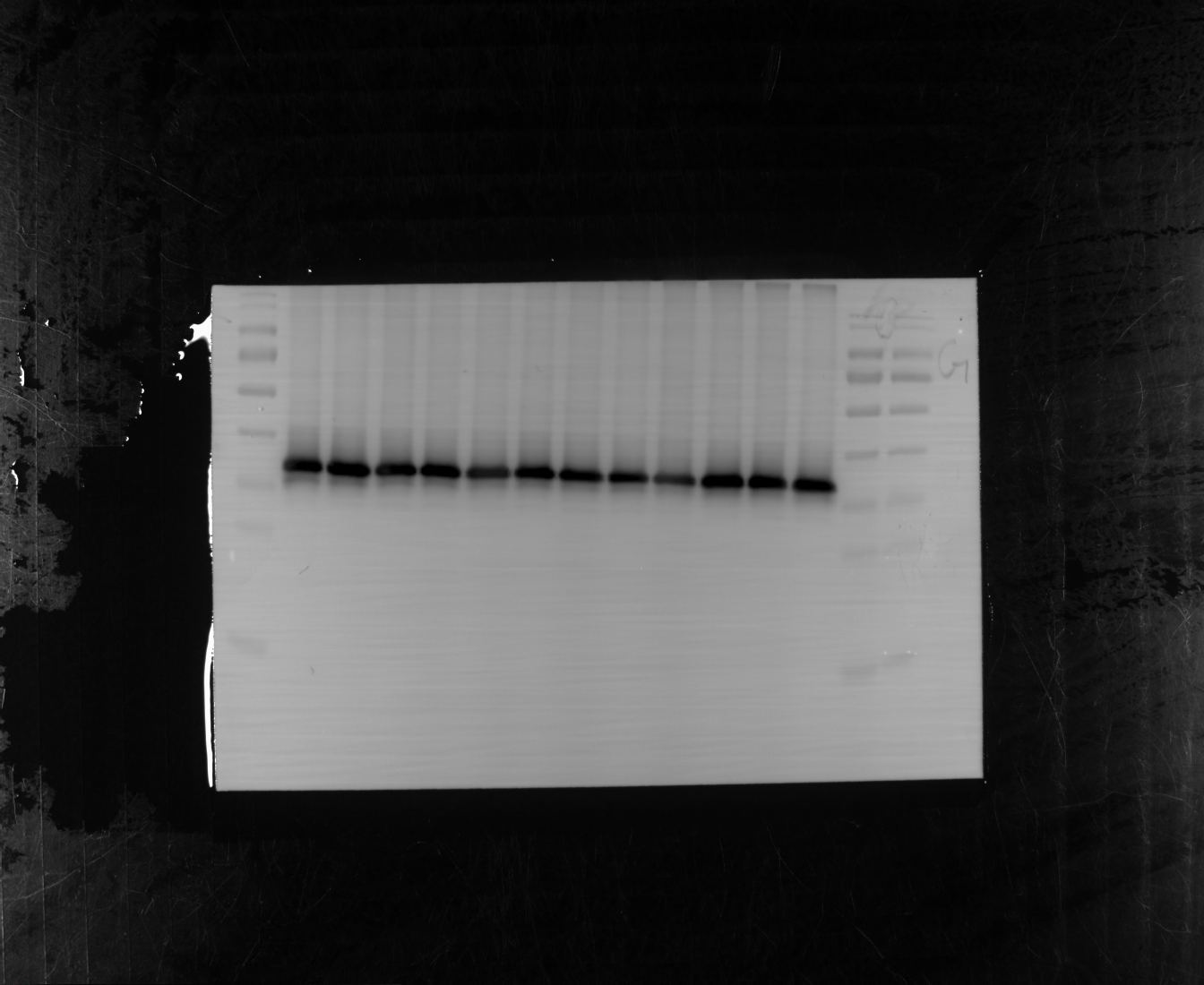

Supplement: Figure 8—figure supplement 1—source data 2. [file elife-99862-fig8-figsupp1-data2.zip › Figure 8-Figure Supplement 1-source data 2/CCI-Chronic/S1PR2/GAPDH4-M.Tif]

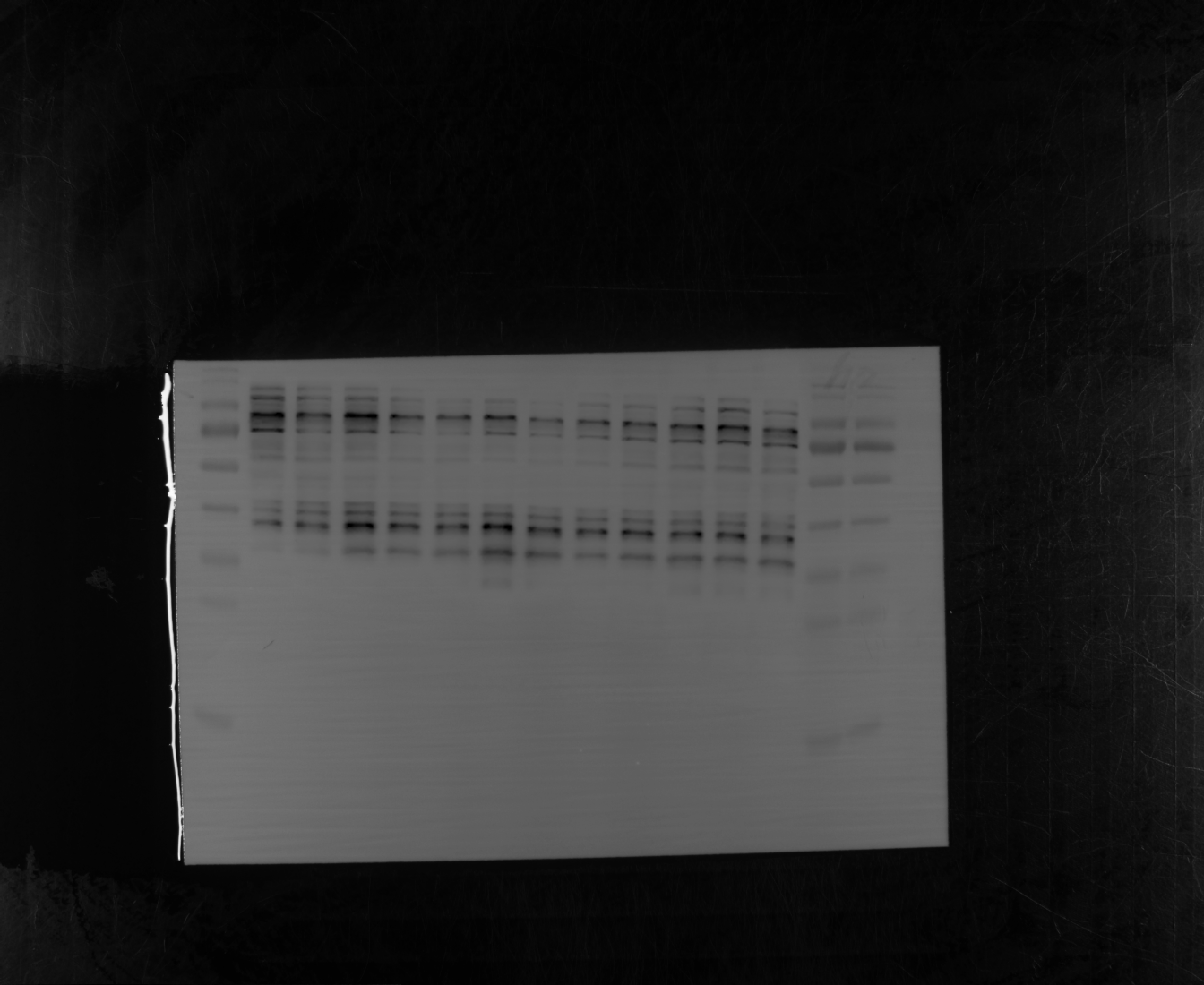

Supplement: Figure 8—figure supplement 1—source data 2. [file elife-99862-fig8-figsupp1-data2.zip › Figure 8-Figure Supplement 1-source data 2/CCI-Chronic/S1PR2/s1pr2_M.Tif]

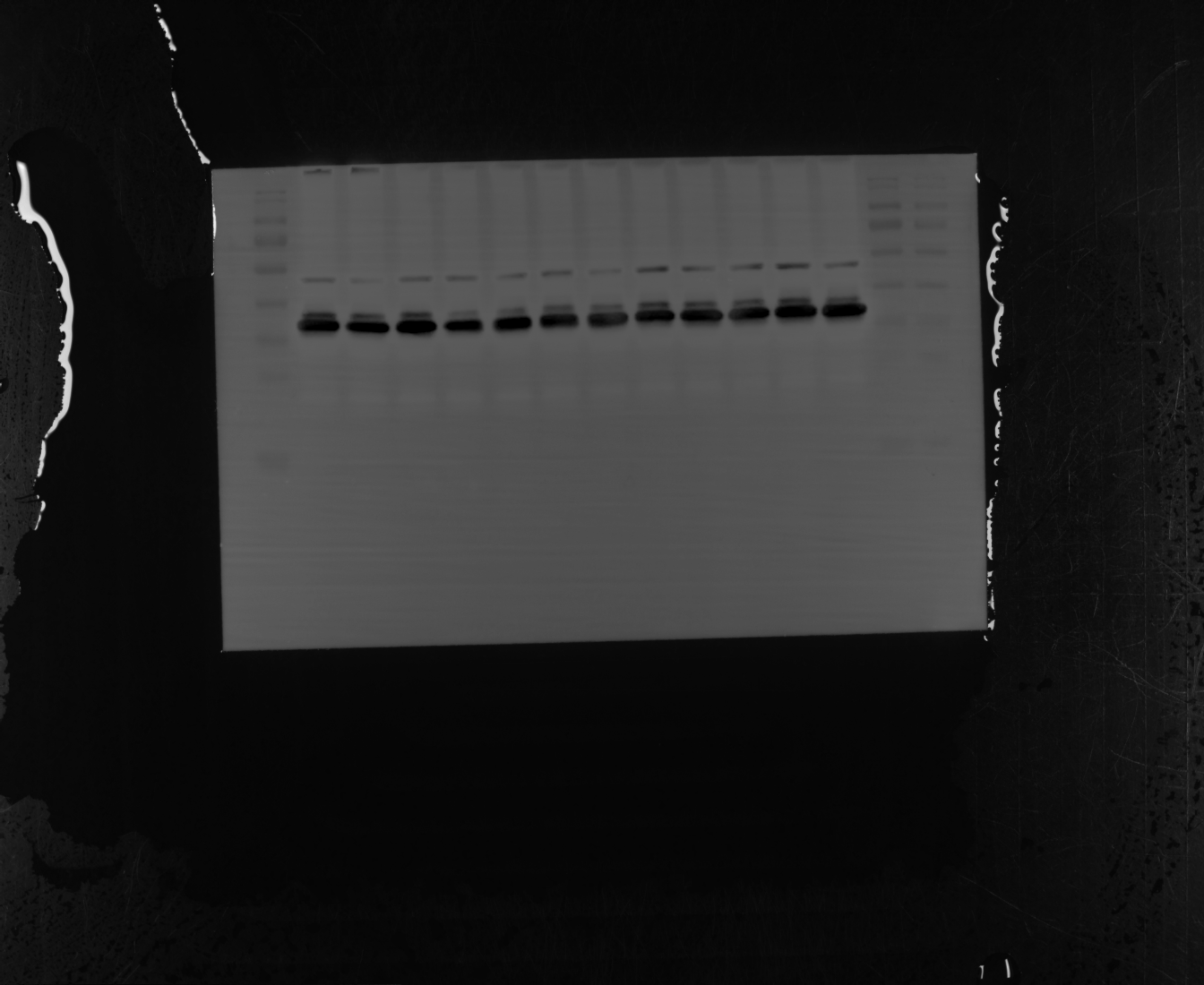

Supplement: Figure 8—figure supplement 1—source data 2. [file elife-99862-fig8-figsupp1-data2.zip › Figure 8-Figure Supplement 1-source data 2/CCI-Chronic/RhoA/gapdh-rhoa-m.Tif]

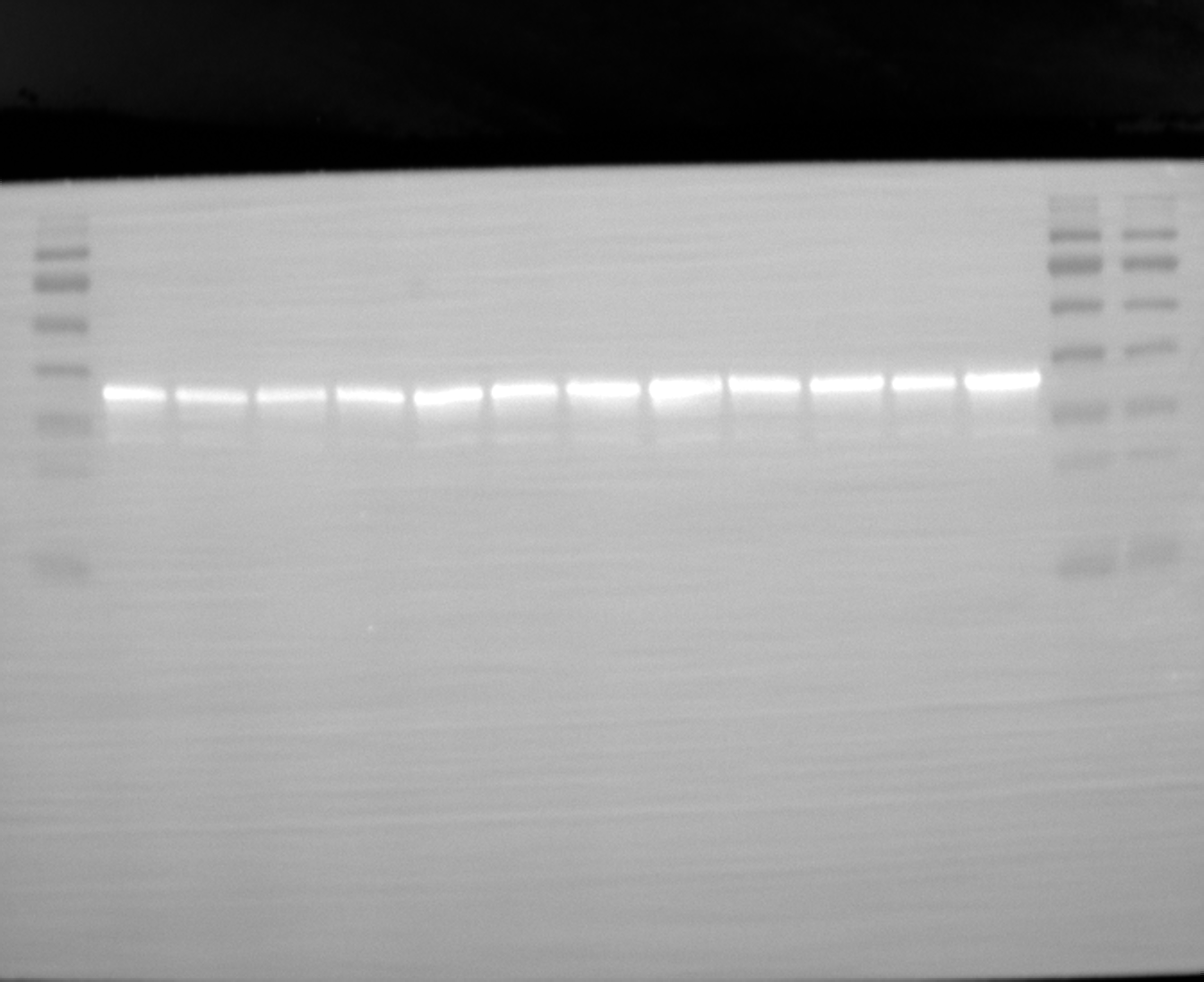

Supplement: Figure 8—figure supplement 1—source data 2. [file elife-99862-fig8-figsupp1-data2.zip › Figure 8-Figure Supplement 1-source data 2/CCI-Acute/S1PR2/gapdh-m.Tif]

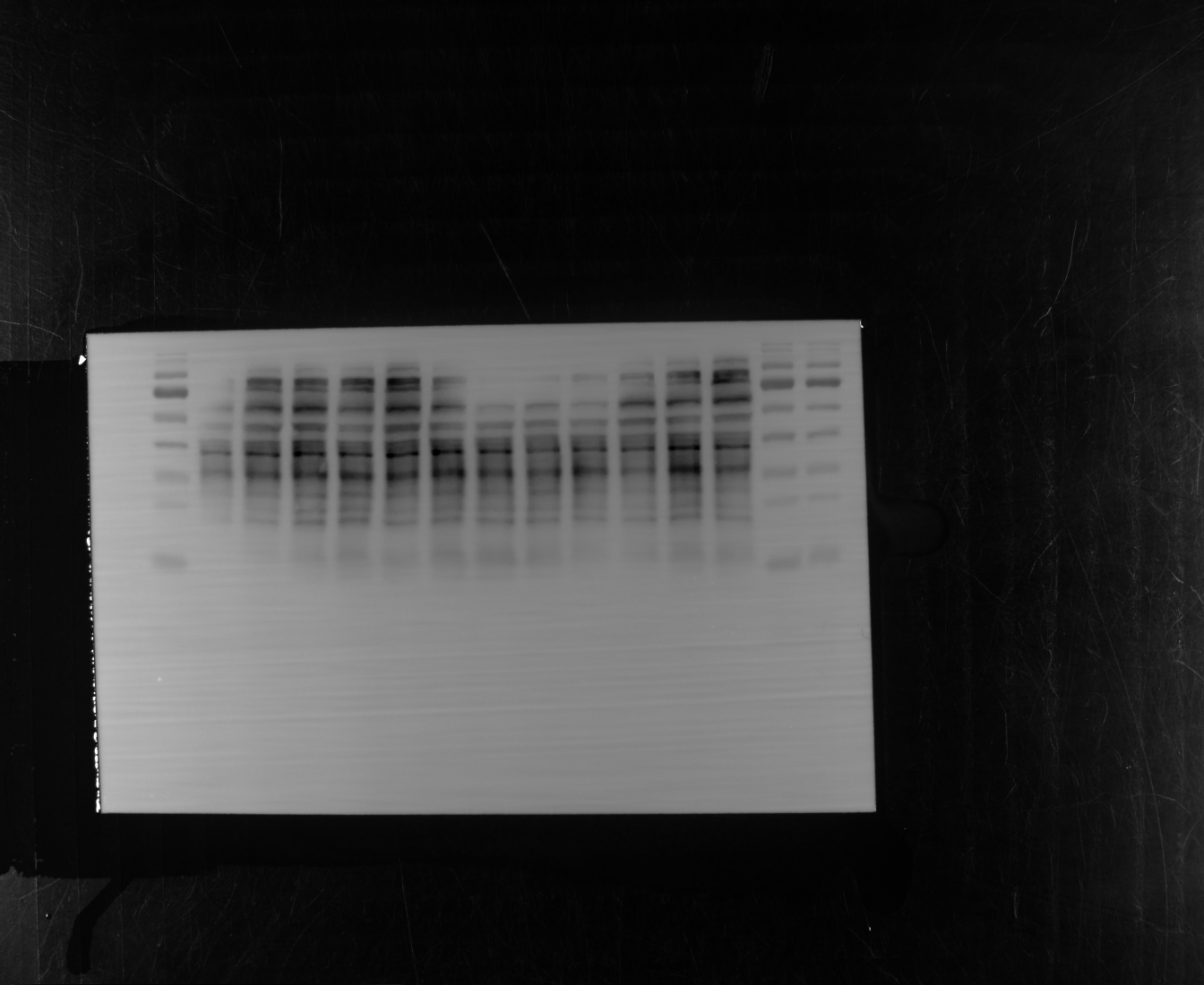

Supplement: Figure 8—figure supplement 1—source data 2. [file elife-99862-fig8-figsupp1-data2.zip › Figure 8-Figure Supplement 1-source data 2/CCI-Acute/S1PR2/s1pr2-m.Tif]

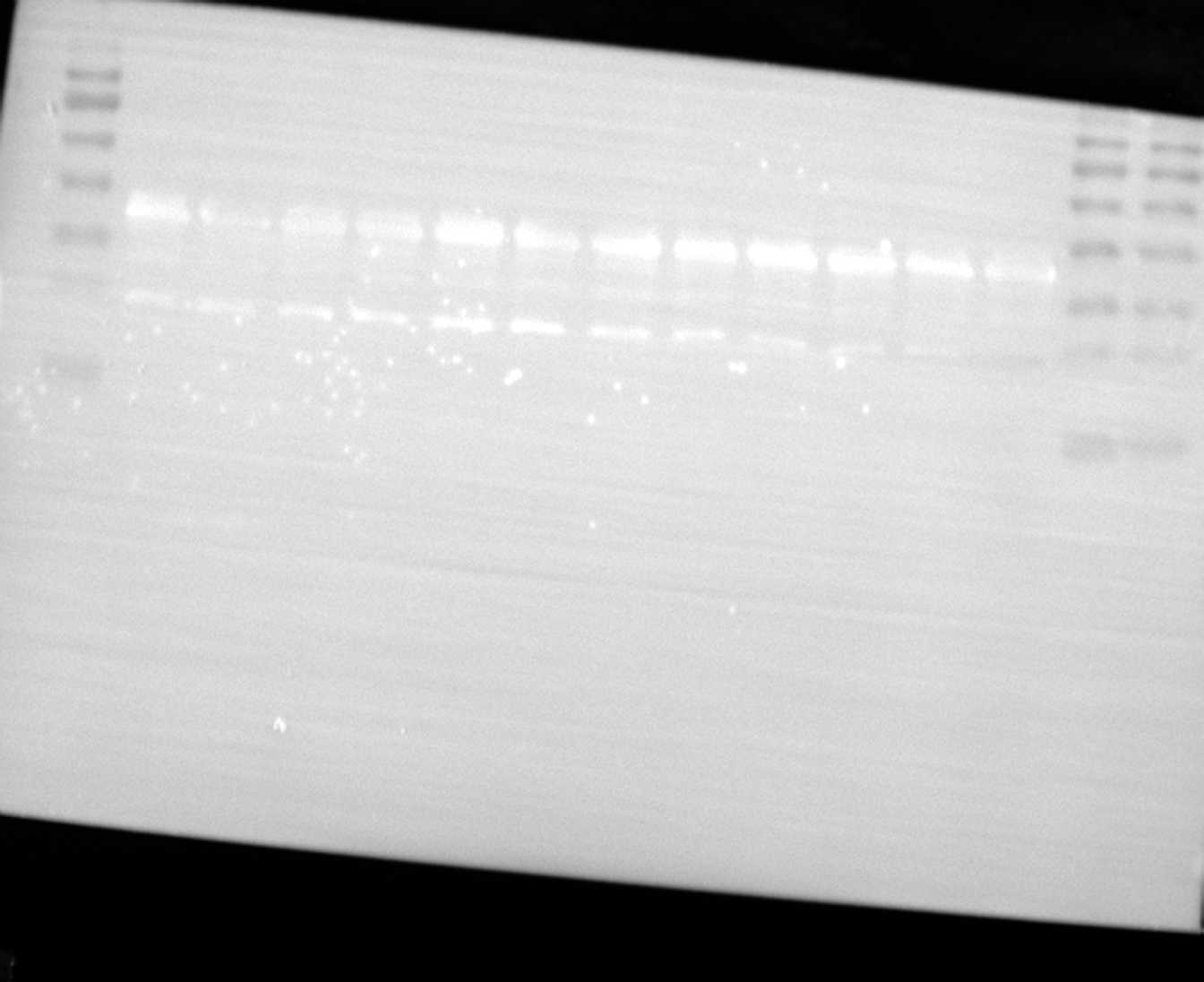

Supplement: Figure 8—figure supplement 1—source data 2. [file elife-99862-fig8-figsupp1-data2.zip › Figure 8-Figure Supplement 1-source data 2/CCI-Acute/RhoA/GAPDH-M.Tif]

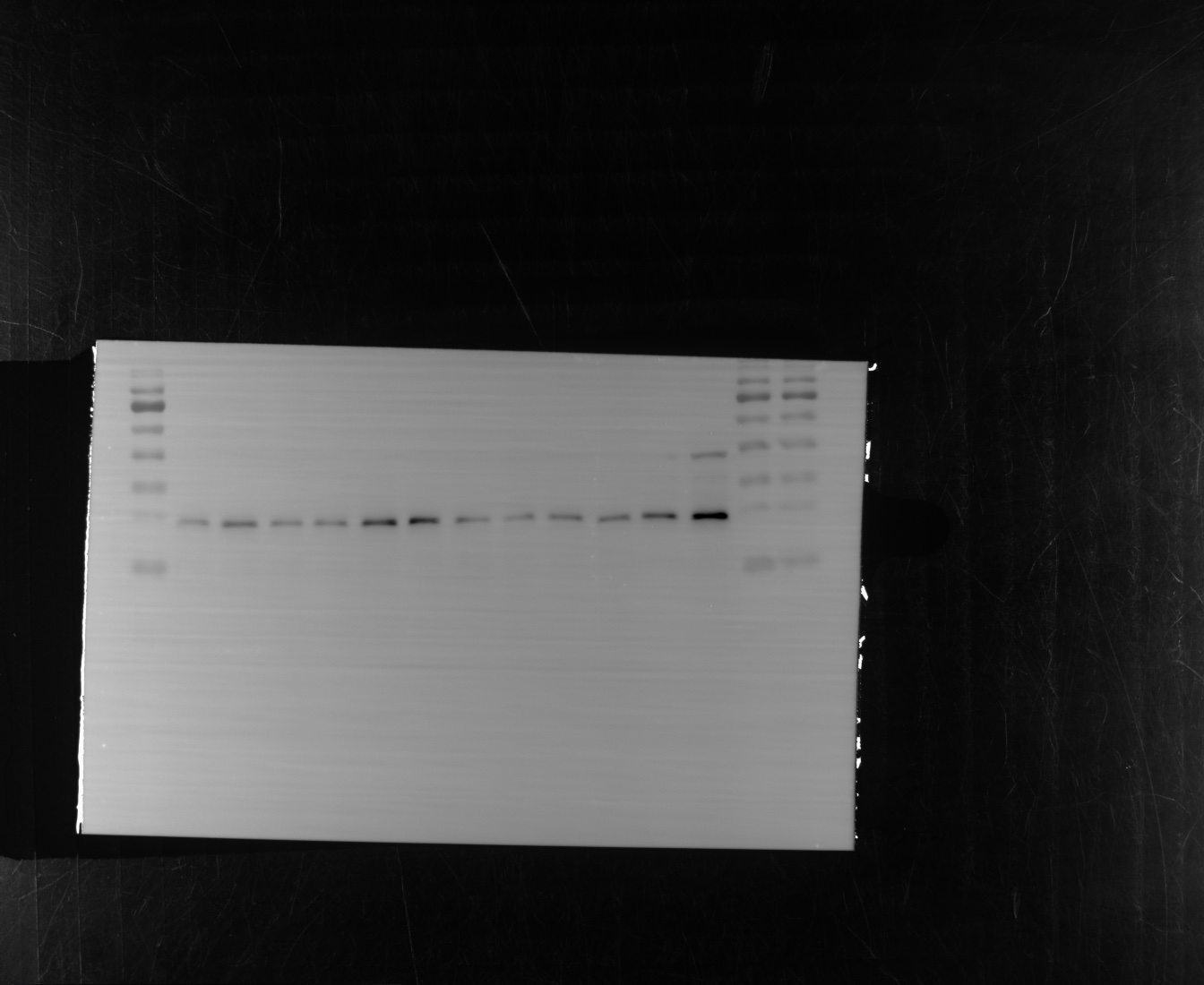

Supplement: Figure 8—figure supplement 1—source data 2. [file elife-99862-fig8-figsupp1-data2.zip › Figure 8-Figure Supplement 1-source data 2/CCI-Acute/RhoA/rhoa-m.jpg]

Full unedited blot for Figure 9

Figure 9A

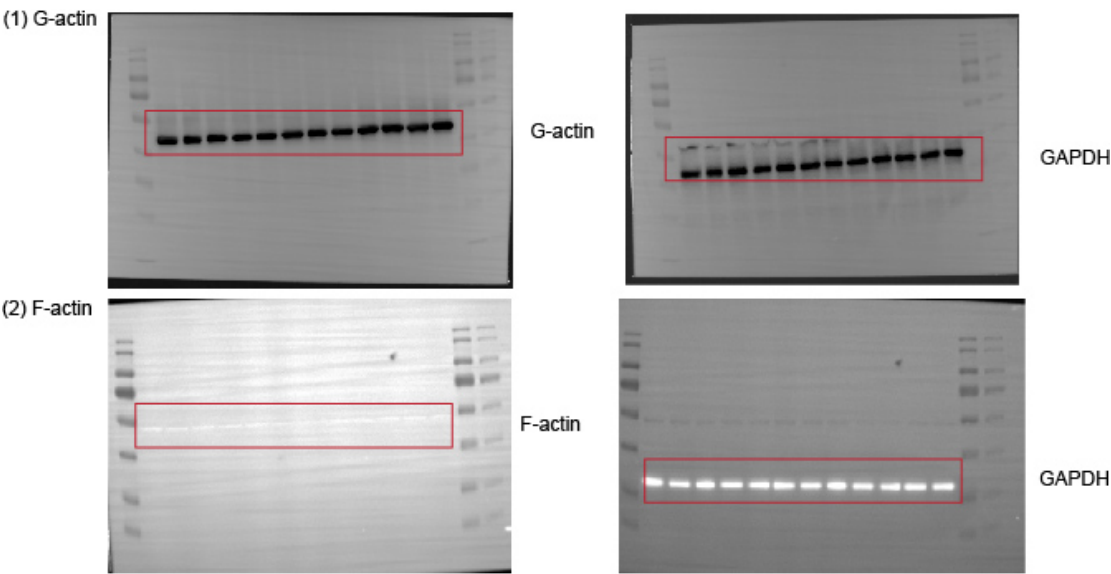

Figure 9E

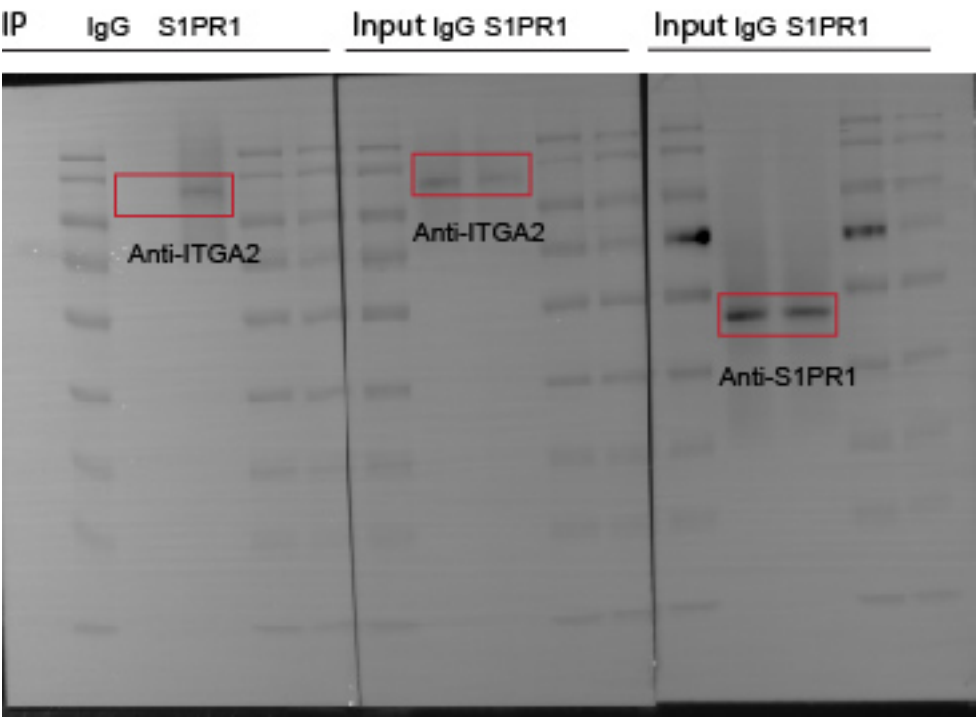

Figure 9H

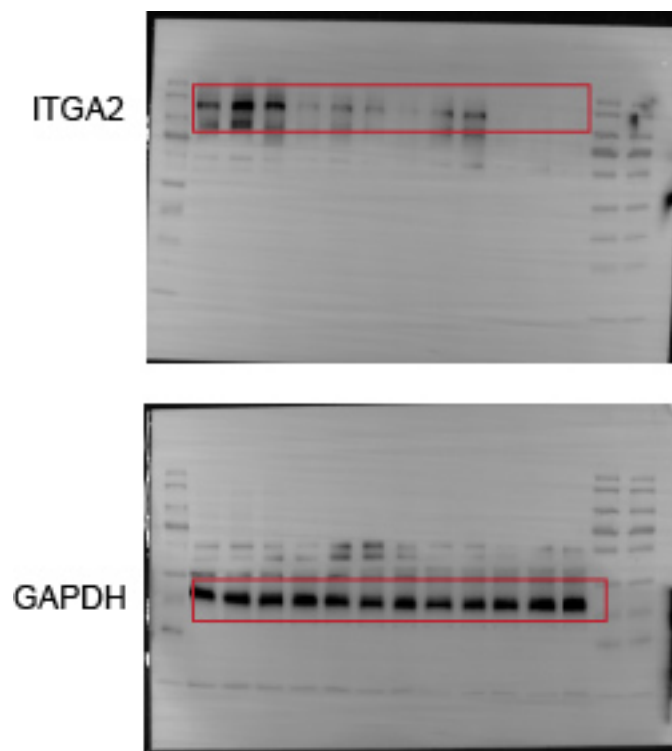

Supplement: Figure 9—source data 1. [file elife-99862-fig9-data1.pdf]

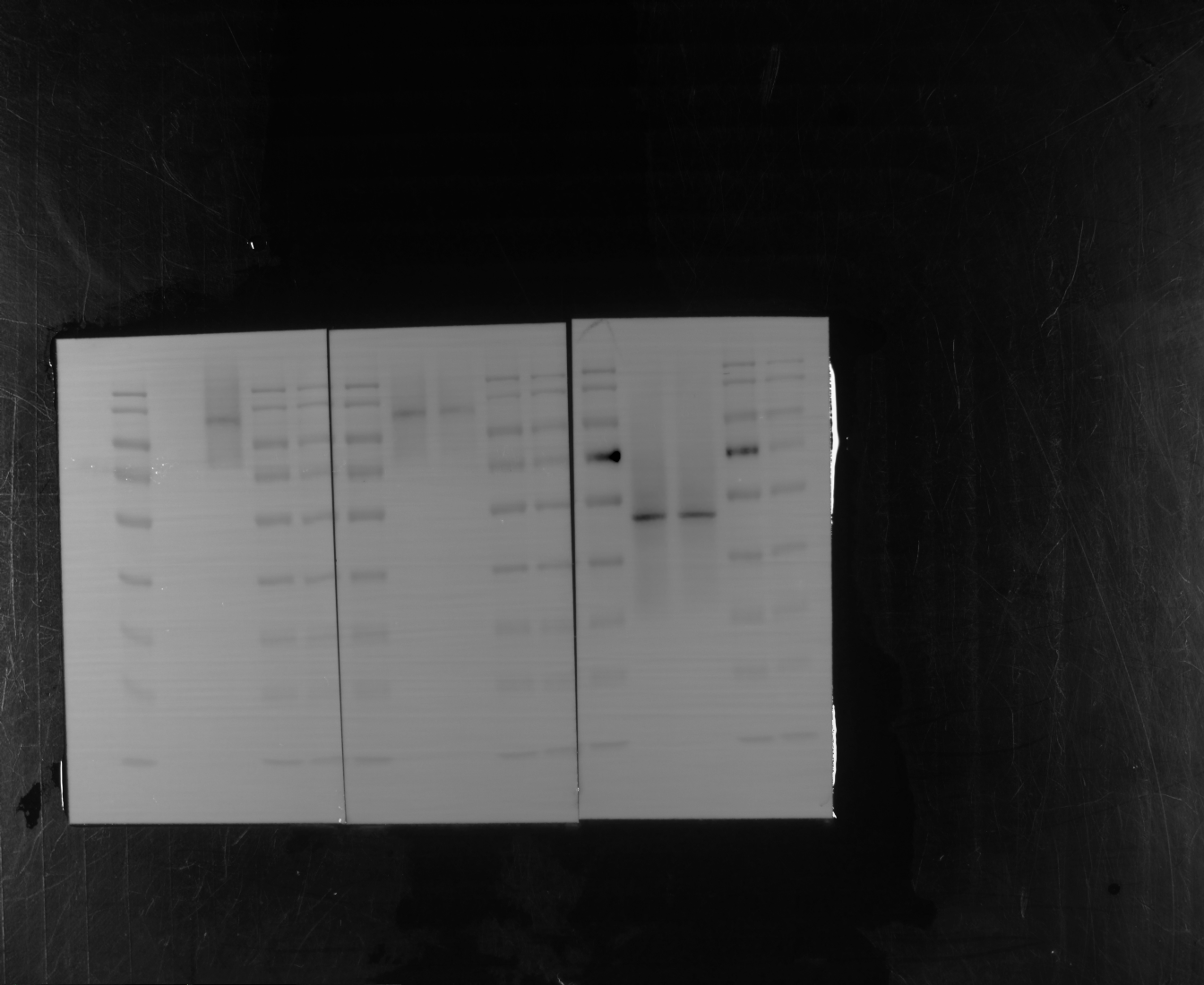

Supplement: Figure 9—source data 2. [file elife-99862-fig9-data2.zip › Figure 9-source data 2/Figure 9E/co-ip.Tif]

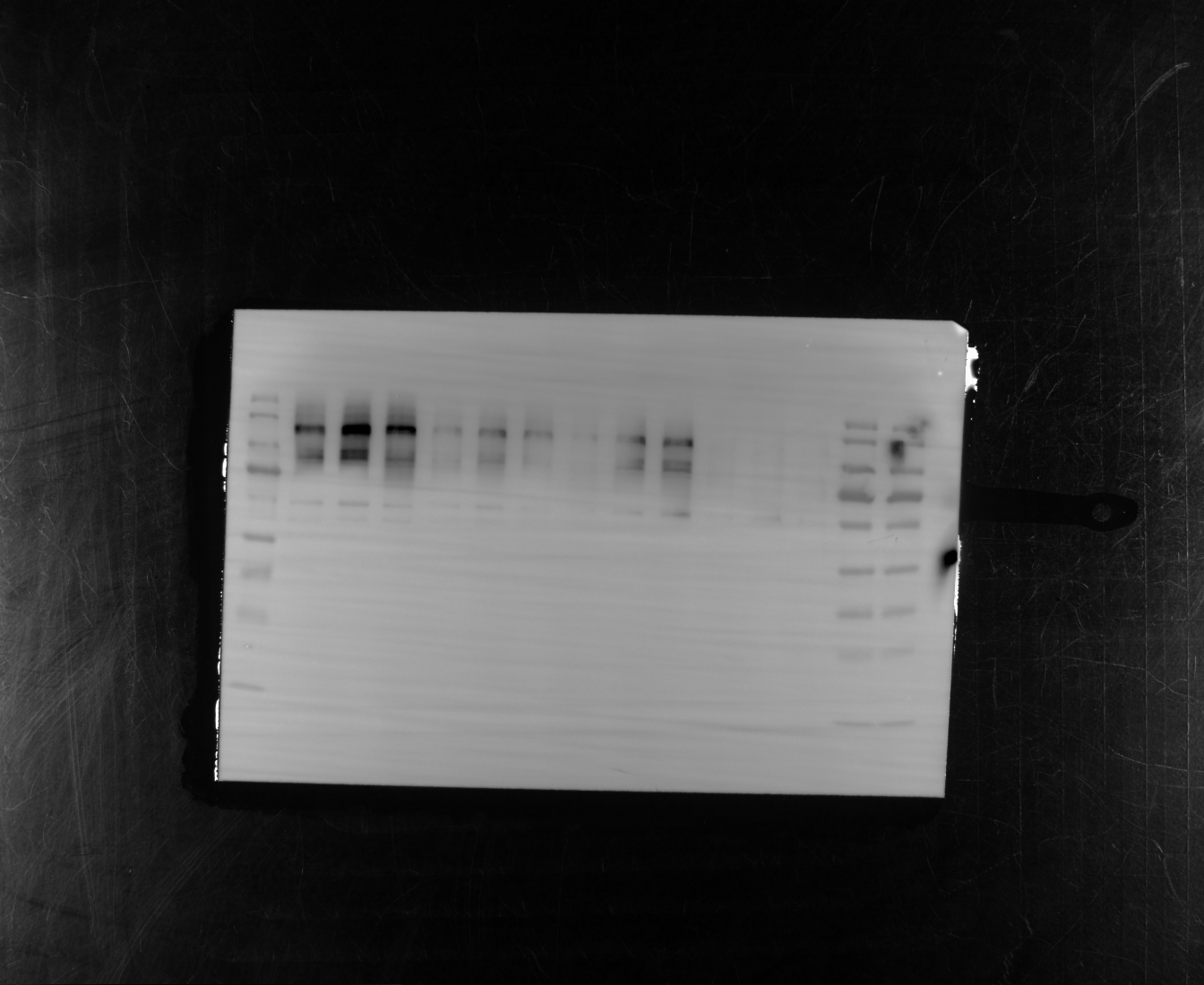

Supplement: Figure 9—source data 2. [file elife-99862-fig9-data2.zip › Figure 9-source data 2/Figure 9H/itga2 M.Tif]

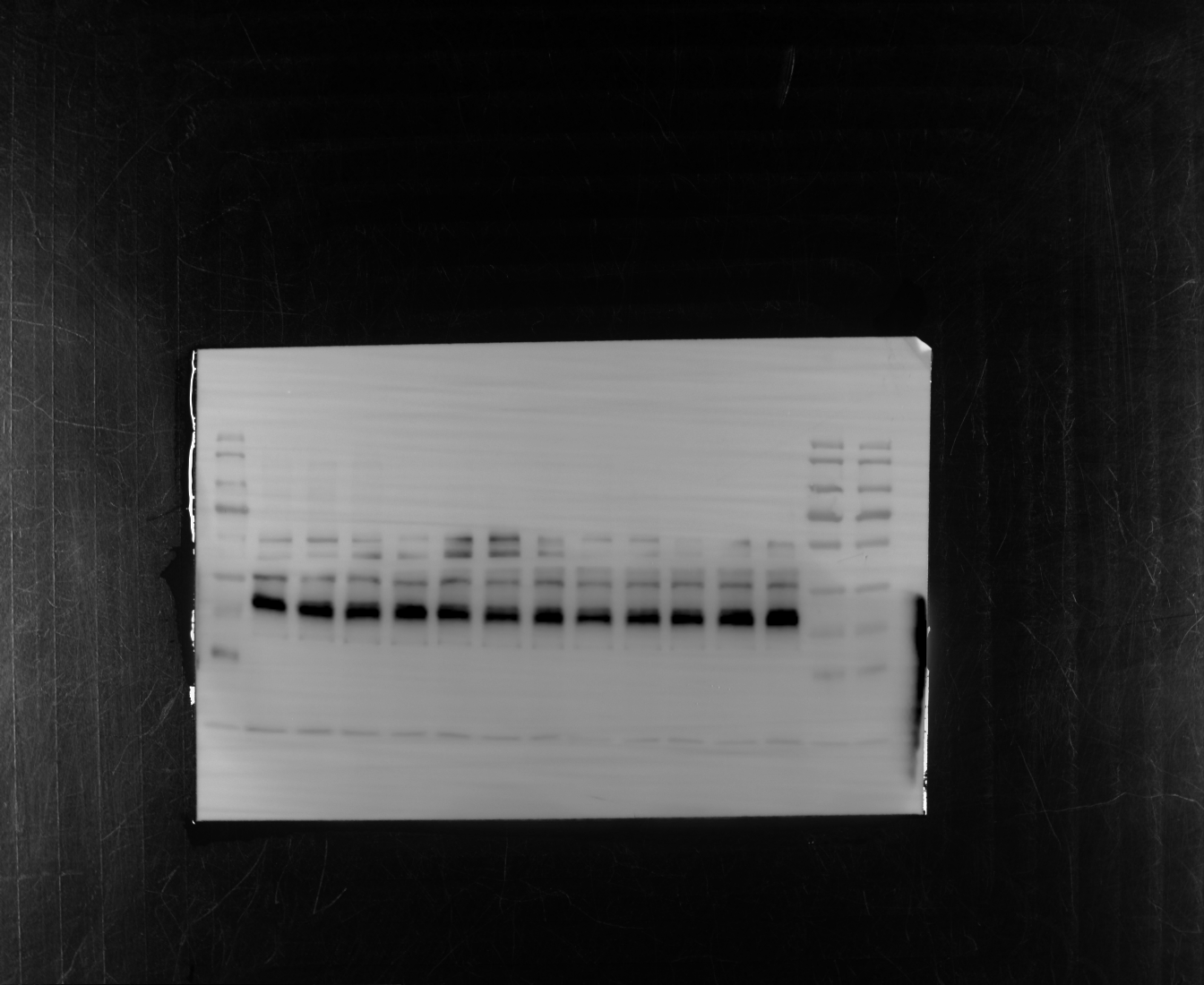

Supplement: Figure 9—source data 2. [file elife-99862-fig9-data2.zip › Figure 9-source data 2/Figure 9H/GAPDH M.Tif]

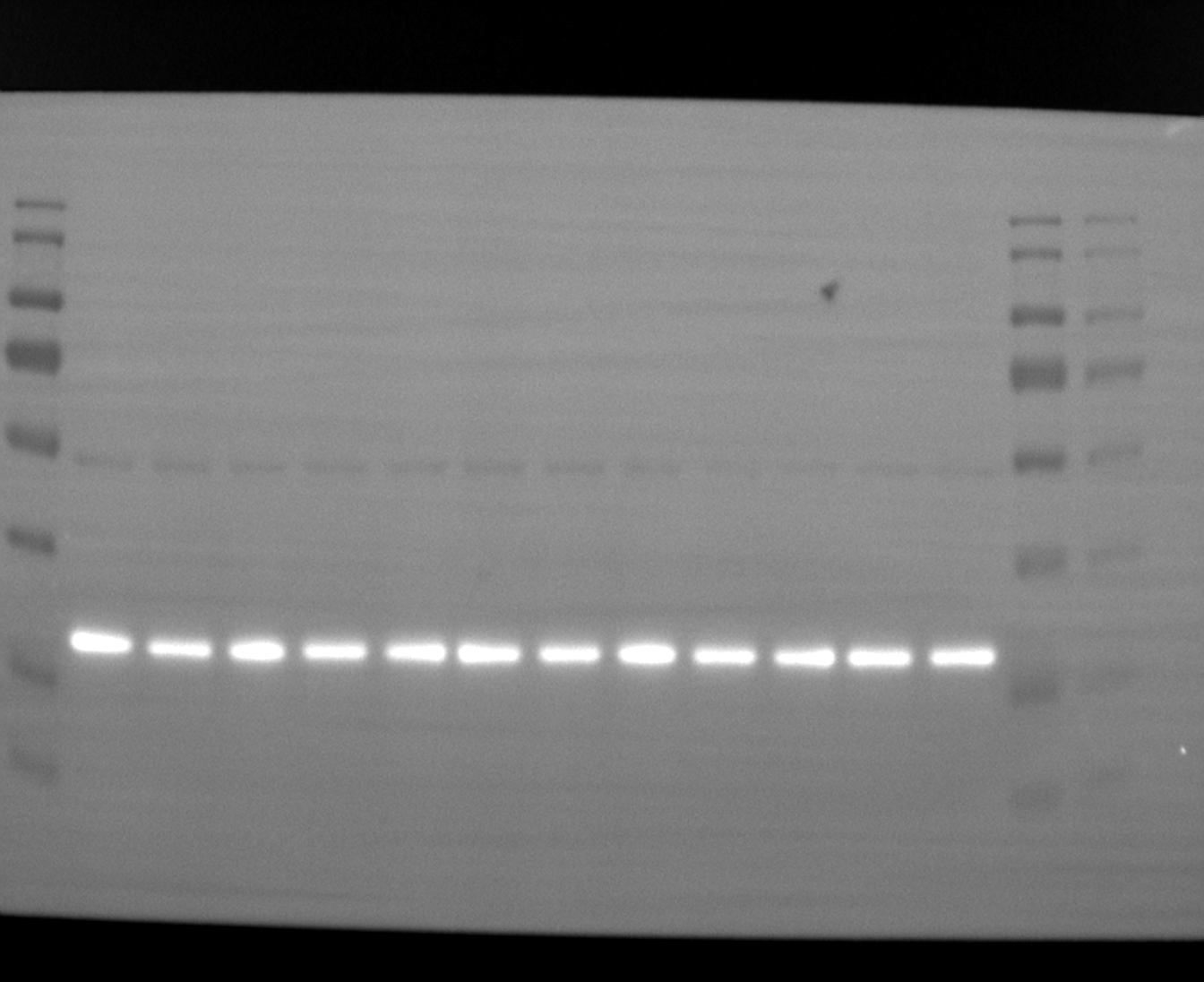

Supplement: Figure 9—source data 2. [file elife-99862-fig9-data2.zip › Figure 9-source data 2/Figure 9A/F actin/gapdh m.Tif]

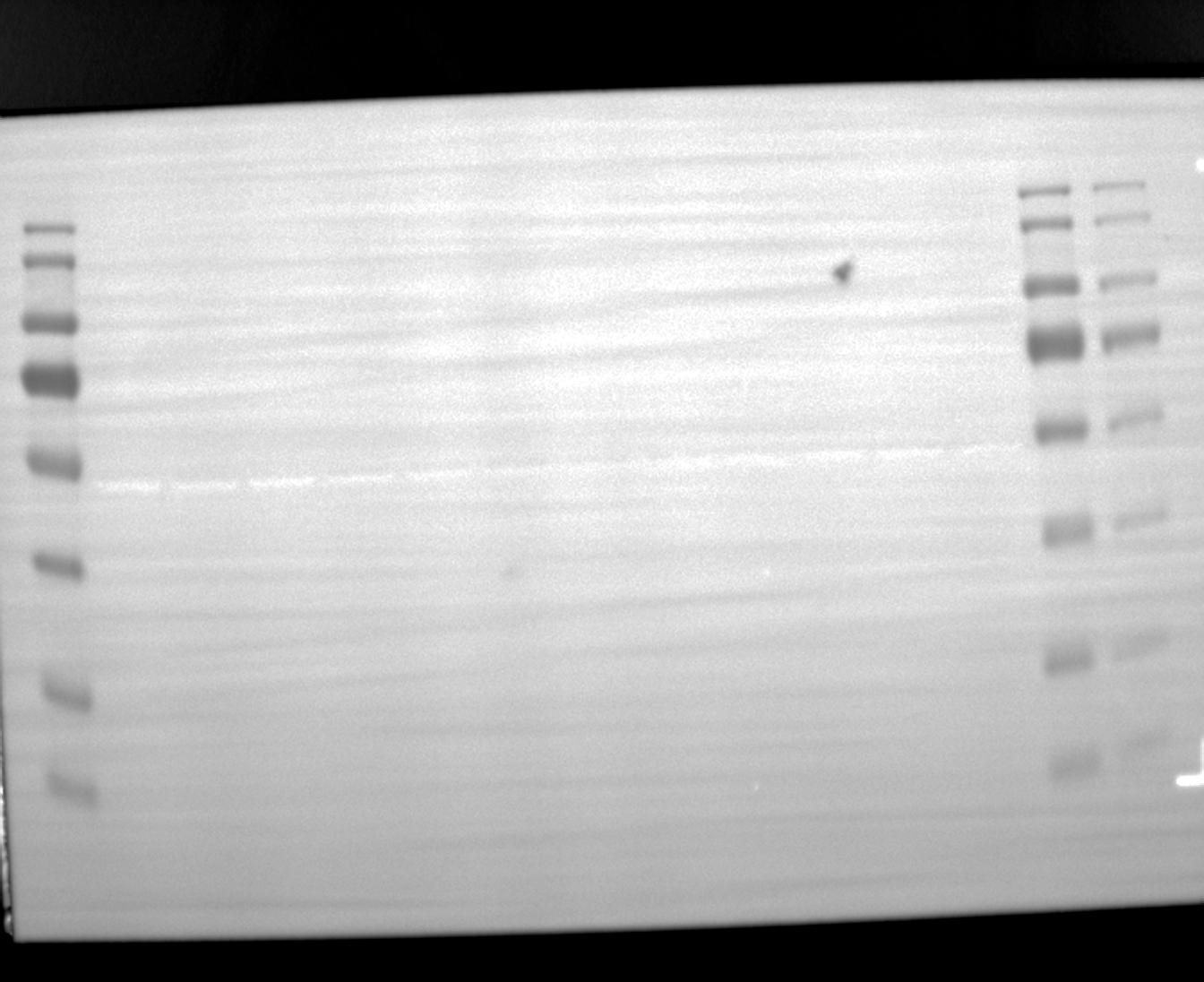

Supplement: Figure 9—source data 2. [file elife-99862-fig9-data2.zip › Figure 9-source data 2/Figure 9A/F actin/f-actin m1.jpg]

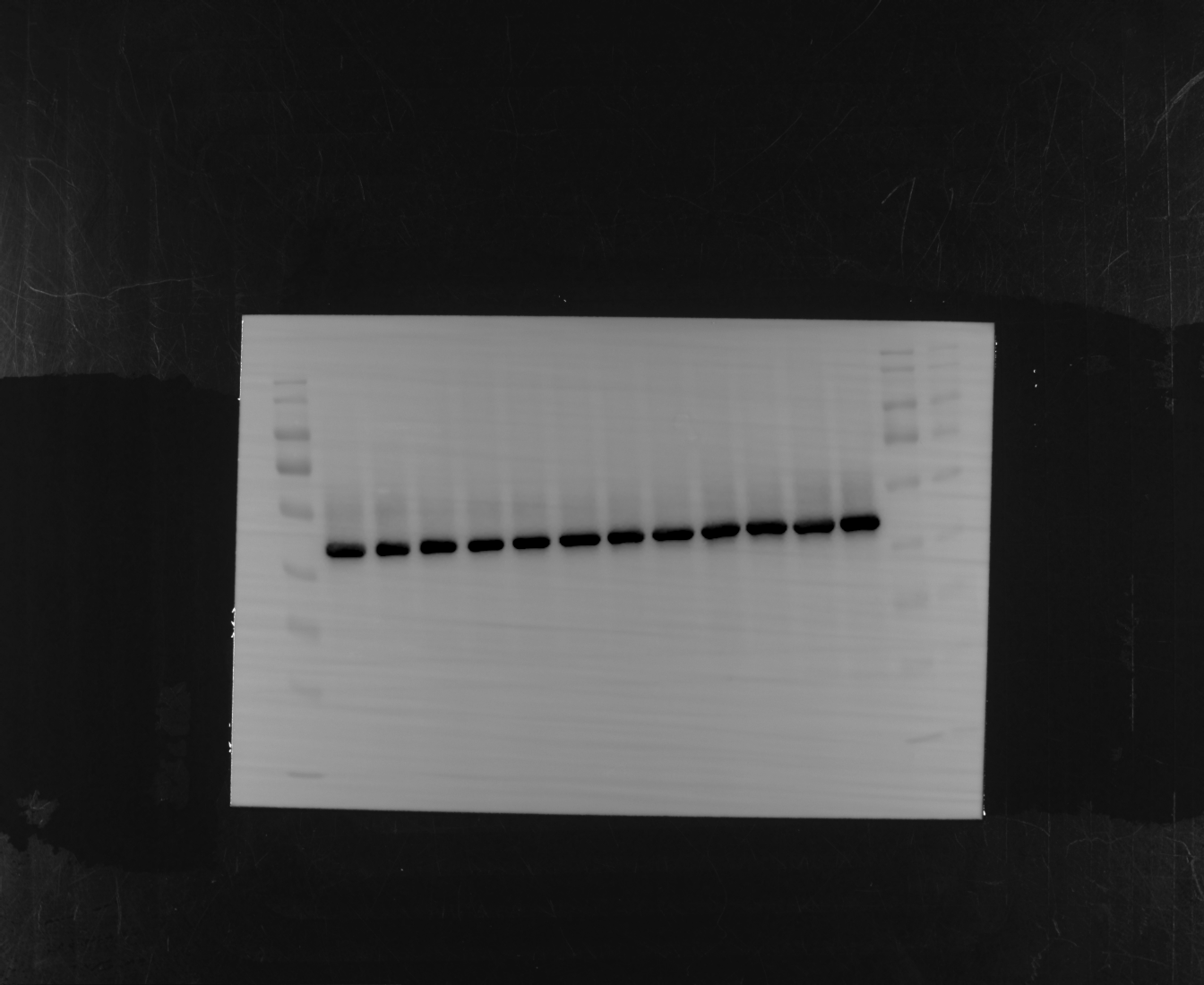

Supplement: Figure 9—source data 2. [file elife-99862-fig9-data2.zip › Figure 9-source data 2/Figure 9A/G actin/G-ACTIN1 M.Tif]

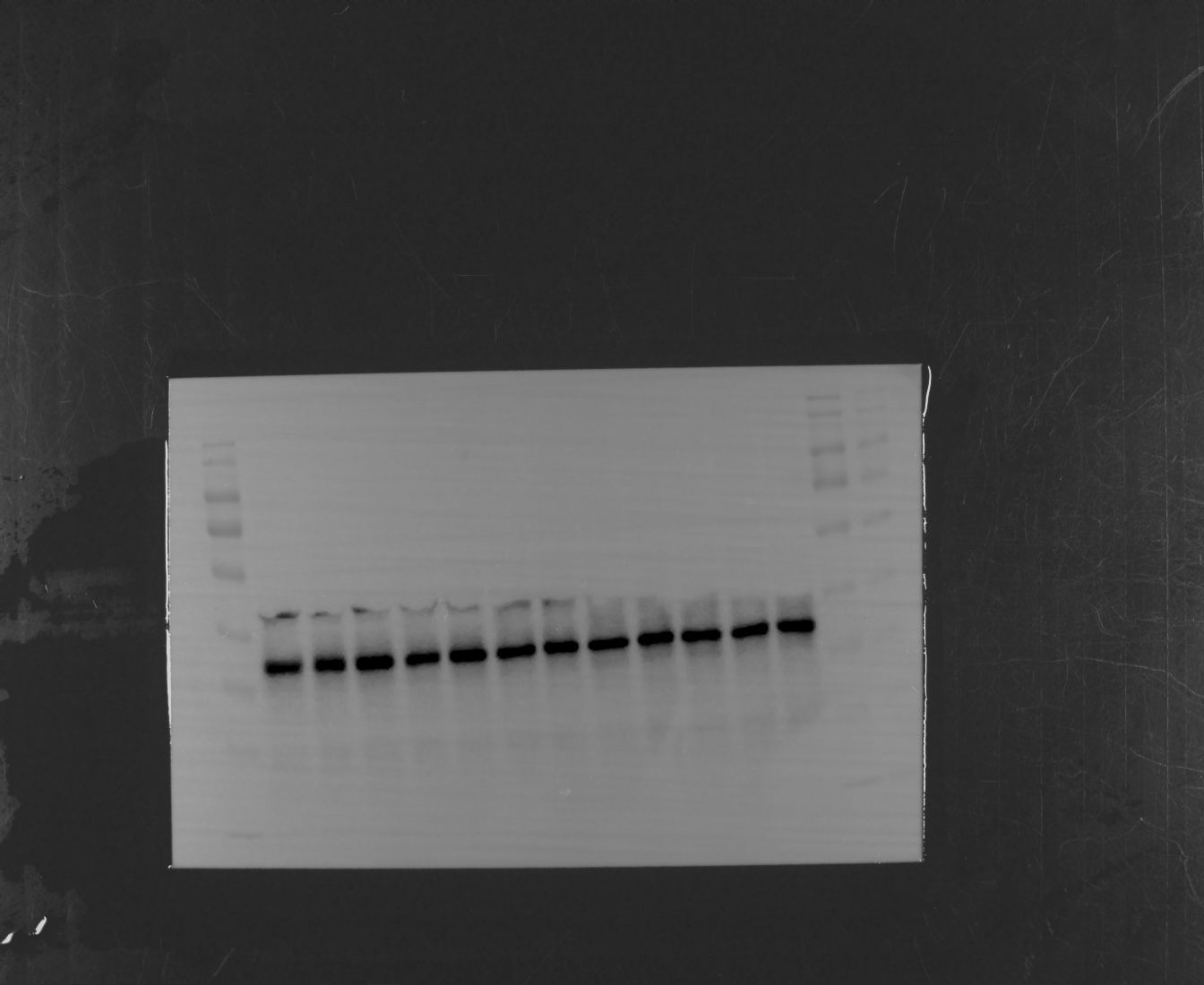

Supplement: Figure 9—source data 2. [file elife-99862-fig9-data2.zip › Figure 9-source data 2/Figure 9A/G actin/gapdh m.jpg]

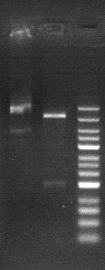

Supplement: Figure 9—figure supplement 1—source data 2. [file elife-99862-fig9-figsupp1-data2.zip › Source data-Figure 9-Figure supplement 1/Fig9-fig supplement 1A-s1pr1.jpeg]

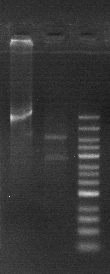

Supplement: Figure 9—figure supplement 1—source data 2. [file elife-99862-fig9-figsupp1-data2.zip › Source data-Figure 9-Figure supplement 1/Fig9-fig supplement 1B-itga2.jpeg]
