# Supplementary material for: Dysfunctional S1P/S1PR1 signaling in the dentate gyrus drives vulnerability of chronic pain-related memory impairment
Source: eLife. 2024 Dec 19;13:RP99862. doi: 10.7554/eLife.99862 (PMC11658773; doi:10.7554/eLife.99862)
Supplement: Figure 8—source data 1. [file elife-99862-fig8-data1.pdf]

Full unedited blot for Figure 8C

(1) RAC1

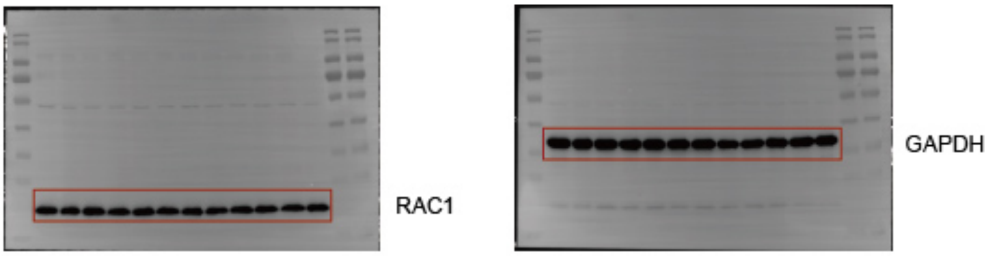

(2) CDC42

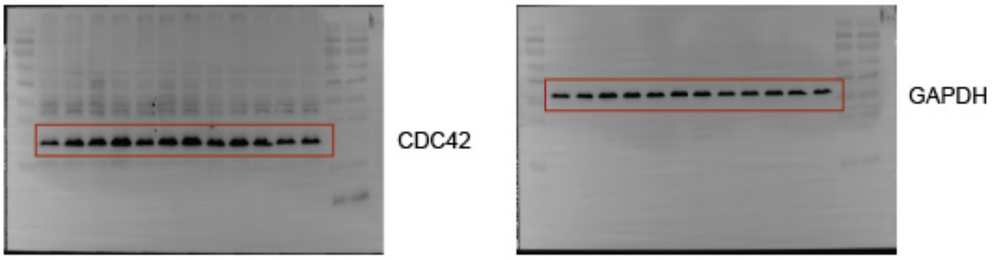

(3) ARP2

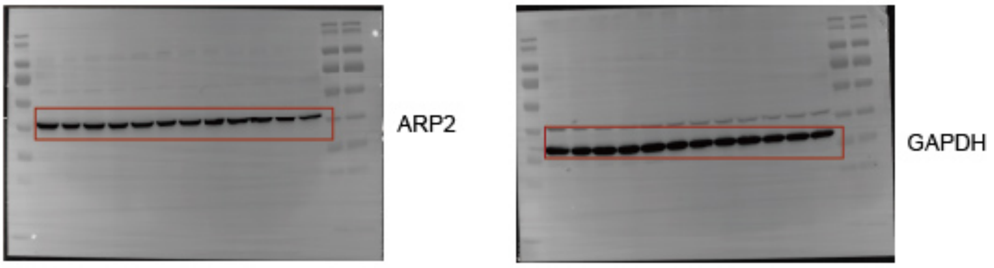

(4) ARP3

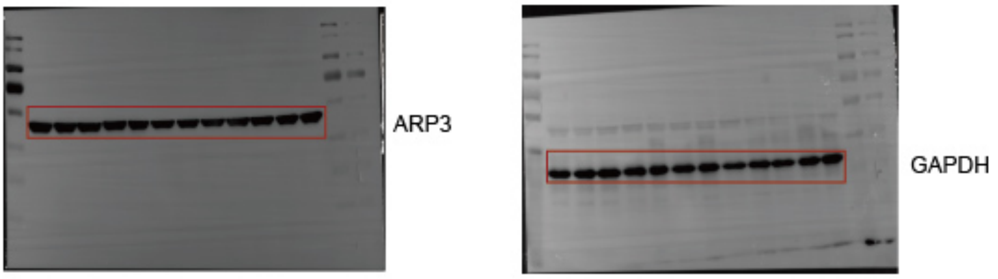

(5) ITGA2

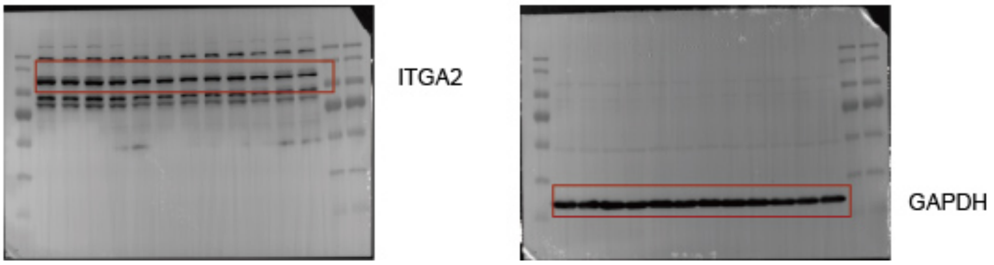

Full unedited blot for Figure 8E

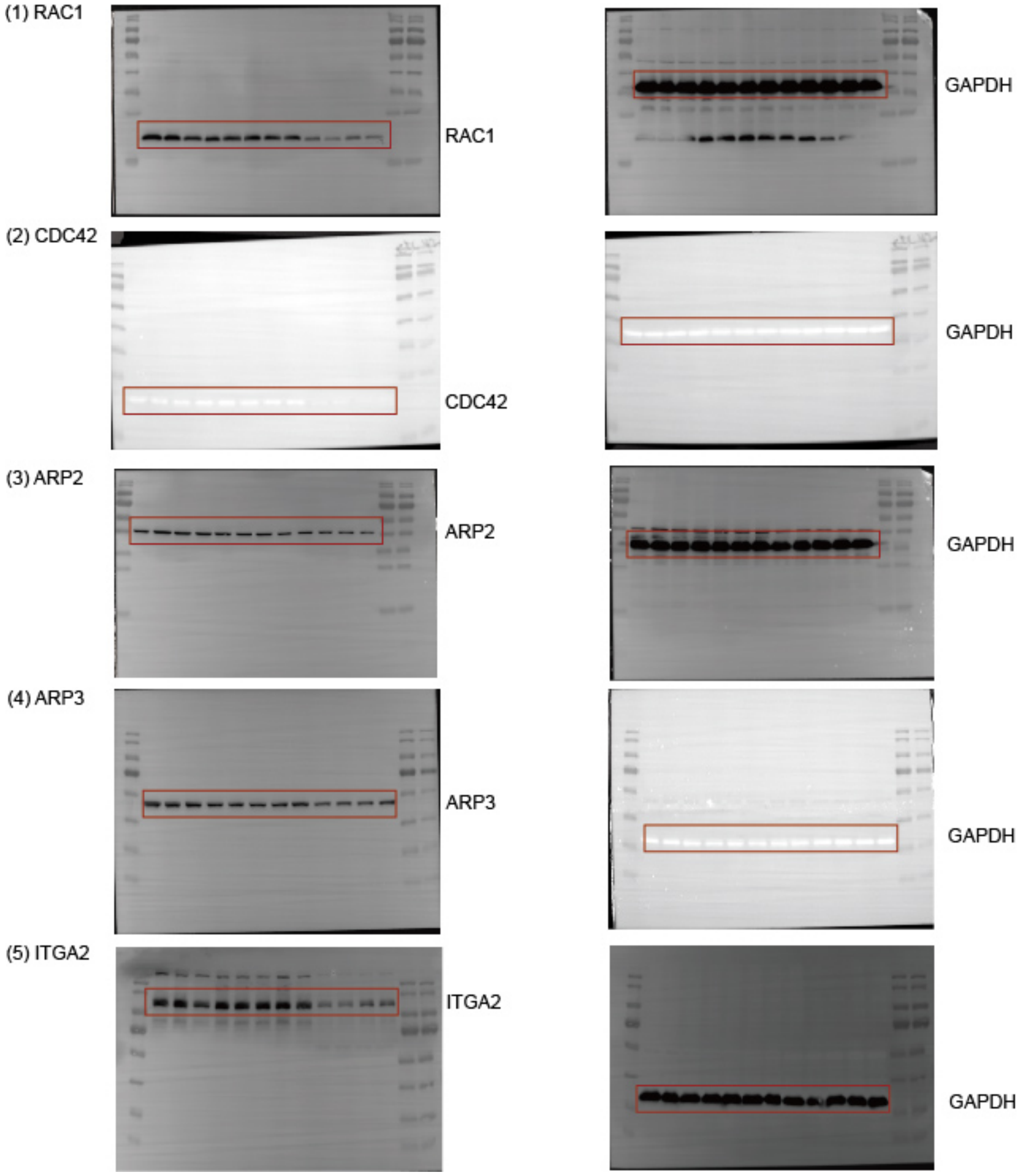

Full unedited blot for Figure 8G

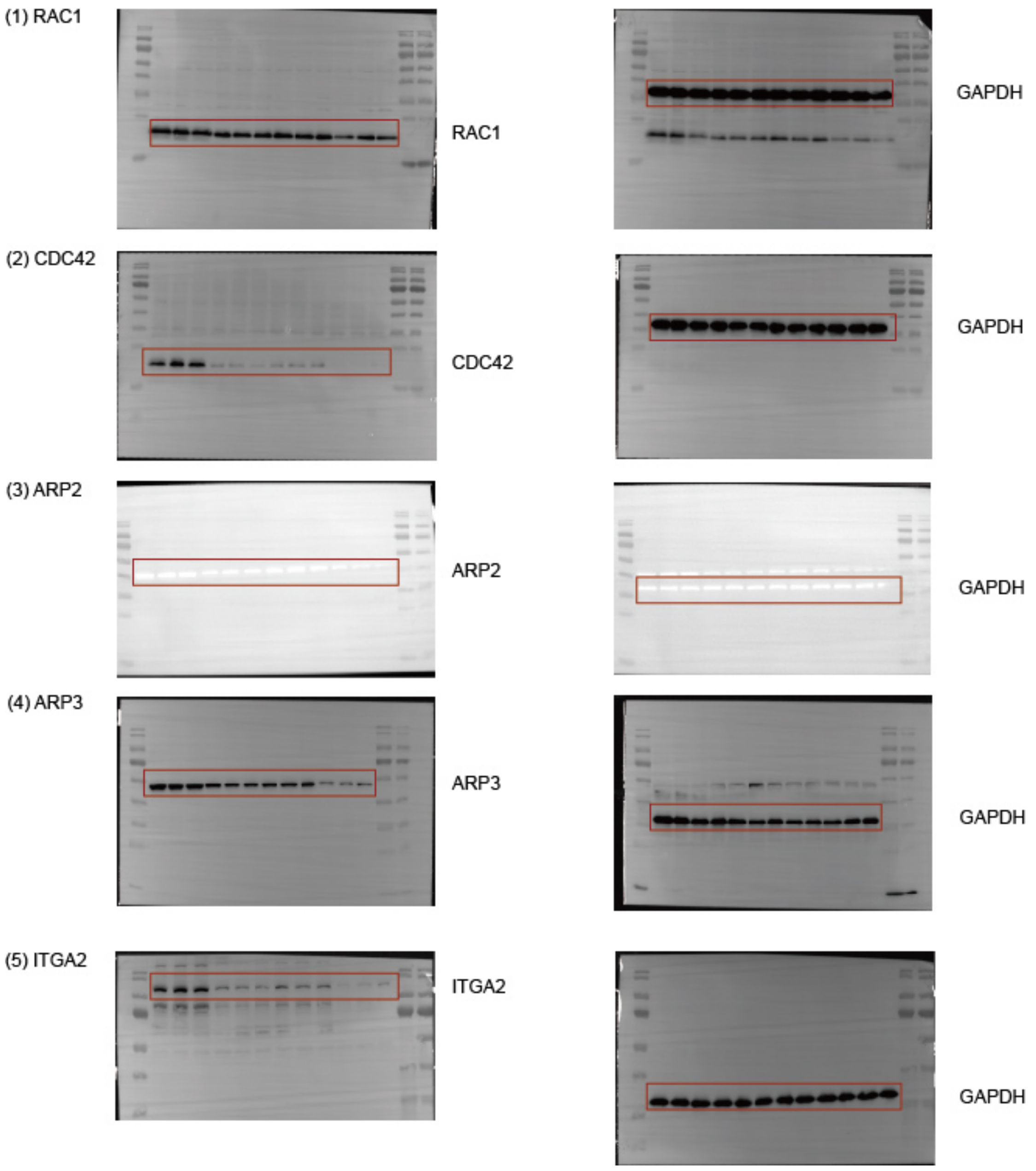

Full unedited blot for Figure 8I

(1) RAC1

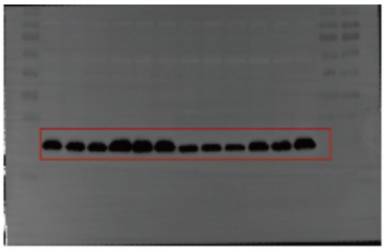

RAC1

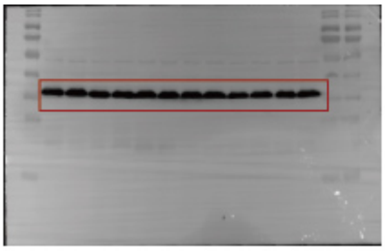

GAPDH

(2) CDC42

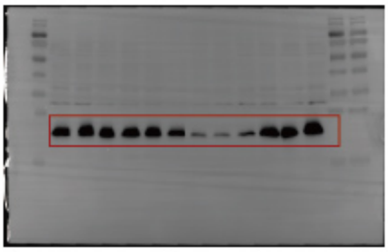

CDC42

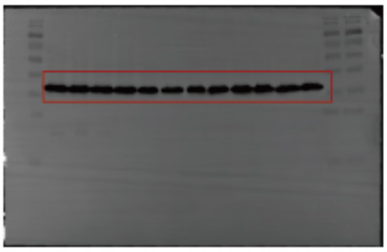

GAPDH

(3) ARP2

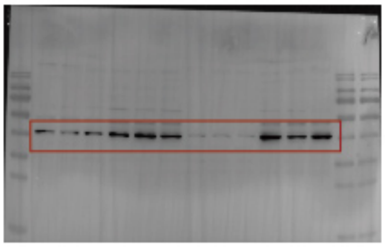

ARP2

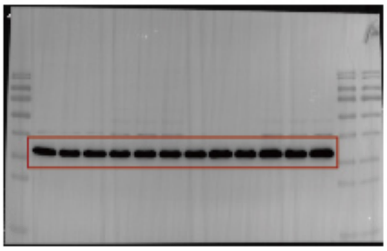

GAPDH

(4) ARP3

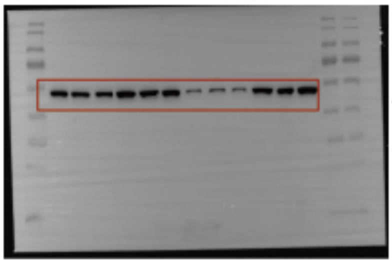

ARP3

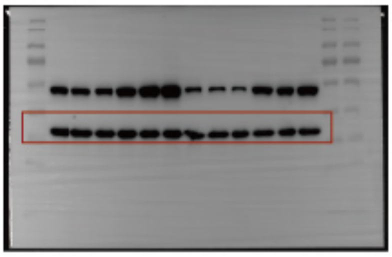

GAPDH

(5) ITGA2

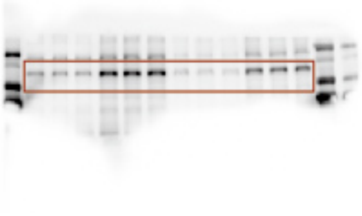

ITGA2

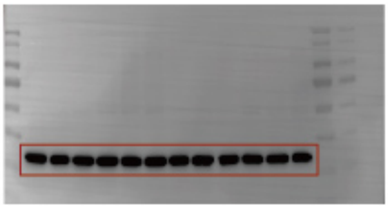

GAPDH
