# Supplementary material for: Dysfunctional S1P/S1PR1 signaling in the dentate gyrus drives vulnerability of chronic pain-related memory impairment
Source: eLife. 2024 Dec 19;13:RP99862. doi: 10.7554/eLife.99862 (PMC11658773; doi:10.7554/eLife.99862)
Supplement: Figure 9—figure supplement 1—source data 1. [file elife-99862-fig9-figsupp1-data1.pdf]

Unedited gel for pBT3-STE-s1pr1 plasmid digested with XbaI-HindIII.

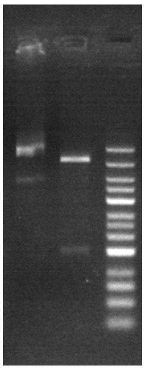

Lane 1: empty plasmid control.

Lane 2: plasmid digested with restriction enzymes

Lane 3: DNA ladder

Unedited gel for pPR3-C-itga2 plasmid digested with SalI-BamHI.

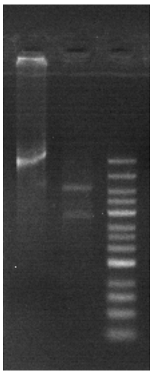

Lane 1: empty plasmid control.

Lane 2: plasmid digested with restriction enzymes

Lane 3: DNA ladder
