# Supplementary material for: Dysfunctional S1P/S1PR1 signaling in the dentate gyrus drives vulnerability of chronic pain-related memory impairment
Source: eLife. 2024 Dec 19;13:RP99862. doi: 10.7554/eLife.99862 (PMC11658773; doi:10.7554/eLife.99862)
Supplement: Supplementary file 1. [file elife-99862-supp1.docx]

**Supplementary File 1**

**Supplementary File 1a. Virus vectors.**

| **Virus vector** | **Sequence** | **Vendor** |
| --- | --- | --- |
| rAAV- CaMKIIa-EGFP-sh*S1pr1*-WPREs | GCTCTACCACAAGCACTATAT | BrainVTA, Wuhan, China |
| rAAV-CaMKIIa-*S1pr1*-P2A EGFP-WPRE-hGH polyA | NCBI: NM_007901.5 | BrainVTA, Wuhan, China |
| rAAV-CaMKIIa-mCherry-sh*Itga2*-WPREs | GACCTCACAAACACCTTCAGA | BrainVTA, Wuhan, China |

**Supplementary File 1b. Chemicals.**

| **Chemicals** | **Vendor** | **Cat #** |
| --- | --- | --- |
| Triton X-100 | Sigma-Aldrich | 9036-19-5 |
| Normal Donkey Serum | Solarbio | SL050 |
| Meloxicam | Solarbio | M9840 |
| Dimethyl suifoxide | Solarbio | D8370 |
| SEW2871 | Aladdin | 256414-75-2 |

**Supplementary File 1c. Anti-bodies.**

| **Anti-bodies** | **Vendor** | **Cat #** | **RRID** | **Dilutability** |
| --- | --- | --- | --- | --- |
| Anti-NeuN antibody [1B7] | Abcam | ab104224 | AB_10711040 | 1:500 |
| GFAP (GA5) Mouse mAb | Cell Signaling | 3670S | / | 1:500 |
| Anti-Iba1 antibody | Abcam | ab5076 | AB_2224402 | 1:500 |
| CaMKII alpha Monoclonal Antibody (6G9) | Invitrogen | MA1-048 | / | 1:500 |
| Anti-GAD67 Antibody, clone 1G10.2 | Sigma-Aldrich | MAB5406 | AB_2278725 | 1:500 |
| Donkey anti-Rabbit IgG (H+L) Highly Cross-Adsorbed Secondary Antibody, Alexa Fluor™ 488 | Thermo Fisher Scientific | **A-21206** | AB_2535792 | 1:500 |
| Donkey anti-Rabbit IgG (H+L) Highly Cross-Adsorbed Secondary Antibody, Alexa Fluor™ 594 | Thermo Fisher Scientific | A-21207 | AB_141637 | 1:500 |
| Donkey anti-Mouse IgG (H+L) Highly Cross-Adsorbed Secondary Antibody, Alexa Fluor™ 488 | Thermo Fisher Scientific | A21202 | AB_141607 | 1:500 |
| Donkey anti-Mouse IgG (H+L) Highly Cross-Adsorbed Secondary Antibody, Alexa Fluor™ 594 | Thermo Fisher Scientific | **A-21203** | AB_2535789 | 1:500 |
| Donkey anti-Goat IgG (H+L) Cross-Adsorbed Secondary Antibody, Alexa Fluor™ 488 | Thermo Fisher Scientific | A-11055 | AB_2534102 | 1:500 |
| Rac1 Polyclonal antibody | Proteintech | 24072-1-AP | AB_2879427 | 1:1000 |
| CDC42 Polyclonal antibody | Proteintech | 10155-1-AP | AB_2078096 | 1:1000 |
| ARP2 Polyclonal antibody | Proteintech | 10922-1-AP | AB_2221854 | 1:1000 |
| ARP3/ARP3B Polyclonal antibody | Proteintech | 13822-1-AP | AB_2221967 | 1:1000 |
| CD41/Integrin Alpha 2B Polyclonal antibody | Proteintech | 24552-1-AP | AB_2879604 | 1:1000 |
| GAPDH Monoclonal Antibody | Proteintech | 60004-1-Ig | AB_2107436 | 1:1000 |
| HRP-labeled Goat Anti-Rabbit IgG (H+L) | Beyotime | A0208 | AB_2892644 | 1:1000 |
